# Enantioselective Catalytic Aldol Reactions in the Presence of Knoevenagel Nucleophiles: A Chemoselective Switch Optimized in Deep Eutectic Solvents Using Mechanochemistry

**DOI:** 10.3390/molecules29010004

**Published:** 2023-12-19

**Authors:** Hanaa Al Beiruty, Sofiia-Stefaniia Zhylinska, Nino Kutateladze, Hayley Kay Tinn Cheong, José A. Ñíguez, Sarah J. Burlingham, Xavier Marset, Gabriela Guillena, Rafael Chinchilla, Diego A. Alonso, Thomas C. Nugent

**Affiliations:** 1 School of Science, Constructor University, Campus Ring 1, 28759 Bremen, Germany; 2 Department of Organic Chemistry, Institute of Organic Synthesis (ISO), University of Alicante, P.O. Box 99, 03080 Alicante, Spaingabriela.guillena@ua.es (G.G.); chinchilla@ua.es (R.C.)

**Keywords:** organic synthesis, green chemistry, organocatalysis, in-water conditions, deep eutectic solvents, aldol reaction, Knoevenagel reaction

## Abstract

In the presence of different nucleophilic Knoevenagel competitors, cyclic and acyclic ketones have been shown to undergo highly chemoselective aldol reactions with aldehydes. In doing so, the substrate breadth for this emerging methodology has been significantly broadened. The method is also no longer beholden to proline-based catalyst templates, *e.g.*, commercially available O-*t*-Bu-L-threonine is advantageous for acyclic ketones. The key insight was to exploit water-based mediums under conventional (in-water) and non-conventional (deep eutectic solvents) conditions. With few exceptions, high aldol-to-Knoevenagel chemoselectivity (>10:1) and good product profiles (yield, *dr*, and *ee*) were observed, but only in DESs (deep eutectic solvents) in conjunction with ball milling did short reaction times occur.

## 1. Introduction

Asymmetric organocatalysis is an incredibly appealing sustainable approach for the synthesis of enantiomerically pure compounds using chiral organocatalysts without the need for transition metals or enzymes [1,2,3,4]. The chiral organocatalysts used in these reactions are typically derived from natural products or designed through rational design approaches. Various methodologies have been explored to enhance the sustainability of organocatalytic processes. One such approach involves the use of alternative and environmentally friendly solvents, which helps reduce waste formation typically associated with volatile organic compounds (VOCs) used as reaction media [5,6]. Deep eutectic solvents (DESs) have lately gained attention as highly promising sustainable solvents for organic transformations [7,8,9]. These alternative solvents share characteristics with ionic liquids, such as low vapor pressure and non-flammability. However, they are not only cost-effective and easy to recycle but also have a minimal ecological impact and are simple to synthesize. Despite the numerous advantages of DESs and the significant increase in their utilization in asymmetric organocatalysis in recent years [10,11,12], their application in asymmetric organocatalyzed reactions has remained relatively limited to the typical aldol, Michael, and α-functionalization of 1,3-dicarbonyl compounds. Here, we show DESs can advantageously influence chemoselectivity.

**Scheme 1:**
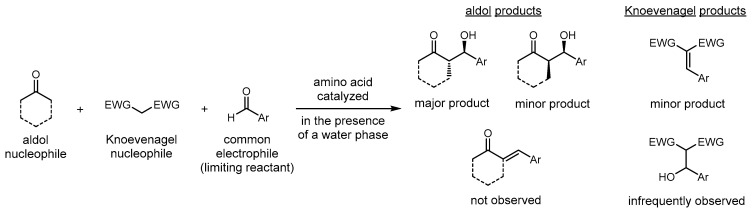
Chemoselective switch: aldol/Knoevenagel competition reactions with aldol selectivity.

Aldol and Knoevenagel condensation reactions share aldehydes as common electrophiles (Scheme 1). Knoevenagel pronucleophiles, most often methylene units flanked by two electron withdrawing groups (EWG), can be converted to their nucleophilic forms under acidic, neutral, or basic conditions [13,14]. Furthermore, their significantly lower pK_a_ values, as compared to ketones, allow them to chemoselectively undergo Knoevenagel reactions in the presence of ketones [15,16,17]. Further supportive evidence of the greater reactivity of Knoevenagel *versus* aldol (ketone) nucleophiles comes from the literature reports on the amino acid catalyzed variants. For example, the reaction of cyclohexanone or acetylacetone with benzaldehyde, under similar reaction conditions, always show Knoevenagel [18,19] reactions with significantly higher rates of reaction as compared to the corresponding aldol [20,21,22] reactions.

**Figure 1:**
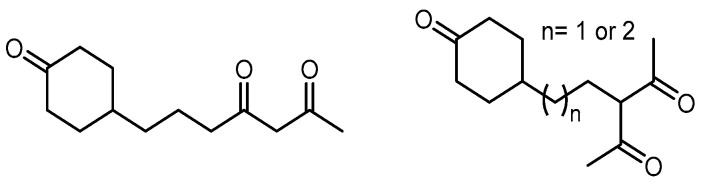
Previously studied substrates enabling chemoselective aldol *versus* Knoevenagel reactions.

To enable a chemoselective amino-acid-catalyzed aldol reaction in the presence of a Knoevenagel nucleophile, off-cycle equilibria must be suppressed, *e.g.*, beginning with catalyst deprotonation of or catalyst enamine formation with the Knoevenagel nucleophile. We recently reported [23] that employment of a water phase (in-water reactions conditions [24]) suppresses rate determining Knoevenagel intermediate accumulation, and in turn allowed a chemoselective switch wherein enantioselective aldol reactions occurred. It was further noted that the presence of water alone was not sufficient. For example, a monophasic solvent system containing water, an organic solvent, reactants, and the catalyst, only resulted in non-productive mixtures of aldol and Knoevenagel products [23]. A new chemoselectivity had been established, but the demonstrated substrate scope was narrow (Figure 1).

In this study, we present the chemo- and enantioselective organocatalyzed aldol reaction between a wide variety of ketones and aldehydes in the presence of Knoevenagel nucleophiles using water or ternary aqueous deep eutectic solvent reaction mediums. Our study not only broadens the ketone (aldol) and Knoevenagel pronucleophile scope, but importantly demonstrates how mechanochemical conditions in conjunction with deep eutectic solvent mixtures can provide dramatically shorter reaction times. Simultaneously, regio-, diastereo-, and enantioselective controls have been imparted on the aldol products. Additionally, we have conducted a comparative analysis of the outcomes obtained in deep eutectic solvents with those using in-water conditions.

## 2. Discussion and Results

**Figure 2:**
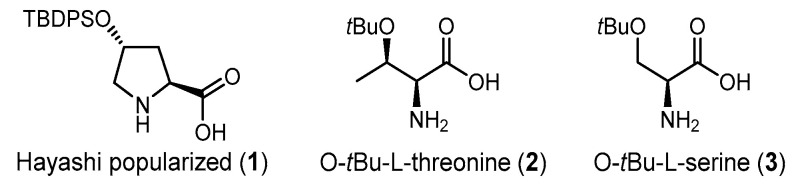
Investigated amino acid catalysts.

**Scheme 2:**
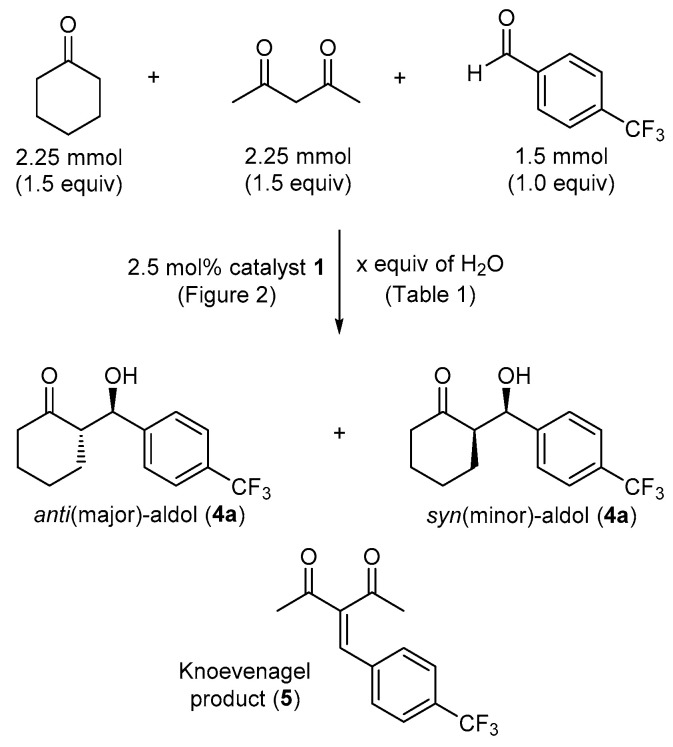
Benchmark aldol/Knoevenagel competition reaction.

Competition reactions are ideally suited for probing the chemoselective challenge at hand. To achieve that, we employed equimolar quantities of an aldol and Knoevenagel nucleophile, each competing for the limiting reactant, an aldehyde (Scheme 1), under amino acid catalysis (Figure 2). We began our study with our previously reported in-water aldol reaction conditions, which permit high aldol product stereoselectivity [23,24]. However, the optimal equivalents of water were not rigorously investigated, and we chose the competition reaction of cyclohexanone and acetylacetone for 4-(trifluoromethyl)benzaldehyde (Scheme 2) to determine this. The data (Table 1, entries 1–5) shows that as little as 3.0 equiv of water are sufficient, but we chose 15 equivalents of water for our standard protocol because it allowed more consistent and effective stirring. To further highlight the critical role of a water phase, the same reaction conditions were applied (Table 1, footnote a) albeit in dry DMSO-d_6_ (0.50 M, no added water)), but reaction conversion was held below 10% after 36 h. However, using 30 mol% of catalyst **1** in dry DMSO-d_6_ (0.70 M, no added water), the reaction proceeded to give >95% conversion with high Knoevenagel chemoselectivity (aldol/Knoevenagel = 1:17) based on *in situ* ^1^H NMR measurement (For further details, see Section S9 of the Supplementary Materials).

Physically, the employed in-water reaction conditions are heterogeneous, *i.e*., the added water is one phase while the reactants and catalyst constitute a concentrated organic phase. However, this fact raises a concern if the high chemoselectivity occurred due to the greater water solubility of the Knoevenagel *versus* aldol nucleophile. The chosen reaction (Scheme 2) uniquely addresses this hypothesis for multiple reasons which are now addressed. First, all reactants are liquids and this removes uncertainties arising from a solid providing inconsistent results due to non-uniform solubilization, *e.g.*, due to particle size, conglomeration of solids, rates of stirring, *etc*. Second, the Knoevenagel nucleophile (acetylacetone solubility: 17.1 g/100 mL H_2_O) is approximately twice as soluble in water as the aldol nucleophile (cyclohexanone solubility: 8.8 g/100 mL H_2_O). Despite this, the aldol/Knoevenagel chemoselectivity was consistent when comparing the use of 3.0 *versus* 15 equiv of water (Table 1, entries 2 (14:1) and 4 (13:1)). However, with no intentionally added water (note: enamine formation produces water during the reaction), the chemoselectivity decreased to 8:1 (Table 1, entry 1) and when 30 equiv of water were employed, the chemoselectivity was observed to be 15:1 (Table 1, entry 5).

To further examine the role of substrate solubility on chemoselectivity, water was replaced with brine (Table 1, entry 6). Interestingly, the chemoselectivity (13:1) was unchanged from the optimized protocol (entry 4) albeit with decreased aldol product *dr*. Finally, our optimized reaction conditions (Table 1, entry 4) were modified such that twice as much acetylacetone was used. This decreased the chemoselectivity to 7:1 (Table 1, entry 7), but the effect was not dramatic and provides further evidence for the strong role of a water phase in suppressing Knoevenagel reactions. In summary, for this reaction (Scheme 2), the greater water solubility of the Knoevenagel *versus* aldol nucleophile had a non-discernable effect on the chemoselectivity. Of further importance to note, where data could be located, all other Knoevenagel nucleophiles studied here are less soluble in water than cyclohexanone (see Supplementary Materials, Section S4: water solubility data for aldol and Knoevenagel nucleophiles).

With the reliability of our competition reaction established, we first investigated a variety of competition reactions (Scheme 1) using the in-water reaction conditions (Table 2 and Table 3). Successful outcomes were noted when applying the Hayashi popularized catalyst **1** to cyclic ketones (Table 2, all entries and Table 3, entry 1) while O-*t*-Bu-L-threonine (**2**) was superior to O-*t*-Bu-L-serine (**3**) and optimal for the acyclic substrate: TBS-hydroxyacetone (Table 3, entries 2–4).

Two categories of Knoevenagel competitors were examined: (i) classical: acetylacetone, diethylmalonate, and methanesulfonylacetone, and (ii) non-classical: chloroacetone and ethyl-2-phenylacetate. In addition, an aldol competitor was also examined: 4-nitroacetophenone (Table 2, entry 9). Acetylacetone is a high value Knoevenagel nucleophile to assess because it has the lowest pK_a_ value from those examined. In the event, those competition reactions provided good to high yield, *dr* and *ee* for the aldol products (Table 2 and Table 3). Notable exceptions were the competition reactions with 4-methylcyclohexanone (Table 2, entry 10), cyclopentanone (Table 2, entry 11), and TBS-hydroxyacetone (Table 3, entry 4). The first is a challenging ketone aldol substrate [24,25] and a single stereoisomer, albeit from eight possible stereoisomeric products, was isolated in only 65% yield. The second, a competition reaction between cyclopentanone/acetylacetone, proceeded with the lowest *dr* (4:1) of all in-water examples (Table 2 and Table 3) and initially displayed lower diastereoselectivity (2.3:1). It was found that higher catalyst loadings (>2.5 mol%) and longer reactions times (>24 h) negatively impacted the aldol product *dr* and may reflect catalyst induced product epimerization. In the end, a balance was struck when 2.5 mol% of catalyst **1** provided a 4:1 diastereomeric aldol ratio in 94% yield in 24 h.

The presence of alternative Knoevenagel nucleophiles, *e.g.*, diethyl malonate and methanesulfonylacetone (Table 2, entries 4 and 5), also allowed aldol good product profiles, albeit the latter suppressed the aldol product yield. Not shown or currently understood is why malononitrile provided an intractable mixture of products, and this is a current limitation.

The presence of chloroacetone and ethyl-2-phenylacetate, non-classical Knoevenagel competition substrates, readily allowed aldol product formation (Table 2, entries 6–8). However, this was not a foregone conclusion and especially so for chloroacetone, a dual threat because (i) it has been previously shown to undergo amino acid catalyzed aldol reactions [26,27] and (ii) multiple nucleophiles, including the catalyst, could attack the reactive α-halocarbon.

We next examined the tolerance of this methodology when using cyclic or acyclic α-oxygenated ketone substrates, specifically: 2,2-dimethyl-1,3-dioxan-5-one and TBS-hydroxyacetone (Table 3). Using catalyst **1** (Figure 2), we isolated a mediocre yield of the *anti*-aldol product **4g** (63%) but with excellent *dr* and *ee* (Table 3, entry 1). We then tested the acyclic substrate with a primary amine catalyst: O-*t*-Bu-L-threonine (**2**), wherein good chemoselectivity for the *syn*-aldol (major) products **4h** and **4i** was observed in the presence of acetylacetone (Table 3, entries 2 and 3) [28,29,30,31]. Because the studied aldol reactions are known (Table 2 and Table 3), no catalyst optimization was performed. Instead, literature validated aldol catalysts, specific to each ketone substrate, were used [24]. This also clarifies our abrupt change in catalyst choice (Table 3, entry 1 to 2) and why catalyst screening was not pursued. Of further general interest, acyclic ketone substrates are known to provide *anti* major aldol products under proline (and derivatives thereof) catalysis, while *syn* major aldol products are noted under primary amine catalysis [24]. Interestingly, the *syn* selective reactions provide superior *dr* and this is why we chose to investigate a primary amine catalyst (**2**) with TBS-hydroxyacetone.

The final in-water competition reaction examined an *ortho*-substituted aldehyde (Table 3, entry 4) and provided excellent chemoselectivity but unacceptably low yield (37%). Examination of the O-*t*-Bu-L-serine catalyst (**3**) provided a more deleterious result. In fact, only the chiral primary–tertiary diamine developed by Chimni has been reported to give practical product profiles when reacting this, and related, acyclic ketones with *ortho*-substituted aldehydes [29].

After successful demonstration of in-water reaction conditions for a wide variety of nucleophiles and electrophiles, a new study was carried out to observe the behavior, in terms of chemo- and stereoselectivity, of the optimized catalytic system when using deep eutectic solvents as the reaction medium. For this purpose, the aldol reaction between cyclohexanone and 4-nitrobenzaldehyde in the presence of acetylacetone and chiral organocatalyst **1** was carried out in the mixture choline chloride ChCl/urea: 2:1 at room temperature. As can be seen in Table 4 (entry 1), when the reaction was carried out employing 30 mol% of **1**, under conventional magnetic stirring conditions, a 92% conversion was observed after 36 h. Regarding chemoselectivity, the aldol/Knoevenagel ratio was 8/1 with a *dr* of 1.7/1 with a low 32% *ee* for the major *anti*-diastereoisomer. Interestingly, the reaction time could be reduced to 7 h by employing ball mill stirring [32], as well as providing a remarkable improvement in the diastereo- and enantioselectivity of the aldol reaction (entry 2). Considering the demonstrated effect that the presence of water has on the chemoselectivity of this process, the competition reaction was then studied in the ternary DES ChCl/urea/water (1:2:5.7) mixture [33]. As shown in entry 3, both the chemo- and the diastereoselectivity of the process were improved (to 15:1 and 8:1, respectively) while maintaining both the conversion (93%) and the enantioselectivity of the *anti*-isomer (96%). With respect to catalyst loading (Table 4, entries 4–7), the reaction was equally effective for this ternary eutectic mixture when reducing **1** to 5 mol% (entry 4). Under this lower catalyst loading, the absence of water (entry 5) led, as previously observed, to a lower chemo- (aldol/Knoevenagel, 3:1) and diastereoselectivity (*anti*/*syn*; 3.2:1). Similar results were observed (catalyst **1**, 5 mol%) when using an alternative DES, *e.g.*, ChCl/glycerol (1:2) provided the following profile: aldol/Knoevenagel (3.4:1), *anti*/*syn* (3:2), and 59% *ee* (*anti*-product). On the other hand, when using 2.5 and 1 mol% of **1** in ChCl/urea/water(1:2:5.7) (Table 4, entries 6 and 7), a decrease in the reaction conversion and the diastereoselectivity of the aldol process was observed. Finally, we studied the competition reaction in other aqueous ChCl/urea DES. As shown in Table 4 (entries 8 and 9) the eutectic mixtures ChCl/urea/water 1:2:2.8 and 1:2:1.4 were equally effective with respect to chemoselectivity (aldol/Knoevenagel, 15:1 and 14:1, respectively), conversion (95 and 91%, respectively) and enantioselectivity of the *anti* diastereoisomer (95 and 92% *ee*, respectively). Only for the mixture ChCl/urea/water (1:2:1.4) did a decrease in the diastereoselectivity (*anti*/*syn*) of the aldol reaction from 9:1 to 4.5:1 (compare in Table 4, entries 4 and 9) occur.

Under the optimized reaction conditions (**1** (5 mol%), ChCl/urea/water (1:2:5.7), ball-mill stirring, room temperature, 7 h) the substrate scope of the competition reaction was then examined (Table 5). Initially, cyclohexanone showed good chemoselectivities (aldol/Knoevenagel from 6:1 to 13:1) when competing for 4-nitrobenzaldehyde, 4-trifluoromethylbenzaldehyde, and methyl 4-formylbenzoate, and not only with acetylacetone but also with more activated nucleophilic methylene units such as methanesulfonylacetone (Table 5, entries 1–4). Similar results were obtained when other ketones were used, both cyclic as tetrahydro-4*H*-thiopyran-4-one (entries 5 and 6), 2,2-dimethyl-1,3-dioxan-5-one (entry 7), 4-methylcyclohexanone (entry 8), and acyclic as TBS-hydroxyacetone (entry 9) with chemoselectivities ranging between 6:1 and 32:1. Regarding the aldol reaction, high *anti*-diastereoselectivities were also obtained especially for the cyclic ketones with enantiomeric excesses between 53% and 99% for the major *anti*-isomer (Table 5). The acyclic *anti*-product (entry 9) has been previously synthesized [34].

## 3. Materials and Methods

### 3.1. General Procedure for the Preparation of the Deep Eutectic Solvents (DES)

The hydrogen bond donor and hydrogen acceptor were added to a round bottom flask in the desired molar ratios and stirred magnetically whilst heating until the formation of completely transparent solution was observed. The DES should be used within the same day to avoid degradation or any absorption of additional water.

ChCl:Urea (1:2)

Choline chloride (2.69 g, 19 mmol, 1 equiv.) and Urea (2.31 g, 38 mmol, 2 equiv.) were added to a round bottom flask and stirred magnetically at 80 °C until the formation of completely transparent solution was observed yielding approximately 5 g of DES.

ChCl:Glycerol (1:2)

Choline chloride (0.43 g, 3 mmol, 1 equiv.) and Glycerol (0.57 g, 6 mmol, 2 equiv.) were added to a round bottom flask and stirred magnetically at 80 °C until the formation of completely transparent solution was observed yielding approximately 1 g of DES.

### 3.2. Typical Competition Experimental Procedure for the Knoevenagel versus Aldol Reaction in DES

Cyclohexanone (2.37 mmol, 1.58 equiv.), 4-nitrobenzaldehyde (1.5 mmol, 1.0 equiv.), acetylacetone (2.37 mmol, 1.58 equiv.), and the chiral organocatalyst **1** were added to a 3 mL Eppendorf vial, followed by the DES (400 µL) and water (when required). The reaction was mixed in a ball mill with 5 stainless steel balls for 7h at a frequency of 15.5 s-1. Upon completion of the reaction, it was extracted with EtOAc (20 mL × 3) and water (15 mL), washed with brine (15 mL), dried over MgSO_4_ and concentrated under reduced pressure to afford the crude reaction mixture which was first analyzed by ^1^H NMR and chiral HPLC to determine the reaction conversion as well as the chemo- and stereoselectivity of the process. Finally, the crude mixture was purified by column chromatography to afford the pure compounds which were fully characterized by ^1^H NMR.

### 3.3. Typical Competition Experimental Procedure for the Knoevenagel versus Aldol Reaction in H_2_O

To a clean screw cap V-shaped reaction vessel (5.0 mL) equipped with a pyramidal stir bar, the following were added in the stated order: cyclohexanone (MW = 98.14, 1.5 equiv., 2.25 mmol, 221 mg, density = 0.947 g/mL, 233.2 µL), acetylacetone (MW = 100.12, 1.5 equiv., 2.25 mmol, 225 mg, density = 0.975 g/mL, 231 µL), deoxygenated distilled water (MW = 18.02, 15 equiv, 22.5 mmol, 405 µL), purified 4-(trifluoromethyl)benzaldehyde (MW = 174.12, 1.0 equiv, 1.5 mmol, 261 mg, density = 1.275 g/mL, 204.9 205 µL), and *trans*-4-(tert-butyldiphenylsilyloxy)-L-proline catalyst (MW = 369.54, 2.5 mol%, 0.0375 mmol, 13.9 mg). Within ten seconds, the solid catalyst fully dissolved leaving a transparent biphasic solution. The resulting heterogenous solution was rigorously stirred for 40 h such that an emulsion was always noted. Work-up entailed standard separatory funnel extractive procedures using CH_2_Cl_2_ and are found in the Supplementary Materials with additional important reaction information.

## 4. Conclusions

The goal of this research was to provide evidence for broader substrate applicability, or lack thereof, when examining a new type of chemoselectivity wherein aldol reactions prevail over Knoevenagel reactions. This was unequivocally established while demonstrating the synthesis of previously reported aldol products. Furthermore, we showed: (i) practical reaction conditions (only 1.5 equivalents of the carbonyl nucleophiles required), (ii) mediocre-to-high yields of highly enantioenriched aldol products, and (iii) short reaction times under DES/ball milling applications. The last point proved insightful for advancing this methodology because ternary aqueous deep eutectic solvent mixtures allow a water phase to coexist with the DES, while mechanochemical conditions provided effective reactant mixing. The presence of a water phase in turn suppresses rate-determining Knoevenagel intermediate accumulation and this enforces high chemoselectivity for aldol reactions. Finally, this proof-of-concept research was realized with commercially available catalysts **1**–**3**, and higher yields and/or stereoselectivity are expected when using the best-in-class organocatalysts.

## Figures and Tables

**Table 1:** Water volume: effect on chemoselectivity and stereoselectivity (Scheme 2) ^a^.

Entry	H_2_O ^b^ (equiv)	Chemoselectivity 4/5 ^c^	Yield(%) ^d^ (*anti*-&*syn*-4)	*dr* (*anti*-/*syn*-4)	*ee* (*anti*-4)
1	0	8:1	78	5:1	93
2	3.0	14:1	83	>19:1	98
3	7.5	14:1	78	>19:1	99
4	15.0	13:1	84	>19:1	98
5	30.0	15:1	79	>19:1	98
6	15.0 ^e^	13:1	87	13:1	98
7 ^f^	15.0	7:1	78	14:1	98

^a^ Standard reaction conditions: cyclohexanone (2.25 mmol, 1.5 equiv), acetylacetone (2.25 mmol, 1.5 equiv), 4-(trifluoromethyl)benzaldehyde (1.5 mmol, 1.0 equiv (limiting reactant)), catalyst **1** (2.5 mol%), 40 h. ^b^ 3.0 equiv (4.5 mmol, 81.1 µL), 7.5 equiv (11.25 mmol, 203 µL), 15 equiv (22.5 mmol, 405 µL), and 30 equiv (45 equiv, 811 µL). ^c^ Accurate chemoselectivity values were only achieved (^1^H NMR) when the entire crude reaction was dissolved in CDCl_3_. ^d^ After silica gel chromatography. ^e^ Distilled water was replaced with brine. ^f^ Double the equiv of acetylacetone (3.0 equiv, 4.5 mmol) were used.

**Table 2:** Aldol chemoselective competition reactions (Scheme 1) ^a^.

Entry	Knoevenagel Nucleophile	Major Competition Product (*anti*-4)	Aldol (4)/ Knoevenagel (5) Chemoselectivity ^b^	Time (h)	Yield(%) (*anti*-4) ^c^	*dr* (4) ^d^	*ee*(*anti*-4)
1	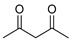	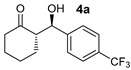	13:1	40	84 ^e^	>19:1	98
2	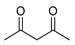	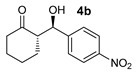	18:1	36	90 ^e^	>19:1	99
3	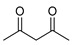	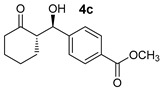	8:1	40	73	>19:1	99
4	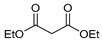	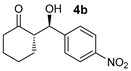	17:1	36	83	>19:1	99
5 ^f^	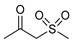	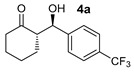	>19:1	20	61	>19:1	99
6 ^f^	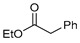	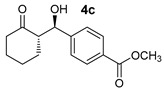	>19:1	24	83	17:1	99
7	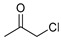	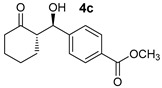	15:1	30	82	17:1	98
8 ^f^	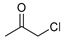	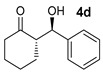	11:1	42	59	10:1	95
9 ^f^	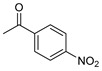	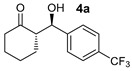	>19:1	36	82	14:1	98
10 ^f^	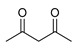	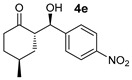	10:1	28	65	13:1 17:1 ^g^	98
11	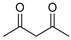	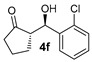	>19:1	24	94 ^e^	4:1	99

^a^ Standard reaction conditions: ketone nucleophile (2.25 mmol, 1.5 equiv), Knoevenagel nucleophile (2.25 mmol, 1.5 equiv), aldehyde (1.5 mmol, 1.0 equiv (limiting reactant)), 2.5 mol% of catalyst **1** (Figure 2), H_2_O (22.5 mmol, 405 µL, 15 equiv). ^b^ Based on crude ^1^H NMR analysis: *anti*- and *syn*-aldol products **4**/Knoevenagel product **5**. ^c^ *anti*-aldol product (single diastereomer) after silica gel chromatography. ^d^ *anti*/*syn* aldol (**4**) ratio. ^e^ Yield represents *anti*- and *syn*-aldol products **4** after silica gel chromatography. ^f^ 5.0 mol% of catalyst **1**. ^g^ Ratio of the *anti*(major)/*anti*(minor), see Supplementary Materials for structural details.

**Table 3:** Aldol chemoselective reactions using *α*-oxygenated ketones (Scheme 1) ^a^.

Entry	Knoevenagel Nucleophile	Major Competition Product (*syn*-4)	Aldol (4)/ Knoevenagel (5) Chemoselectivity ^b^	Time (h)	Yield(%) ^c^	*dr* (4) ^d^	*ee* (*syn*-4)
1 ^e^	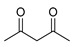	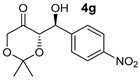	7:1	30	63 ^f^	17:1 ^g^	95 ^h^
2 ^i^	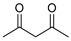	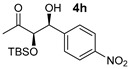	16:1	30	80	9:1	97 ^j^
3	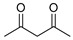	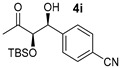	8:1	40	77	8:1	95 ^j^
4	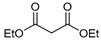	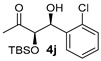	>19:1	60	37	8:1	- ^k^

^a^ Standard reaction conditions: ketone nucleophile (2.25 mmol, 1.5 equiv), Knoevenagel nucleophile (2.25 mmol, 1.5 equiv), aldehyde (1.5 mmol, 1.0 equiv (limiting reactant)), 15 mol% of catalyst **2** (Figure 2), H_2_O (22.5 mmol, 405 µL, 15 equiv). ^b^ Based on crude ^1^H NMR analysis: *syn*- and *anti*-aldol products **4**/Knoevenagel product **5**. ^c^ *syn*- and *anti*-aldol products **4** after silica gel chromatography. ^d^ *syn*/*anti* aldol (**4**) ratio. ^e^ 5.0 mol% of catalyst **1** (Figure 2) and brine (15 equiv). ^f^ Represents the *anti*-aldol product as a single diastereomer after silica gel chromatography. ^g^ *anti*(major)/*syn*(minor) aldol product. ^h^ *ee* of the *anti*-product. ^i^ H_2_O (11.3 mmol, 203 µL, 7.5 equiv). ^j^ The enantiomeric excess was determined using the O-acylated analog (Chiralpak OD-H HPLC column). ^k^ The enantiomeric excess was not determined, this aldol product and its O-acetylated derivative failed to resolve (Chiralpak OD-H HPLC column using Hex/IPA mixtures).

**Table 4:** Aldol chemoselective reaction in ChCl/Urea (1:2). Reaction optimization ^a^.

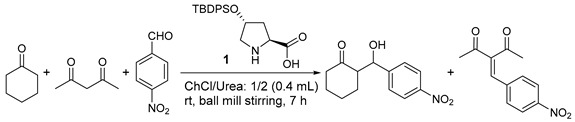					
Entry	1 (mol%)/H_2_O (μL)	Aldol (4)/Knoevenagel (5) Chemoselectivity ^b^	Conversion(%) ^b^	*dr* (*anti*/*syn-4*) ^b^	*ee* (*anti*-4) ^c^
1	30/0	8:1	92	1.7:1	32 ^d^
2	30/0	7.8:1	90	5:1	93
3	30/200 ^e^	15:1	93	8:1	96
4	5/200 ^e^	13:1	94	9:1	99
5	5/0	3:1	98	3.2:1	92
6	2.5/200 ^e^	16:1	87	8:1	93
7	1/200 ^e^	11:1	70	2:1	93
8	5/100 ^f^	15:1	95	7.5:1	95
9	5/50 ^g^	14:1	91	4.5:1	92
10	5/600 ^h^	12:1	95	7:1	93

^a^ Standard reaction conditions: cyclohexanone (1.58 equiv), acetylacetone (1.54 equiv), catalyst **1**, and 4-nitrobenzaldehyde (1.5 mmol) were added to the reaction vessel followed by the DES (400 µL). ^b^ Determined by ^1^H NMR analysis of crude reaction mixtures. ^c^ Determined by chiral HPLC analysis (97:3 Hex:IPA, OD-H, 0.6 mL/min). ^d^ Reaction performed under magnetic stirring for 36 h. ^e^ Water content: 29 wt% (200 µL). ^f^ Water content: 17 wt%. ^g^ Water content: 9 wt%. ^h^ No DES added, this volume of water mimics the total solvent volume for optimized entry 4.

**Table 5:** Aldol chemoselective reaction in ChCl/Urea (1:2). Substrate scope study ^a^.

Entry	Knoevenagel Nucleophile	Major Competition Product (4)	Aldol (4)/Knoevenagel (5) Chemoselectivity ^b^	Yield (4, %) ^c^	*dr* (4) ^b,d^	*ee* (4) ^e^
1	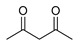	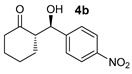	13:1	73	9:1	99
2	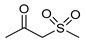	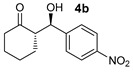	6:1	75	8.5:1.5	98
3	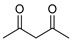	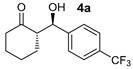	10:1	70	6.5:3.5	74 ^f^
4	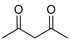	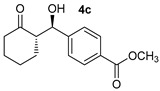	6:1	30	9:1	98
5	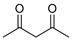	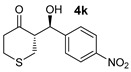	6:1	65	9.8:0.2	98 ^g^
6	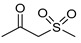	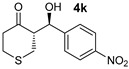	32:1	64	9.3:0.7	96
7	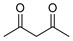	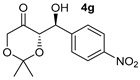	8:1	62	9:1	99 ^h^
8	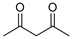	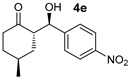	8:1	60	8.8:1.2 9:1 ^i^	94
9	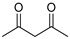	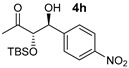	6:1	58	6:4	53 ^j^

^a^ Standard reaction conditions: ketone nucleophile (2.37 mmol, 1.58 eq.), Knoevenagel nucleophile (2.37 mmol, 1.58 eq.), aldehyde (1.5 mmol, 1.0 equiv. (limiting reactant), catalyst **1** (5 mol%), ChCl/urea/water: 1/2/5.7 (270 mg ChCl/231 mg urea/200 mg H_2_O), ball milling stirring at rt for 7 h. ^b^ Determined by crude ^1^H NMR analysis. ^c^ Isolated yield after column chromatography for the *anti*/*syn* aldol products **4**. ^d^ *anti*/*syn* aldol product (**4**) ratio. ^e^ Determined by chiral HPLC analysis (95:5 Hex:IPA, OD-H, 1 mL/min). ^f^ Determined by chiral HPLC analysis (98:2 Hex:IPA, AD-H, 1 mL/min). ^g^ Determined by chiral HPLC analysis (90:10 Hex:IPA, AD-H, 1 mL/min). ^h^ Determined by chiral HPLC analysis (90:10 Hex:IPA, OD-H, 0.75 mL/min). ^i^ *anti*(major)/*anti*(minor). ^j^ Analysis of acetylated alcohol derivative (95:5 Hex:IPA, OD-H, 0.5 mL/min).

## Data Availability

The data presented in this study are available in the article and Supplementary Material.

## Supplementary Materials

The following supporting information can be downloaded at: https://www.mdpi.com/article/10.3390/molecules29010004/s1, it contains one hundred and eleven pages of supportive information in the form of experimental details, spectra, and chromatograms. Refs. [35,36,37,38,39,40,41,42,43,44,45,46,47,48,49,50,51,52,53,54,55,56,57] are cited in supplementary materials.
