# Supplementary material for: Enantioselective Catalytic Aldol Reactions in the Presence of Knoevenagel Nucleophiles: A Chemoselective Switch Optimized in Deep Eutectic Solvents Using Mechanochemistry
Source: Molecules. 2023 Dec 19;29(1):4. doi: 10.3390/molecules29010004 (PMC10779746; doi:10.3390/molecules29010004)

Enantioselective Catalytic Aldol Reactions in the Presence of Knoevenagel Nucleophiles: A Chemoselective Switch Optimized in Deep Eutectic Solvents Using Mechanochemistry

SUPPORTING INFORMATION

Table of Contents:

Section S1. General information……………………………………………………………………………………………………2
Section S2. General competition reaction information (in-water conditions)…………………………………………2
Section S3. General competition reaction information (DES/ball milling conditions)…………………………………4
Section S4. Water solubility data for aldol and Knoevenagel nucleophiles ……………………………………………5
Section S5. Aldol racemate formation……………………………………………………………………………………………5
Section S6. Table 2 entries and characterization of *anti*-aldol products **4a**,**b**,**c**,**d**,**e**,**f**………………………………7
Section S7. Table 3 entries and characterization of *anti*-aldol product **4g** and *syn*-aldol products **4h**,**i**,**j**……67
Section S8. Table 5 entries and characterization of *anti*-aldol product **4a**,**b**,**c**,**e**,**g**,**h**,**k**……………………………99
Section S9. Knoevenagel selective competition reaction…………………………………………………………… 111

Section S1. General information

Commercial reagents were used as received, unless otherwise stated, from their commercial source, usually Sigma-Aldrich. Routine monitoring of reactions was performed by thin-layer chromatography (TLC) using precoated plates of silica gel 60 F254 and visualized under ultraviolet irradiation (254 nm) and CAM (ceric ammonium molybdate) staining solution. Column chromatography was performed using silica gel 60 (0.040-0.063 mm), and most often EtOAc with a boiling point of 77 °C and petroleum ether (pet ether) with a boiling point range of 40-60 °C were used. JEOL NMR (ECX) 400 MHz was used for the NMR analysis operating at 400 MHz (^{1}H) and 100 MHz (^{13}C) respectively. Chemical shifts (δ) were reported in parts per million (ppm) relative to $CHCl_3$ (7.26 ppm) for ^{1}H NMR and relative to $CHCl_3$ (77.16 ppm) for ^{13}C NMR. DMSO-d_6 and CD_3OD were occasionally used and then noted. Multiplicities are abbreviated as (s = singlet, d = doublet, t = triplet, q = quartet, bs = broad singlet, m = multiplet). The enantiomeric excess, *ee*, was determined using a Chiralpak OD-H chiral HPLC column (6 x 250 mm, 5 µm particle size) with mixtures of *n*-hexane (Hex) and *i*-propanol (IPA) as eluents. Solvents were delivered at a flow rate of 1.0 mL/min, the column temperature was set to room temperature, and the UV detection wave length was 254 nm unless otherwise stated.

Section S2. General competition reaction information (in-water conditions)

Standard reaction conditions: Our standard reaction conditions: ketone (aldol) nucleophile (1.5 equiv), Knoevenagel nucleophile (1.5 equiv), aldehyde (1.5 mmol, 1.0 equiv, limiting reactant), catalyst (2.5 or 5 mol% of catalyst **1** (*trans*-4-(*tert*-butyldiphenylsilyloxy)-L-proline) or 15 mol% of catalyst **2** (O-tBu-L-threonine), see Figure 2 for structure within manuscript, and water (15 equiv). Note: all solid reactants were mortar and pestle ground before weighing. Regarding reaction order, all reactions started by combining the ketone (aldol) nucleophile with the Knoevenagel nucleophile. From there, the order can be varied regarding the aldehyde, catalyst and water. *However, the addition of water was always rigorously adhered to in one of two ways: (i) after catalyst addition the water was added within 1 min, or alternatively (ii) water was added before catalyst addition.*

Reaction Screening: We intermittently screened the use of 0, 3.0, 7.5, 10, 15, and even 30 equiv of water, but 15 equiv of water was always used for the yield determination for the in-water reactions, *i.e.*, the reactions without a DES or ball milling (Tables 2 & 3). Note, in one stance we did use 7.5 equiv of water (Table 3, entry 2). Note only after we had some experience with these reactions did we observe that 15 equiv of water allowed more consistent and effective stirring. This is not a trivial point and can affect the yield. For all reactions, we used V-shaped (conical) 5.0 mL vials with matching pyramidal stir bars. Further note that some reactions had undissolved solid material (for example a reactant and/or the catalyst, while later perhaps a product) for the duration of the reaction, while at other times no solids were initially observed but later appeared (presumably product accumulation). Reactions that had undissolved or partially dissolved reactants need to be more closely observed, in most instances full conversion was still observed while in others adequate consumption was not obtained and pursuit of the reaction was abandoned. Such reactions are then ideal for examination by ball milling.

Aldehyde purity: Solid aldehydes (4-nitrobenzaldehyde, methyl 4-formylbenzoate, and 4-cyanobenzaldehyde) were used as purchases (no purification), but were always mortar and pestle ground. Liquid aldehydes are prone to oxidation on storage and were always purified immediately before addition

to the reaction. Although wasteful, we found the following procedure useful for the purification of our liquid aldehydes (all commercially purchased). Under pressurized nitrogen, about 1.5 to 2.0 mL of the neat aldehyde was passed through a disposable glass pipet (diameter ≈ 6 mm) containing a small tightly packed cotton plug, sand (0.5 cm), and silica gel (2.0 cm). The first fraction (≈ 0.5 mL) was discarded, from the next fraction (≈ 0.75 mL) was immediately taken the required aldehyde reaction volume and added to the reaction without delay. During our initial experiments, ^{13}C NMR examination of the second fraction showed no carboxylic acid was present. After those positive results, we did not examine future fractions by ^{13}C NMR even though the structure of the liquid aldehyde changed. Because of the ease of aldehyde oxidation, we recommend the use of deoxygenated distilled water.

Water (solvent): The reaction conditions represent in-water reaction conditions (Kobayashi definition, see: T. Kitanosono, S. Kobayashi, *Chem. Eur. J.* **2020**, *26*, 9408–9429.) which can be described as a concentrated organic phase and a water phase coexisting. *15 equiv of distilled water were used, but for consistent yield we recommend the use of deoxygenated distilled water.* These reactions were performed in screw capped vials (5.0 mL, when using 1.5 mmol of the aldehyde (limiting reactant)) with a head space containing atmospheric air. Despite this, the water was deoxygenated to reduce the aldehyde to carboxylic acid oxidation. Before each reaction, distilled water (250 mL) was deoxygenated using pressurized nitrogen via a fritted glass bubbling unit. The brine employed for Table 1 (entry 6) was prepared without deoxygenation of the distilled water.

Reaction monitoring: Reaction progress could not be accurately monitored by TLC due to the heterogenous nature of these reactions. Instead, screening reactions were worked-up at set times and crude ^{1}H NMR data was used to determine: aldol *versus* Knoevenagel chemoselectivity and *anti*/*syn* aldol product ratios. Crude reaction products were generally dried overnight before the ^{1}H NMR spectrum was collected. Note that sampling of the crude aldol product between cyclohexanone and 4-trifluoromethylbenzaldehyde was problematic. Each sample from the semi-solid product provided a different chemoselectivity. For that reason, the entire crude product was dissolved in $CDCl_3$ and only then did we record the ^{1}H NMR data as noted in Tables 1-3 and the experimental data within this document.

Work-up: The reaction vial contents were transferred to a beaker (100 mL) and the vial rinsed with CH_2Cl_2 (3 x 4.0 mL) and then with H_2O (2 x 3.0 mL). To the beaker was added more CH_2Cl_2 (15.0 mL) and H_2O (15.0 mL) and this biphasic solution was transferred to a separatory funnel. The beaker was rinsed several times with CH_2Cl_2 (2 x 10.0 mL), and separately with H_2O (2 x 10.0 mL), and all those washings were added to the separatory funnel. The CH_2Cl_2 layer was collected and the aqueous solution was further extracted with CH_2Cl_2 (4 x 20.0 mL). The combined organic layers were dried over Na_2SO_4, filtered, and concentrated under rotary evaporation (*the water bath must remain at room temperature, if heated, the aldol product often epimerizes and always in a less favorable direction*).

Chemoselectivity and diastereomeric ratio: Overnight high vacuum drying of the crude product removes the volatile reactants and permits the chemoselectivity and *dr* to be easily determined via the crude product ^{1}H NMR data.

Characterization: All aldol products have been previously characterized and often by many different researchers. The references we cite here are ones that showed clear experimental data in their Supporting Information and included an HPLC trace and often a ^{1}H NMR, but sometimes even that was not possible to find. At other times we have additionally offered a review to look at because there all of the best in class reaction conditions can be found for that product.

Section S3. General competition reaction information (DES/ball milling conditions)

DES preparation: The hydrogen bond donor and hydrogen acceptor were added to a round bottom flask in the desired molar ratios and stirred magnetically whilst heating until the formation of completely transparent solution was observed. The DES should be used within the same day to avoid degradation or any absorption of additional water.

ChCl:Urea (1:2)
Choline chloride (2.69 g, 19 mmol, 1 eq.) and Urea (2.31 g, 38 mmol, 2 eq.) were added to a round bottom flask and stirred magnetically at 80°C until the formation of completely transparent solution was observed yielding approximately 5 g of DES.

ChCl:Glycerol (1:2)
Choline chloride (0.43 g, 3 mmol, 1 eq.) and Glycerol (0.57 g, 6 mmol, 2 eq.) were added to a round bottom flask and stirred magnetically at 80 ºC until the formation of completely transparent solution was observed yielding approximately 1g of DES.

Standard reaction conditions in DES: ketone (aldol) nucleophile (1.5 equiv), Knoevenagel nucleophile (1.5 equiv), aldehyde (1.5 mmol, 1.0 equiv) and the chiral organocatalyst 1 were added to a 3 mL Eppendorf vial, followed by the DES (400 µL) and water (when required), in that order. The reaction was mixed in a ball mill with 5 stainless steel balls for 7h at a frequency of 15.5 s^{-1}.

Work-up for reactions in DES: the Eppendorf vial contents were transferred to a beaker (50 mL) and the vial rinsed with EtOAc (3 x 2.0 mL) and then with H2O (2 x 2.0 mL). To the beaker was added more EtOAc (15.0 mL) and H2O (15.0 mL) and this biphasic solution was transferred to a separatory funnel. The beaker was rinsed several times with EtOAc (2 x 10.0 mL), and separately with H2O (2 x 10.0 mL), and all those washings were added to the separatory funnel. The EtOAc layer was collected, and the aqueous solution was further extracted with EtOAc (3 x 20.0 mL). The combined organic layers were dried over Na2SO4, filtered, and concentrated under rotatory evaporation.

Section S4. Water solubility data for aldol and Knoevenagel nucleophiles

Table S1. Solubility data for the ketone (aldol) and Knoevenagel nucleophiles.

**Substrate**	**CRC**[1]	**HASD**[2]	**ILO ICSC**[3]	**HSDB**[4]
cyclohexanone	soluble	88.09 g/L (25 °C)	87 g/L (20 °C)	87 g/L (20 °C)
cyclopentanone	insoluble	no data	poor	9.2 g/L (estimated)
4-methylcyclohexanone	insoluble	no data	no data	no data
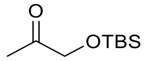	no data	no data	no data	no data
acetylacetone	very soluble	170.5 g/L (19.5 °C) 170.8 g/L (25 °C)	160 g/L (°C not specified)	166 g/L (20 °C)
diethyl malonate	slightly soluble	23.2 g/L (37°C)	20 g/L (20°C)	20 g/L (20 °C)
chloroacetone	soluble	82.57 g/L (reliability in doubt)	100 g/L (20 °C)	82.57 g/L (°C not specified)
Ethyl-2-phenylacetate	no data	1.478 g/L (25 °C)	no data	1.478 g/L (25 °C)
1-(methylsulfonyl)-2-propanone	no data	no data	no data	no data
1. CRC Handbook of Chemistry and Physics 95th Edition, 2. Handbook of Aqueous Solubility Data Second Edition, 3. ILO International Chemical Safety Cards (ICSC): www.ilo.org/dyn/icsc/showcard.home 4. Hazardous Substances Data Bank (HSDB): pubchem.ncbi.nlm.nih.gov/source/11933				

Section S5. Aldol racemate formation

The cobalt dichloride inspired methods came from the work of Reiser, see: A. Karmakar, T. Maji, S. Wittmann, O. Reiser*, Chem. Eur. J.* **2011**, *17*, 11024–11029. Note that while he calls for the use anhydrous $CoCl_2$ and MeOH, we did not always rigorously exclude moisture. Despite this we did perform these reactions under N_2. Our point, these reactions are moisture sensitive and while they tolerate some moisture, too much leads to failed reactions.

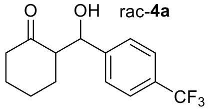
rac-**4a**

Anhydrous cobalt dichloride (MW = 129.84, 10 mol%, 0.3 mmol, 38.95 mg), L-proline (MW = 115.13, 10 mol%, 0.3 mmol, 34.54 mg), D-proline (MW = 115.13, 10 mol%, 0.3 mmol, 34.54 mg), and methanol (101.1 µL), followed by cyclohexanone (MW= 98.14, 3.0 equiv, 9.0 mmol, 883.3 mg, density= 0.947 g/mL, 932.7 µL) and 4-(trifluoromethyl)benzaldehyde (MW = 261.18, 1.0 equiv, 3.0 mmol, 783.5 mg, density= 1.275 g/mL, 614.5 µL). Time = 45 h, no yield recorded.

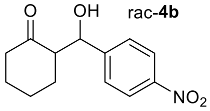
rac-**4b**

2-Picolyamine (MW= 108.14, 20 mol%, 0.53 mmol, 57.3 mg, density= 1.049 g/mL, 54.6 uL), 4-nitrobenzaldehyde (MW= 151.12, 1 equiv, 2.65 mmol, 400.5 mg), and cyclohexanone (MW= 98.14, 3 equiv, 7.95 mmol, 780.2 mg, density= 0.947 g/mL, 823.9 uL) were added to a mixture of MeOH (2.7 mL) and H_2O (2.7 mL). Time = 20 h, no yield recorded.

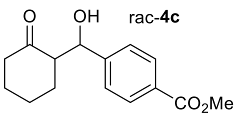

rac-**4c**

Anhydrous cobalt dichloride (MW = 129.84 g/mol, 10 mol%, 0.1 mmol, 12.9 mg), L-proline (MW = 115.13, 0.1 equiv, 0.1 mmol, 11.5 mg), D-proline (MW = 115.13, 0.1 equiv, 0.1 mmol, 11.5 mg), were added to methanol (100 µL), followed by cyclohexanone (MW = 98.14, 3.0 equiv, 3.0 mmol, density = 0.947 g/mL, 311 µL), methyl 4-formylbenzoate (MW = 164.16, 1.0 equiv, 1.0 mmol, 164.2 mg). Time = 46 h, yield = 48%.

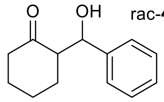

rac-**4d**

Cyclohexanone (MW= 98.14, 19.3 equiv, 19.3 mmol, 1894.1 mg, density= 0.947 g/mL, 2.0 mL), benzaldehyde (MW= 106.12, 1.0 equiv, 1.0 mmol, 106.1 mg, density= 1.044 g/mL, 101.6 µL), L-proline (MW = 115.13, 15 mol%, 0.15 mmol, 17.3 mg), D-proline (MW = 115.13, 15 mol%, 0.15 mmol, 17.3 mg) were added to DMSO (8 mL). Time = 98 h, yield = 17%.

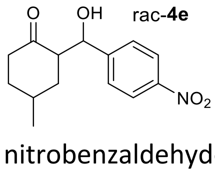

rac-**4e**

Anhydrous cobalt dichloride (MW = 129.84, 10 mol%, 0.1 mmol, 12.9 mg), L-proline (MW = 115.13, 10 mol%, 0.1 mmol, 11.5 mg), D-proline (MW = 115.13, 10 mol%, 0.1 mmol, 11.5 mg), methanol (101.1 µL), followed by 4-methylcyclohexanone (MW= 112.17, 3.0 equiv, 3.0 mmol, 336.5 mg, density= 0.914 g/mL, 368.2 µL) and 4-nitrobenzaldehyde (MW = 151.12, 1.0 equiv, 1.0 mmol, 151.1 mg). Time = 24 h, 48% yield.

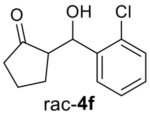

rac-**4f**

Anhydrous cobalt dichloride (MW = 129.84, 10 mol%, 0.3 mmol, 38.95 mg), L-proline (MW = 115.13, 10 mol%, 0.3 mmol, 34.54 mg), D-proline (MW = 115.13, 10 mol%, 0.3 mmol, 34.54 mg), and methanol (101.1 µL), followed by cyclopentanone (MW= 84.12, 3.0 equiv, 9.0 mmol, 757.1 mg, density= 0.951 g/mL, 796.1 µL) and 2-chlorobenzaldehyde (MW = 140.57, 1.0 equiv, 3.0 mmol, 421.7 mg, density= 1.248 g/mL, 337.9 µL). Time = 45 h, no yield recorded.

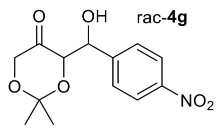

rac-**4g**

Anhydrous cobalt dichloride (MW = 129.84, 10 mol%, 0.1 mmol, 12.8 mg), L-proline (MW = 115.13, 0.1 mmol, 11.5 mg), D-proline (115.13, 0.1 mmol, 11.5 mg), and methanol (101 µL), followed by 2,2-dimethyl-1,3-dioxan-5-one (MW = 130.14, 3.0 mmol, 358 µL) and 4-nitrobenzaldehyde (MW = 151.12, 1.00 mmol, 151.12 mg). Time = 96 h, yield = 51%.

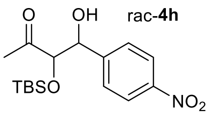

rac-**4h**

1-(t -butydimethylsilyloxy)-2-propanone (MW= 188.34, 4.0 equiv, 6.00 mmol, density= 0.976 g/mL, 1157.3 µL), O-tBu-L-serine (MW= 161.20, 15 mol%, 0.225 mmol, 36.3 mg), O-tBu-D-serine (MW= 161.20, 15 mol%, 0.225 mmol, 36.3 mg) and 4-nitrobenzaldehyde (MW= 151.12, 1.0 equiv, 1.5 mmol, 226.7 mg) followed by distilled water (10.0 equiv, 15.0 mmol, 270.3 µL). Time = 96 h, no yield recorded.

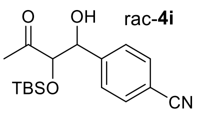

rac-**4i**

1-(t-butydimethylsilyloxy)-2-propanone (MW= 188.34, 4.0 equiv, 6.00 mmol, density= 0.976 g/mL, 1157.3 µL), O-tBu-L-serine (MW= 161.20, 15 mol%, 0.225 mmol, 36.27 mg), O-tBu-D-serine (MW= 161.20, 15 mol%, 0.225 mmol, 36.27 mg) and 4-cyanobenzaldehyde (MW= 131.13, 1.0 equiv, 1.5 mmol, 196.69 mg), followed by distilled water (10.0 equiv, 15.0 mmol, 270.3 µL). Time = 1 week, no yield recorded.

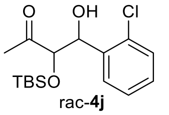

rac-**4j**

1-(t-butydimethylsilyloxy)-2-propanone (MW= 188.34, 4.0 equiv, 6.00 mmol, density= 0.976 g/mL, 1157.3 µL), O-tBu-L-serine (MW= 161.20, 15 mol%, 0.225 mmol, 36.27 mg), O-tBu-D-serine (MW= 161.20, 15 mol%, 0.225 mmol, 36.27 mg)and 2-chlorobenzaldehyde (MW= 140.57, 1.0 equiv, 1.5 mmol, 168.95 mg), followed by distilled water (10.0 equiv, 15.00 mmol, 270.3 µL). Time = 1 week, no yield recorded.

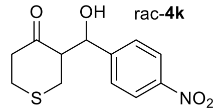

rac-**4k**

Anhydrous cobalt dichloride (MW = 129.84, 10 mol%, 0.3 mmol, 38.95 mg), L-proline (MW = 115.13, 10 mol%, 0.3 mmol, 34.54 mg), D-proline (MW = 115.13, 10 mol%, 0.3 mmol, 34.54 mg), and methanol (101.1 µL), followed by tetrahydro-4H-thiopyran-4-one (MW= 116.18, 3.0 equiv, 9.0 mmol, 1.045 g) and 4-nitrobenzaldehyde (MW= 151.12, 1 equiv, 3 mmol, 453.3 mg). Time = 48 h, no yield recorded.

Section S6. Table 2 entries & characterization of *anti*-aldol products **4a**, **b**, **c**, **d**, **e**, **f**

Table 2, entry 1: Competition reaction between cyclohexanone and acetylacetone for the limiting reactant 4-(trifluoromethyl)benzaldehyde

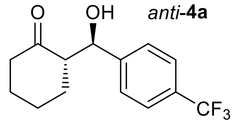

anti-**4a**

(S)-2-((R)-hydroxy(4-(trifluoromethyl)phenyl)methyl)cyclohexan-1-one

To a clean screw cap V-shaped reaction vessel (5.0 mL) equipped with a pyramidal stir bar, were added cyclohexanone (MW= 98.14, 1.5 equiv, 2.25 mmol, 221 mg, density= 0.947 g/mL, 233 µL), acetylacetone (MW= 100.12, 1.5 equiv, 2.25 mmol, 225.3 mg, density= 0.975 g/mL, 231 µL), deoxygenated distilled water (MW= 18.02, 15 equiv, 22.5 mmol, 405 µL), purified 4-(trifluoromethyl)benzaldehyde (MW= 174.12, 1.0 equiv, 1.5 mmol, 261.2 mg, density= 1.275 g/mL, 205 µL), and *trans*-4-(*tert*-butyldiphenylsilyloxy)-L-proline catalyst (MW= 369.54, 2.5 mol%, 0.0375 mmol, 13.9 mg) in the stated order. Within ten seconds the solid catalyst fully dissolved leaving a transparent biphasic solution. The resulting heterogenous solution was rigorously stirred for 40 h such that an emulsion was always noted, albeit without causing splashing against the vessel walls, by doing so reproducible yield data was achieved. This competition reaction was also used during a larger study examining what role the volume of water had on the chemoselectivity of the reaction (**Scheme 2** & **Table 1** of the manuscript). All **Table 1** reactions were set up in the same manner and with the same order of addition as noted here.

This compound has been synthesized by many research groups, one example is:
Miura, T.; Imai, K.; Ina, M.; Tada, N.; Imai, N.; Itoh, A. Direct asymmetric aldol reaction with recyclable fluorous organocatalyst. *Org. Lett.* **2010**, *12*, 1620–1623.

Crude product ^{1}H NMR analysis (**Figure S1** below) allowed the following diastereo- and chemoselectivity ratios to be determined. Note: For the crude ^{1}H NMR data, taking a random small portion of the crude semi-solid reaction product of this reaction did not result in reproducible data, thus the entire crude reaction product was dissolved in $CDCl_3$ and then the ^{1}H NMR data was collected. That is, in our hand, when examining a random sample of these crude reaction products, a solid/simultaneously viscous oil, a different chemoselectivity was noted each time.

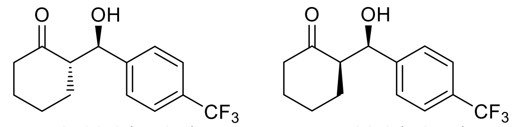

anti-aldol (major) *syn*-aldol (minor)

Diastereoselectivity: The *anti*/*syn* aldol ratio was determined as >19:1 by comparing the *anti*-aldol product resonance at 4.85 ppm (d, representing the benzylic proton) and the *syn*-aldol product resonance at 5.44 ppm (broad singlet, representing the benzylic proton). For literature regarding the *syn*-aldol product chemical shift, see page 14 of the Supporting Information from the following reference: J. Gao, S. Bai, Q. Gao, Y. Liu, Q. Yang, *Chem. Commun.* **2011**, *47*, 6716-6718.

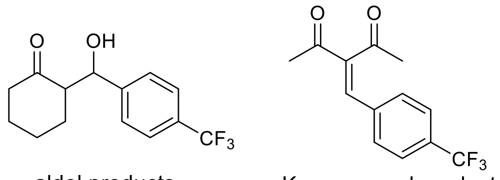

aldol products Knoevenagel product

Chemoselectivity: The aldol/Knoevenagel product ratio was determined to be 13.1:1 based on the addition of the *anti*-aldol product resonance at 4.85 ppm (the doublet representing the benzylic proton) and the *syn*-aldol product resonance at 5.44 ppm (broad singlet, representing the benzylic proton) *versus* the Knoevenagel product resonance at 2.28 ppm (singlet representing one of the methyl groups). The Knoevenagel condensation product for acetylacetone and 4-(trifluoromethyl)benzaldehyde is not reported in the literature; however, the compound was isolated as a minor impurity and the semi-purified ^{1}H NMR spectrum can be seen below in **Figure S3**.

Purification and yield: Silica gel chromatography (35 mm column outer diameter, 16 cm silica gel bed height) was performed using gradient elution (1-2 vol% acetone in CH_2Cl_2). The crude product was loaded onto the column in a minimum volume of dichloromethane. The purified *anti*-aldol product **4a** was isolated as a single diastereomer as a light-yellow solid (MW= 272.27, 340.8 mg, 1.25 mmol, 84% yield).

TLC: R_f=0.44, acetone/CH_2Cl_2 (1:49)

98% *ee*: Chiralpak OD-H chiral HPLC column, iPrOH/n-hexane (20:80), flow rate = 0.5 mL/min, λ= 216 nm, *anti*-aldol product t_{major}= 11.2 min, t_{minor}= 13.8 min (**Figure S5**).

^{1}H NMR (400 MHz, $CDCl_3$) (ppm) *anti*-aldol product **4a**: δ 7.61 (d, 2H, J= 8.6 Hz), 7.44 (d, 2H, J= 8.6 Hz), 4.85 (d, 1H, J= 8.6 Hz), 2.54-2.64 (m, 1H), 2.44-2.53 (m, 1H), 2.30-2.42 (m, 1H), 2.05-2.16 (m, 1H), 1.76-1.87 (m, 1H), 1.48-1.74 (m, 3H), 1.27-1.41 (m, 1H)

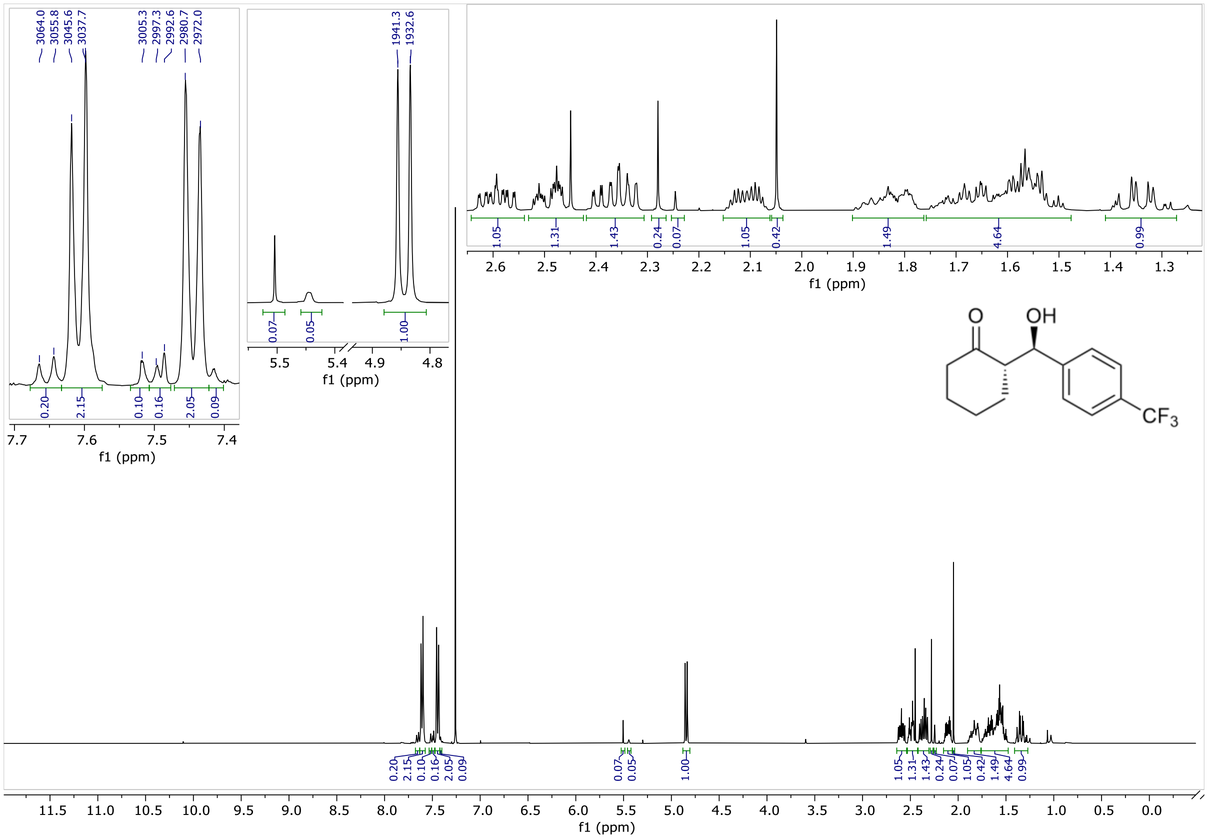

Figure S1. Crude ^{1}H NMR spectrum after high vacuum drying overnight, when employing 15 equiv of H_2O (above).

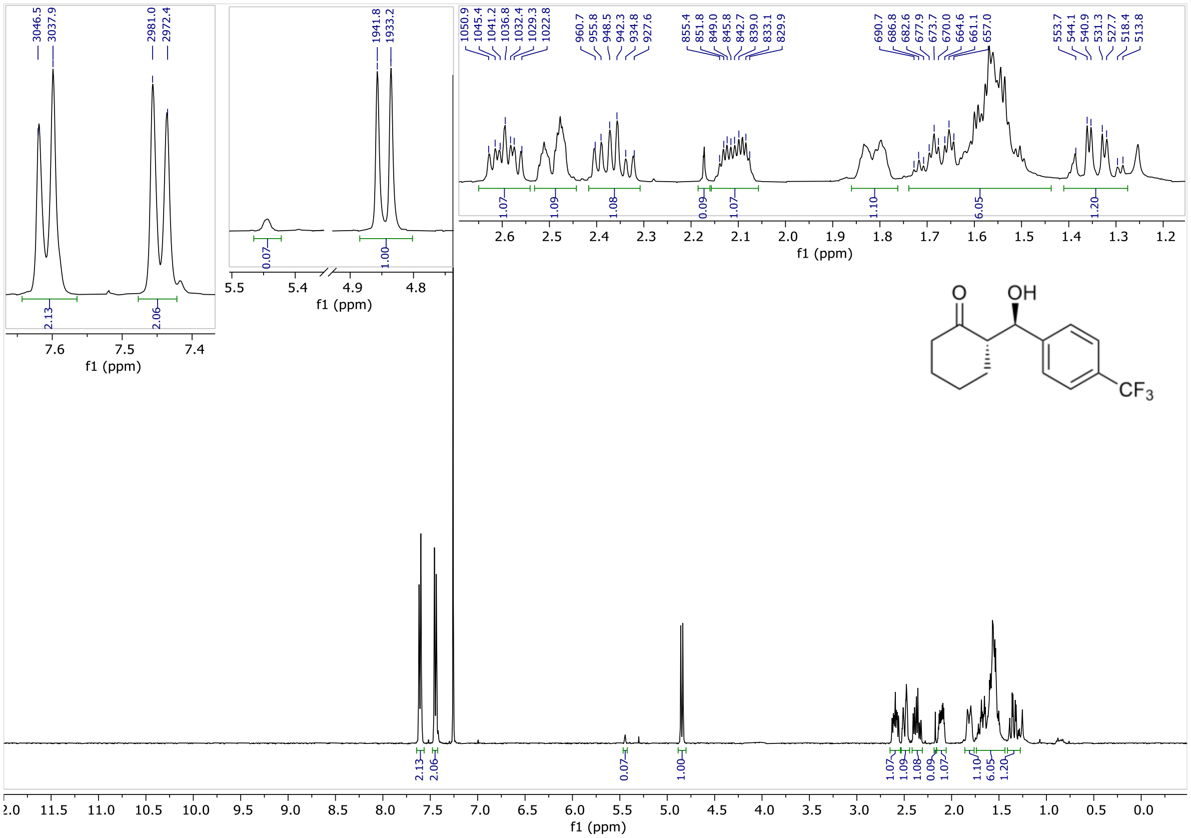

Figure S2. ^{1}H NMR spectrum of the purified *anti*-(major) and *syn*-(minor) aldol products (above), when employing 15 equiv of H_2O (above).

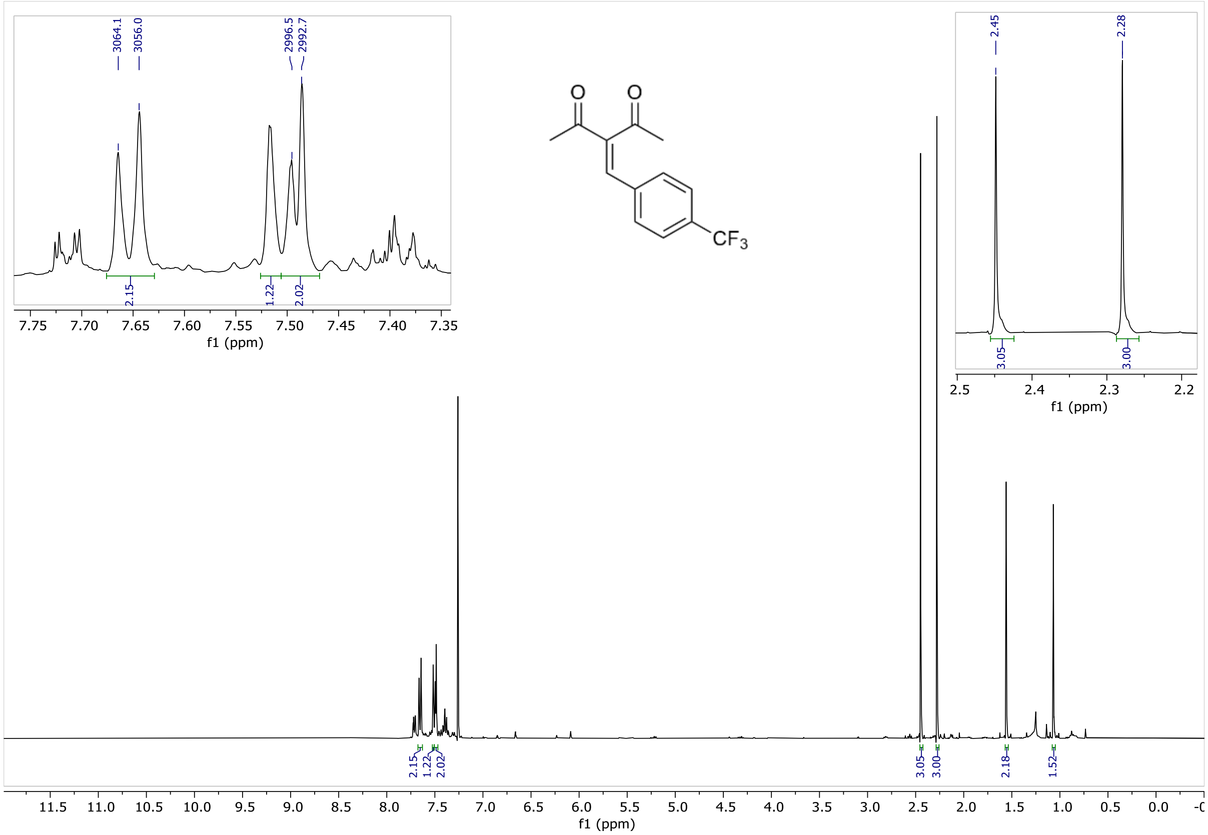

Figure S3. Semi-purified Knoevenagel condensation product (above).

SHIMADZU LabSolutions

Analysis Report

<Sample Information>

Sample Name	: HC-I-26 20%IPAnHex 20uL 0.5 mLmin 08Feb23 1mgmL		
Sample ID	:		
Data Filename	: HC-I-26 20%IPAnHex 20uL 0.5 mLmin 08Feb23 1mgmL.lcd		
Method Filename	: trial.lcm		
Batch Filename	:		
Vial #	: 1-4	Sample Type	: Unknown
Injection Volume	: 20 uL		
Date Acquired	: 2/8/2023 3:15:20 PM	Acquired by	: System Administrator
Date Processed	: 2/8/2023 3:39:35 PM	Processed by	: System Administrator

<Chromatogram>

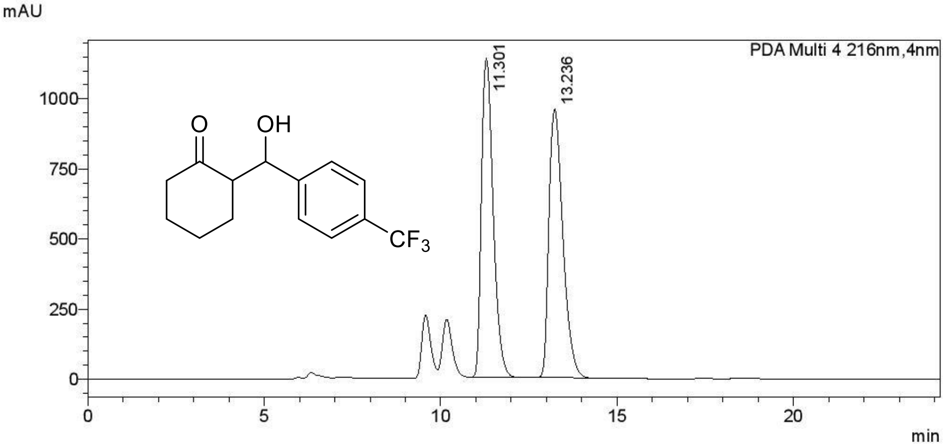

<Peak Table>

PDA Ch4 216nm

Peak#	Ret. Time	Area	Height	Area%
1	11.301	26277677	1138787	49.940
2	13.236	26341028	958736	50.060
Total		52618705	2097523	100.000

C:\Users\Shimadzu\Desktop\Hayley\HC-I-26 20%IPAnHex 20uL 0.5 mLmin 08Feb23 1mgmL.lcd

Figure S4. HPLC trace of racemic aldol product **4a** (above).

SHIMADZU LabSolutions **Analysis Report**

<Sample Information>

Sample Name : HAB-I64 15equiv 20%IPAnHex 20ul 0.5mLmin 05May23
Sample ID :
Data Filename : HAB-I64 15equiv 20%IPAnHex 20ul 0.5mLmin 05May23.lcd
Method Filename : trial.lcm
Batch Filename :
Vial # : 1-5 Sample Type : Unknown
Injection Volume : 20 uL
Date Acquired : 5/5/2023 4:11:25 PM Acquired by : System Administrator
Date Processed : 5/5/2023 4:32:47 PM Processed by : System Administrator

<Chromatogram>

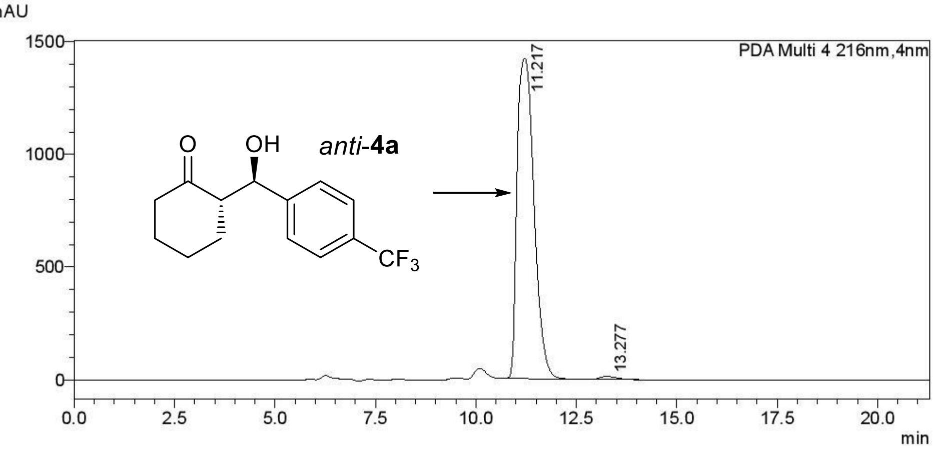

<Peak Table>

PDA Ch4 216nm

Peak#	Ret. Time	Area	Height	Area%
1	11.217	41633026	1420775	99.104
2	13.277	376319	12839	0.896
Total		42009345	1433614	100.000

C:\Users\Shimadzu\Desktop\Hanaa\HAB-I64 15equiv 20%IPAnHex 20ul 0.5mLmin 05May23.lcd

Figure S5. HPLC trace of the enantioenriched *anti*-aldol (major) product **4a.** (15 equiv water, above).

Table 2, entry 2: Competition reaction between cyclohexanone and acetylacetone for the limiting reactant 4-nitrobenzaldeyhde

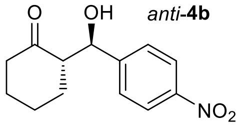
anti-**4b**

(*S*)-2-[(*R*)-hydroxy(4-nitrophenyl)methyl]cyclohexanone

To a clean screw cap V-shaped reaction vessel (5.0 mL) equipped with a pyramidal stir bar, were added cyclohexanone (MW= 98.14, 1.5 equiv, 2.25 mmol, density= 0.947 g/mL, 233 µL), acetylacetone (MW= 100.12, 1.5 equiv, 2.25 mmol, density= 0.975 g/mL, 231 µL), mortar and pestle ground 4-nitrobenzaldehyde (MW= 151.12, 1.0 equiv, 1.50 mmol, 227 mg) and *trans*-4-(*tert*-butyldiphenylsilyloxy)-L-proline catalyst (MW= 369.54, 2.5 mol%, 0.0375 mmol, 13.9 mg) in the stated order. Within a minute of adding the catalyst, distilled water (MW= 18.02, 15.0 equiv, 22.5 mmol, 405 µL) was added. The resulting heterogenous solution was rigorously stirred for 36 h. In general, the solid reactants/catalyst were fully dissolved between 60-90 min. The reaction solution was efficiently stirred albeit without causing the contents to splash against the vessel walls, by doing so reproducible yield data was achieved.

This compound has been synthesized by many research groups, one example is:
Yang, Y.; Wang, C.; Cheng, Q.; Su, Y.; Li, H.; Xiao, R.; Tan, C.; Liu, G. A Chemo-enzymatic oxidation/aldol sequential process directly converts arylbenzyl alcohols and cyclohexanol into chiral β-hydroxy carbonyls. *Green Chem.* **2021**, *23*, 7773–7779.

Crude product ^{1}H NMR analysis (**Figure S6** below) allowed the following diastereo- and chemoselectivity ratios to be determined.

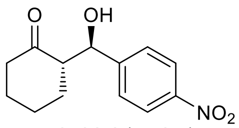
anti-aldol (major)

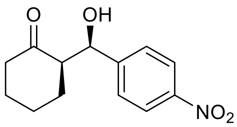
syn-aldol (minor)

Diastereoselectivity: The *anti*/*syn* aldol product ratio was determined to be >19:1, based on the *anti*-aldol product resonance at 4.90 ppm (d, representing the benzylic proton) and the *syn*-aldol product resonance at 5.48 ppm (d, representing the benzylic proton). The optimized reaction reported here was repeated three times, and the *anti*-aldol/*syn*-aldol product ratio ranged from 17:1 to 25:1. For literature regarding the *syn*-aldol product chemical shift, see page 2 of the Supporting Information within the following reference: N. Mase, Y. Nakai, N. Ohara, H. Yoda, K. Takabe, F. Tanaka, C. F. Barbas III, *J. Am. Chem. Soc.* **2006**, *128*, 734–735.

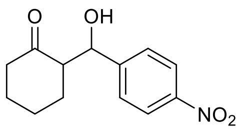
aldol products

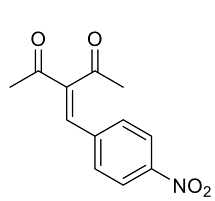
Knoevenagel product

Chemoselectivity: The aldol/Knoevenagel product ratio was determined to be 17.5:1, based on the addition of the *anti*-aldol product resonance at 4.90 ppm (d, representing the benzylic proton) and *syn*-aldol product resonance at 5.48 ppm (d, representing the benzylic proton) *versus* the Knoevenagel product resonance at 2.29 ppm (s, representing one of the methyl groups). For literature regarding the Knoevenagel condensation product chemical shift, see page 2258 of the following reference: Y. Zhang, C. Sun, J. Liang, Z. Shang, *Chin. J. Chem*. **2010**, *28*, 2255-2259.

Purification and yield: Silica gel chromatography (35 mm column outer diameter, 16 cm silica bed height) was performed by adding the crude product in a minimum volume of CH_2Cl_2. Gradient elution was used (1 to 5 vol% acetone in dichloromethane). 335.6 mg (MW= 249.27, 1.35 mmol, 90% yield) of the *anti-/syn-*aldol products **4b** were isolated as a light-yellow solid. Note that EtOAc/pet ether eluent systems did not allow chromatic removal of the Knoevenagel product.

TLC: R_f = 0.39, acetone/dichloromethane (1:49).

99% *ee*: Chiralpak OD-H chiral HPLC column, *i*PrOH/*n*-hexane (7:93), flow rate = 1.0 mL/min, λ= 254 nm, *anti*-aldol product t_{major}= 19.3 min, t_{minor}= 27.2 min was observed (**Figure S9**).

^{1}H NMR (400 MHz, $CDCl_3$) (ppm) *anti*-aldol product **4b**: δ 8.21 (d, 2H, J= 8.9 Hz), 7.51 (d, 2H, J= 8.3 Hz), 4.90 (d, 1H, J= 8.4 Hz), 4.07 (br, s, 1H), 2.54-2.64 (m, 1H), 2.45-2.54 (m, 1H), 2.31-2.42 (m, 1H), 2.07-2.16 (m, 1H), 1.77-1.89 (m, 1H), 1.49-1.76 (m, 3H), 1.31-1.45 (m, 1H).

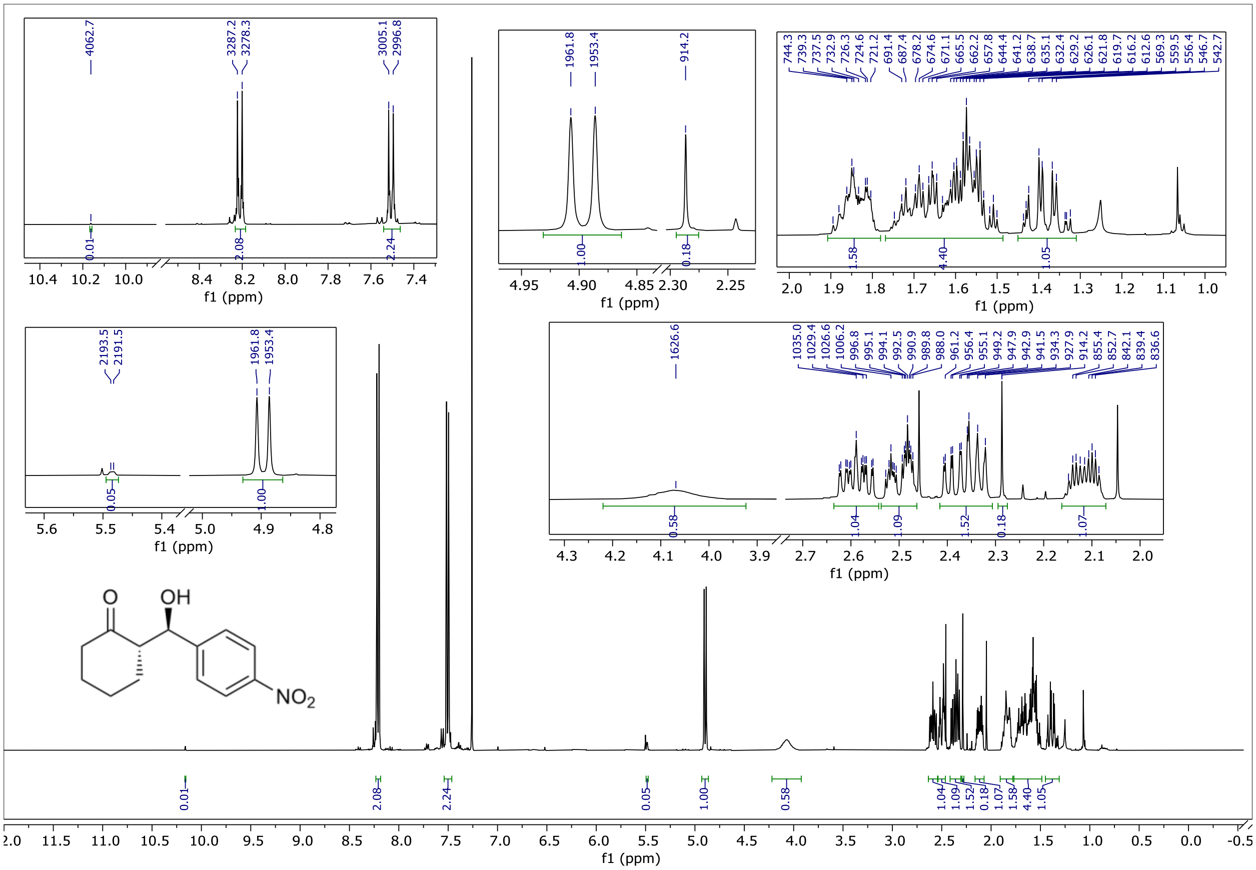

Figure S6. Crude ^{1}H NMR spectrum after high vacuum drying overnight (above).

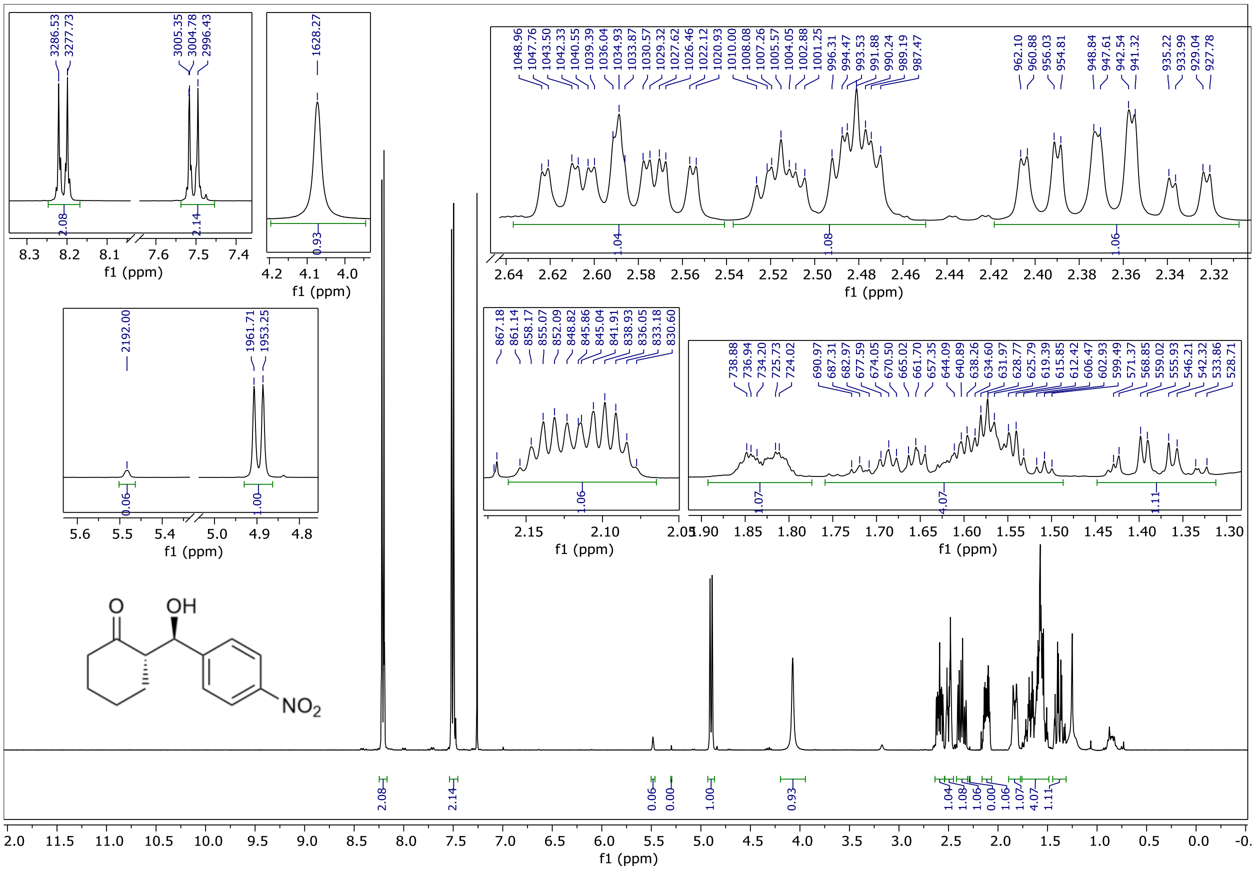

Figure S7. ^{1}H NMR spectrum of the purified *anti*-(major) and *syn*-(minor) aldol products (above).

SHIMADZU LabSolutions

Analysis Report

ADV-A45-7% IPA/nHex 5uL 1mL/min 12May2022

Sample Name	: A46 (RACE)7% IPAnHex 5uL1mLmin 26May 2
Sample ID	:
Data Filename	: A46 (RACE)7% IPAnHex 5uL1mLmin 26May 2.lcd
Method Filename	: trial.lcm
Batch Filename	:
Vial #	: 1-1
Sample Type	: Unknown
Injection Volume	: 20 uL
Date Acquired	: 5/26/2022 2:38:31 PM
Acquired by	: System Administrator
Date Processed	: 5/26/2022 3:28:36 PM
Processed by	: System Administrator

ADV-A46-7% IPA/nHex 5uL 1mL/min 12May2022

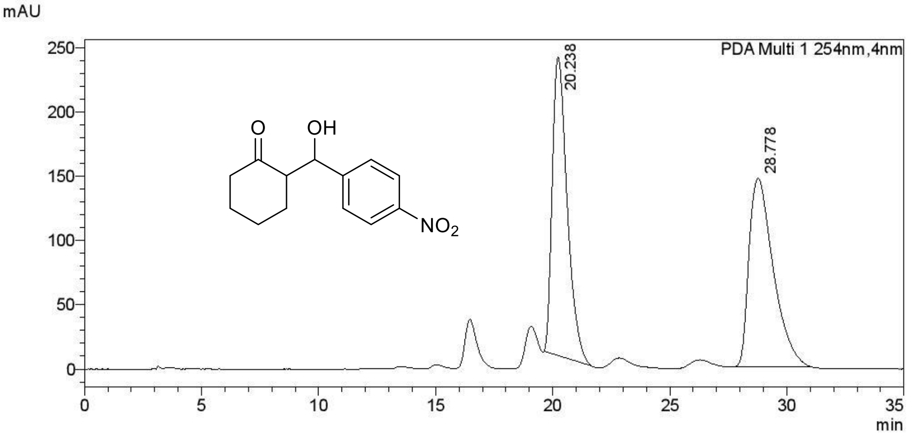

ADV-A45-7% IPA/nHex 5uL 1mL/min 12May2022

PDA Ch1 254nm

Peak#	Ret. Time	Area	Height	Area%
1	20.238	10283216	232276	49.316
2	28.778	10568658	146536	50.684
Total		20851874	378812	100.000

C:\Users\Shimadzu\Desktop\ADV\Competition aldol samples\A46 (RACE)7% IPAnHex 5uL1mLmin 26May 2.lcd

Figure S8. HPLC trace of racemic aldol product **4b** (above).

SHIMADZU LabSolutions **Analysis Report**

<Sample Information>

Sample Name	: HC-I-9-36h 7%IPAnHex 20uL 1mLmin 2nd run 12Aug
Sample ID	:
Data Filename	: HC-I-9-36h 7%IPAnHex 20uL 1mLmin 2nd run 12Aug.lcd
Method Filename	: trial.lcm
Batch Filename	:
Vial #	: 1-4
Sample Type	: Unknown
Injection Volume	: 20 uL
Date Acquired	: 8/12/2022 4:28:17 PM
Acquired by	: System Administrator
Date Processed	: 8/12/2022 5:03:37 PM
Processed by	: System Administrator

<Chromatogram>

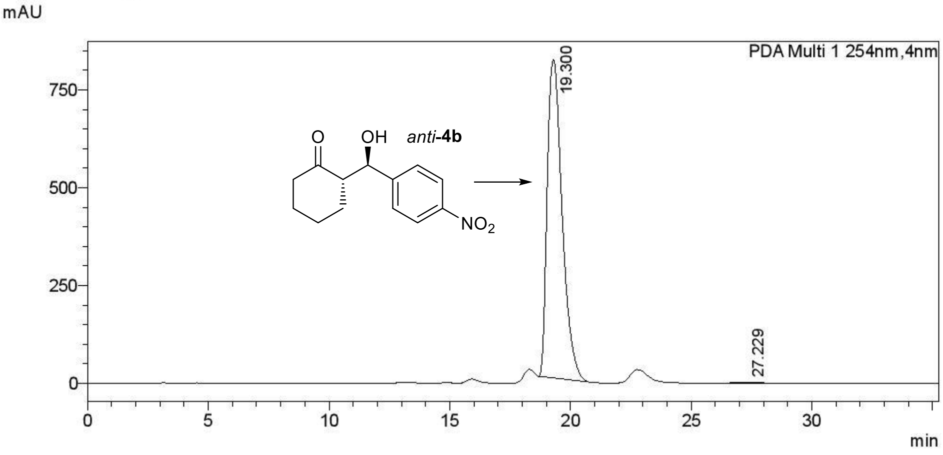

<Peak Table>

PDA Ch1 254nm

Peak#	Ret. Time	Area	Height	Area%
1	19.300	34906308	814542	99.565
2	27.229	152416	2741	0.435
Total		35058724	817282	100.000

C:\Users\Shimadzu\Desktop\Hayley\HC-I-9-36h 7%IPAnHex 20uL 1mLmin 2nd run 12Aug.lcd

Figure S9. HPLC trace of the enantioenriched *anti*-aldol (major) product **4b** (above).

Table 2, entry 3: Competition reaction between cyclohexanone and acetylacetone for the limiting reagent methyl 4-formylbenzoate

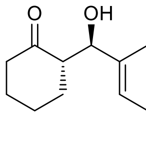
anti-**4c**

Methyl 4-((*R*)-hydroxy((*S*)-2-oxocyclohexyl)methyl)benzoate

To a clean screw cap V-shaped reaction vessel (5.0 mL) equipped with a pyramidal stir bar, were subsequently added cyclohexanone (MW = 98.14, 1.50 equiv, 2.25 mmol, density = 0.947 g/mL, 233 µL), acetylacetone (MW= 100.12, 1.50 equiv, 2.25 mmol, density = 0.975 g/mL, 231 µL), *trans*-4-(tert-butyldiphenylsilyloxy)-L-proline catalyst (MW = 369.54, 2.5 mol%, 0.01875 mmol, 13.9 mg) and mortar and pestle ground methyl 4-formylbenzoate (MW = 164.16 g/mol, 1.00 equiv, 1.50 mmol, 246.24 mg) in the stated order. Within a minute of adding the catalyst, deoxygenated distilled water (MW = 18.02, 15.0 equiv, 22.5 mmol, 405 µL) was added. The resulting yellow heterogenous solution was rigorously stirred for 40 h. The reaction solution was efficiently stirred albeit without causing the contents to splash against the vessel walls, by doing so reproducible yield data was achieved.

This compound has been synthesized by many research groups, one example is:
Inoue, H.; Kikuchi, M.; Ito, J.-i.; Nishiyama, H. Chiral phebox-rhodium complexes as catalysts for asymmetric direct aldol reaction. *Tetrahedron* **2008**, *64*, 493-499.

Crude product ^{1}H NMR analysis (**Figure S10**) allowed the following diastereo- and chemoselectivity ratios to be determined.

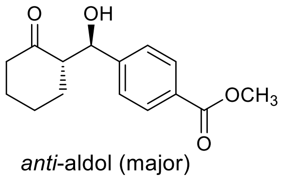
anti-aldol (major)

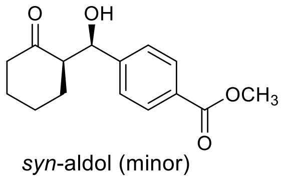
syn-aldol (minor)

Diastereoselectivity: The *anti*-aldol/*syn*-aldol product ratio was determined as >19:1, based on the *anti*-aldol product resonance at 4.84 ppm (d representing the benzylic proton) and the *syn*-aldol product resonance at 5.45 ppm (bs, representing the benzylic proton). For literature regarding the *syn*-aldol product chemical shift, see page 2, product **3c**, within the Supporting Information of: N. Mase, Y. Nakai, N. Ohara, H. Yoda, K. Takabe, F. Tanaka, C. F. Barbas III, *J. Am. Chem. Soc.* **2006**, *128*, 734–735.

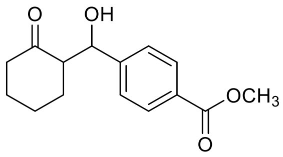
aldol products

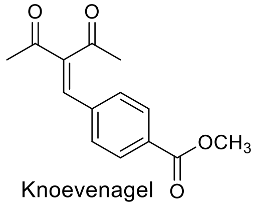
Knoevenagel product

Chemoselectivity: The aldol/Knoevenagel product ratio was determined to be 7.5:1, based on the addition of the *anti*-aldol product resonance at 4.84 ppm (d, representing the benzylic proton) and the *syn*-aldol product resonance at 5.45 ppm (bs, representing the benzylic proton) *versus* the Knoevenagel product resonance at 2.27 ppm (s, representing one of the methyl groups). The Knoevenagel condensation product was also isolated, see **Figure S12** below.

Purification and yield: Silica gel chromatography (35 mm column outer diameter, 16 cm silica bed height) was performed using gradient elution (1.5 to 3.5 vol% acetone in CH_2Cl_2) to remove the starting material, the Knoevenagel condensation product, and the *syn*-aldol product. The crude product was loaded onto the column in a minimum volume of CH_2Cl_2. The *anti*-aldol product **4c** was isolated as a single diastereomer as a light yellow solid (285.1 mg, MW = 262.31, 1.09 mmol, 73% yield).

TLC: R_f = 0.29, acetone/CH_2Cl_2 (1:39)

99% *ee*: Chiralpak OD-H chiral HPLC column, n-hexane/iPrOH (95:5), flow rate = 1.0 mL/min, λ = 254 nm, *anti*-aldol product t_{major}= 19.4 min, *syn*-aldol product t_{minor}= 22.8 min retention times were observed (**Figure S14**).

^{1}H NMR (400 MHz, $CDCl_3$) (ppm) *anti*-aldol product **4c**: δ 8.01 (d, 2H, J = 8.3 Hz), 7.39 (d, 2H, J = 8.4 Hz), 4.84 (dd, 1H, J = 8.6, 2.8 Hz), 4.03 (d, 1H, J = 2.9 Hz), 3.93 (s, 3H), 2.59 (ddd, 1H, J = 13.8, 8.7, 5.4 Hz), 2.48 (dm, 1H, J = 13.6 Hz), 2.36 (ddd, 1H, J = 13.3, 6.1 Hz), 2.10 (m, 1H), 1.79 (dm, 1H, J = 11 Hz), 1.62 (m, 3H), 1.32 (ddt, 2H, J = 13.6, 3.8, 1.4 Hz).

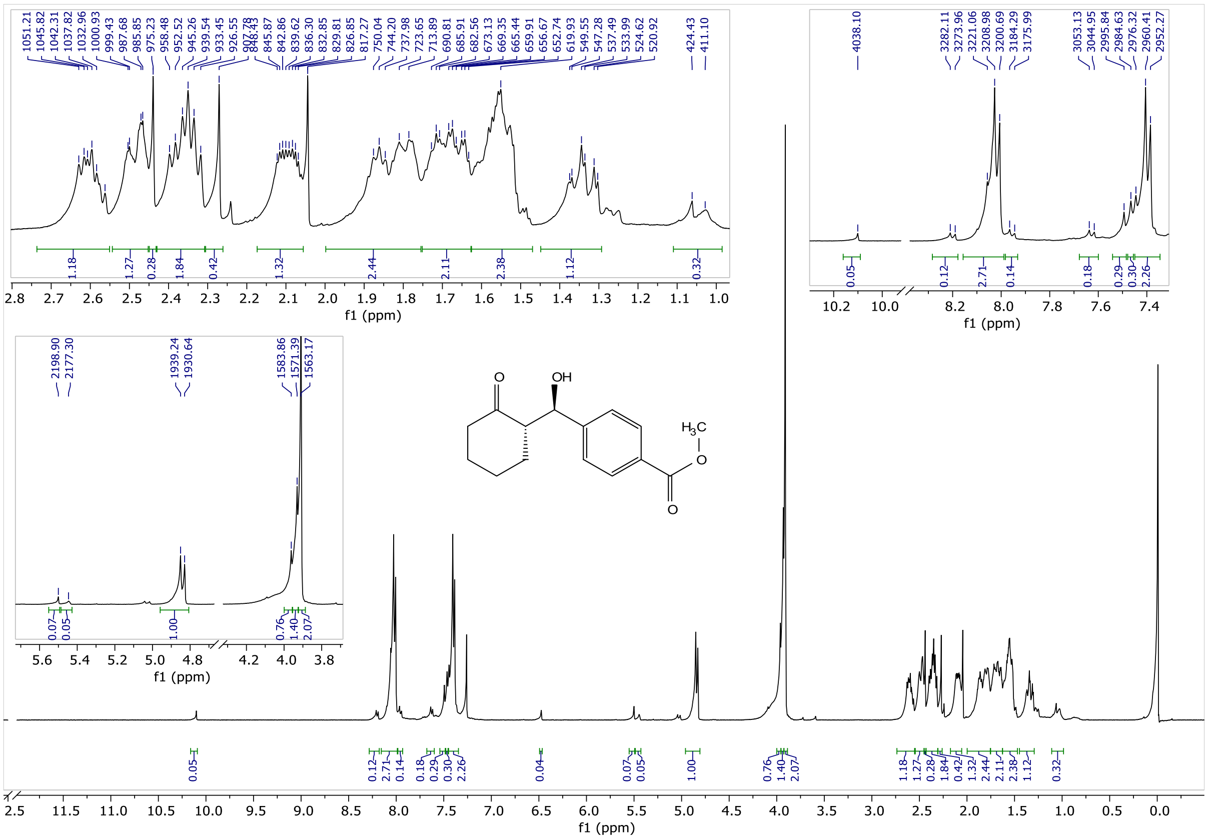

Figure S10. Crude ^{1}H NMR spectrum after high vacuum drying overnight (above).

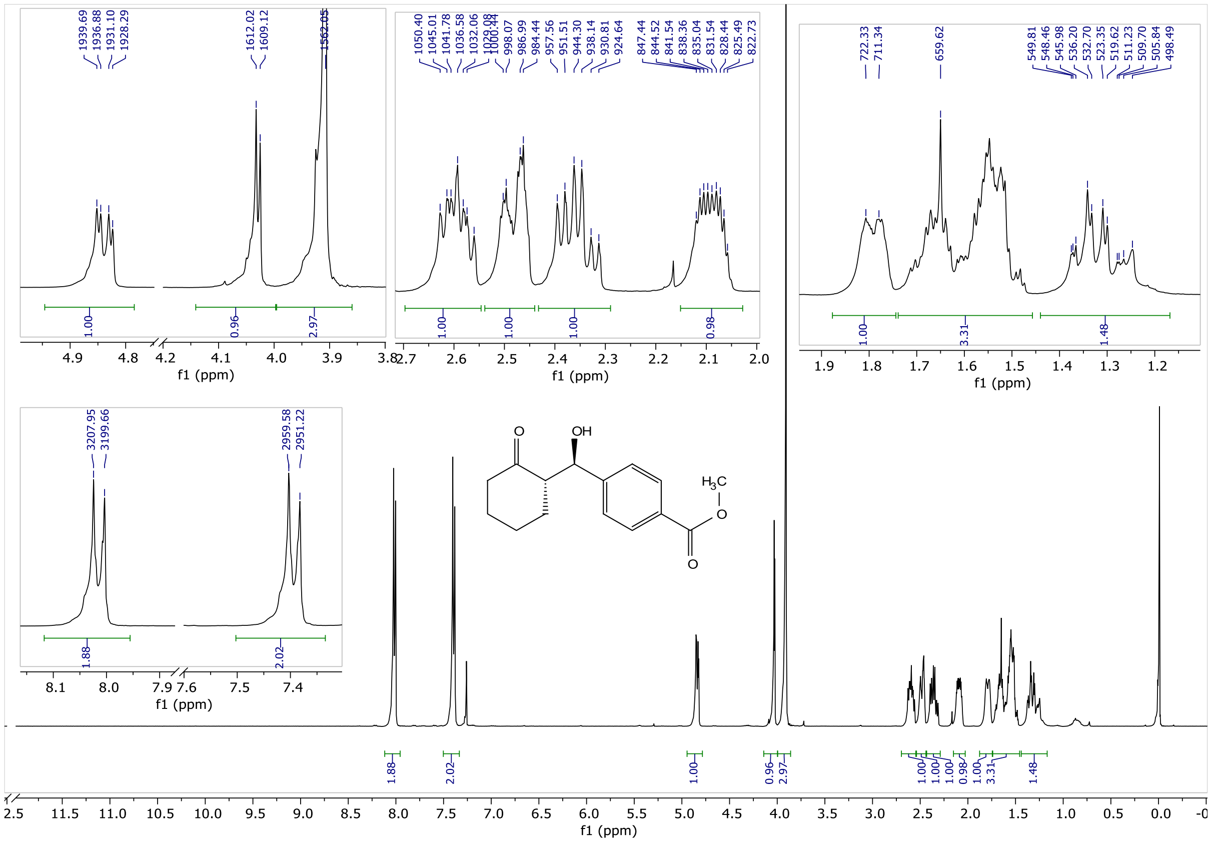

Figure S11. ^{1}H NMR spectrum of the purified *anti*-aldol (major) product **4c** (above).

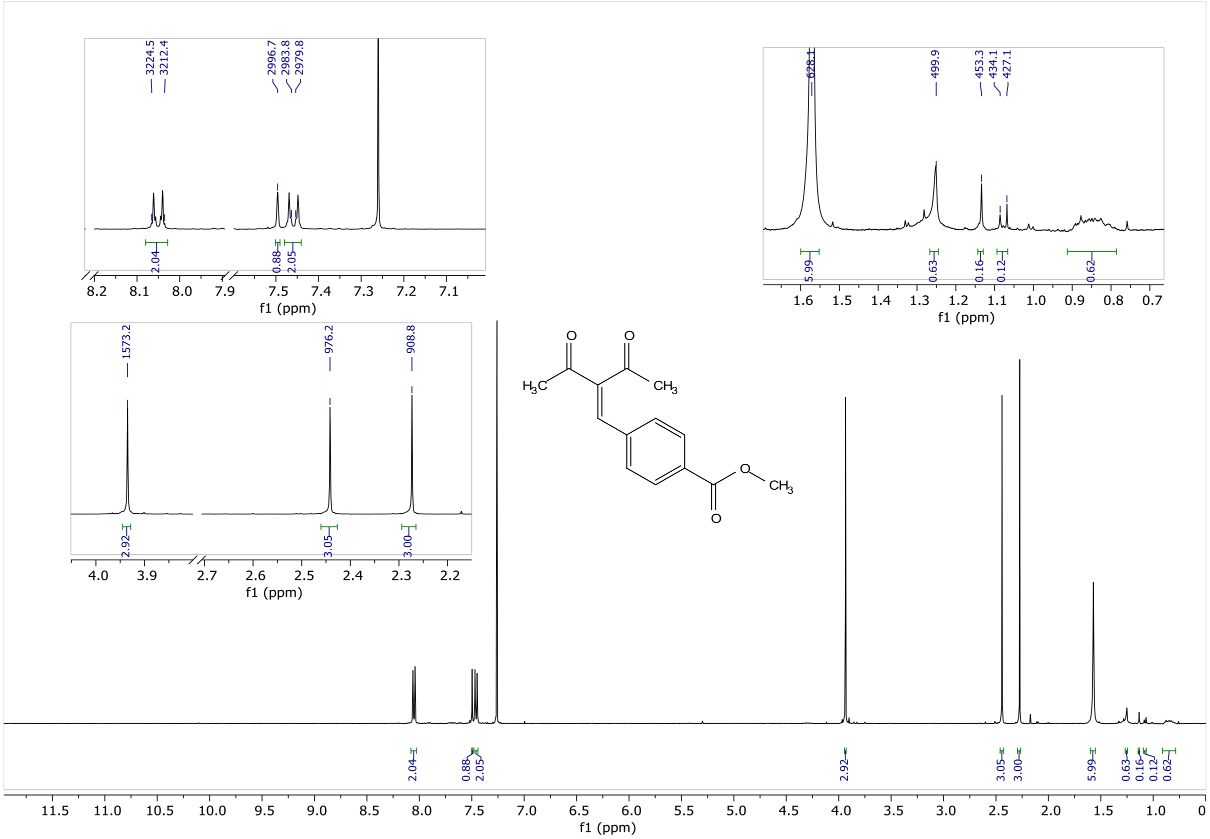

Figure S12. ^{1}H NMR of purified Knoevenagel condensation product (above).

SHIMADZU LabSolutions

Analysis Report

<Sample Information>

Sample Name	: SSZ02 RACE 2mgmL 5%IPAnHex 20uL 1mLmin 17nov22		
Sample ID	:		
Data Filename	: SSZ02 RACE 2mgmL 5%IPAnHex 20uL 1mLmin 17nov22.lcd		
Method Filename	: trial.lcm		
Batch Filename	:		
Vial #	: 1-4	Sample Type	: Unknown
Injection Volume	: 20 uL		
Date Acquired	: 11/17/2022 12:56:08 PM	Acquired by	: System Administrator
Date Processed	: 11/17/2022 1:34:19 PM	Processed by	: System Administrator

<Chromatogram>

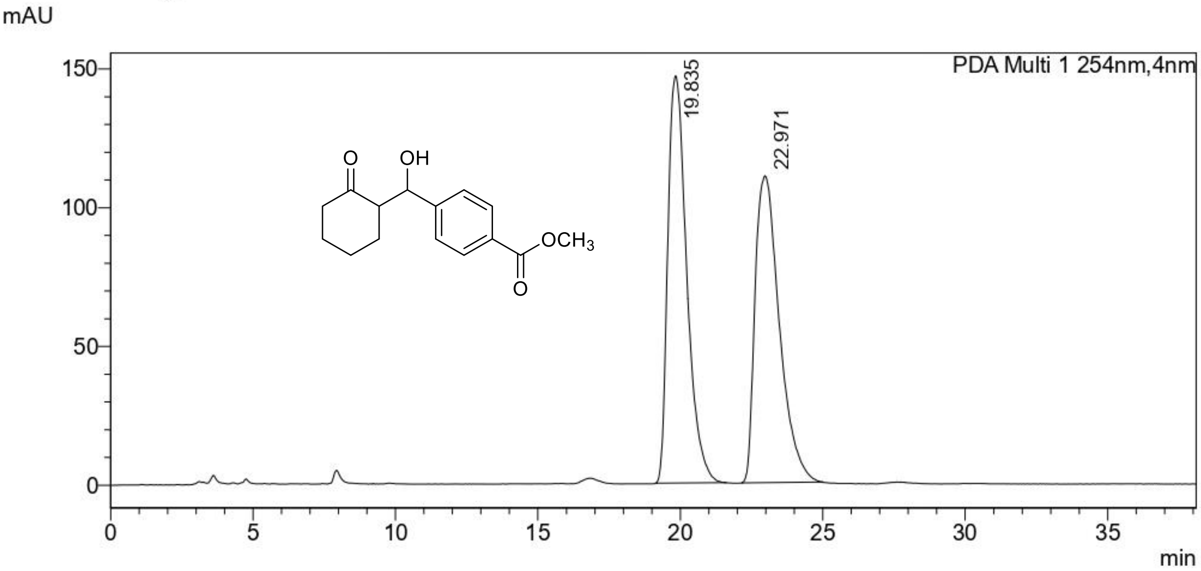

<Peak Table>

PDA Ch1 254nm

Peak#	Ret. Time	Area	Height	Area%
1	19.835	6780885	146718	50.893
2	22.971	6542886	110549	49.107
Total		13323772	257267	100.000

Figure S13. HPLC trace of racemic aldol product **4c** (above).

SHIMADZU LabSolutions Analysis Report

<Sample Information>

Sample Name	: SSZ50 5%IPAnHex 1mLmin 1 mgmL 20 uL 13Nov23		
Sample ID	:		
Data Filename	: SSZ50 5%IPAnHex 1mLmin 1 mgmL 20 uL 13Nov23.lcd		
Method Filename	: Sofiia.lcm		
Batch Filename	:		
Vial #	: 1-1	Sample Type	: Unknown
Injection Volume	: 20 uL		
Date Acquired	: 11/13/2023 1:05:34 PM	Acquired by	: System Administrator
Date Processed	: 11/13/2023 1:35:49 PM	Processed by	: System Administrator

<Chromatogram>

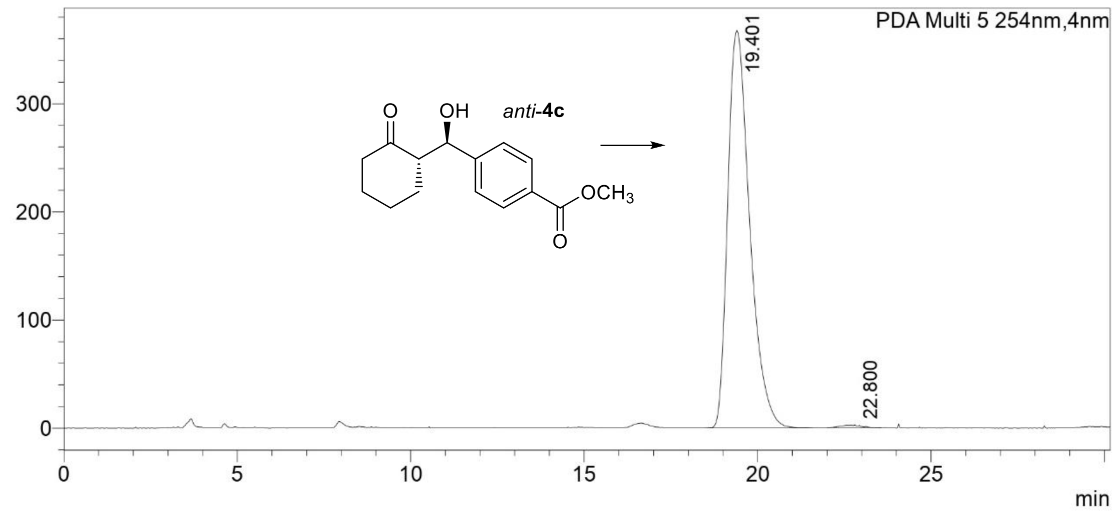

<Peak Table>

PDA Ch5 254nm

Peak#	Ret. Time	Area	Height	Area%
1	19.401	16198587	367562	99.357
2	22.800	104816	2743	0.643
Total		16303402	370305	100.000

Figure S14. HPLC trace of the enantioenriched *anti*-aldol (major) product **4c** (above).

Table 2, entry 4: Competition reaction between cyclohexanone and diethyl malonate for the limiting reactant 4-nitrobenzaldeyhde

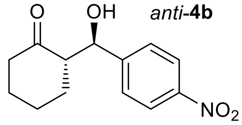

anti-**4b**

(*S*)-2-((*R*)-hydroxy(4-nitrophenyl)methyl)cyclohexanone:

To a clean screw cap V-shaped reaction vessel (5 mL) equipped with a pyramidal stir bar, were added cyclohexanone (MW= 98.14, 1.50 equiv, 2.25 mmol, 220.8 mg, density= 0.947 g/mL, 233 µL), diethyl malonate (MW= 160.17, 1.50 equiv, 2.25 mmol, 360.4 mg, density= 1.055 g/mL, 342 µL), *trans*-4-(tert-butyldiphenylsilyloxy)-L-proline catalyst (MW= 369.54, 2.5 mol%, 0.0375 mmol, 13.9 mg), and mortar and pestle ground 4-nitrobenzaldehyde (MW= 151.12, 1.0 equiv, 1.50 mmol, 227 mg) in the stated order. Within a minute of adding the catalyst, distilled water (MW= 18.02, 15.0 equiv, 22.5 mmol, 405 µL) was added. The resulting heterogenous solution was rigorously stirred for 36 h such that an emulsion was always noted, albeit without causing the contents to splash against the vessel walls, by doing so reproducible yield data was achieved. After several hours the solid reactants fully dissolved.

This compound has been synthesized by many research groups, one example is:
Yang, Y.; Wang, C.; Cheng, Q.; Su, Y.; Li, H.; Xiao, R.; Tan, C.; Liu, G. A Chemo-enzymatic oxidation/aldol sequential process directly converts arylbenzyl alcohols and cyclohexanol into chiral β-hydroxy carbonyls. *Green Chem.* **2021**, *23*, 7773–7779.

Crude product ^{1}H NMR analysis (see below) allowed the following diastereo- and chemoselectivity ratios to be determined.

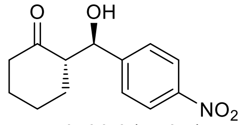

anti-aldol (major)

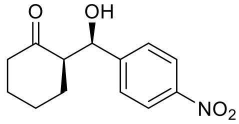

syn-aldol (minor)

Diastereoselectivity: The *anti*/*syn* aldol ratio was determined as >19:1 by comparing the *anti*-aldol product resonance at 4.90 ppm (d, representing the benzylic proton) and the *syn*-aldol product resonance at 5.49 ppm (d, representing the benzylic proton). For the literature regarding the *syn*-aldol product chemical shift, see page 2 of the Supporting Information of the following reference: N. Mase, Y. Nakai, N. Ohara, H. Yoda, K. Takabe, F. Tanaka, C. F. Barbas III, *J. Am. Chem. Soc.* **2006**, *128*, 734–735.

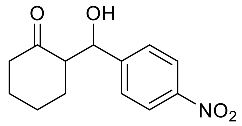

aldol products

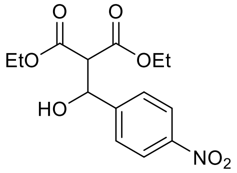

Knoevenagel aldol product

Chemoselectivity: Interestingly the Knoevenagel condensation product was not observed but instead the intermediary Knoevenagel aldol product. The aldol/Knoevenagel aldol product ratio was determined to be 17.3:1, based on the addition of the *anti*-aldol product resonance at 4.90 ppm (d, representing the benzylic proton) and the *syn*-aldol product resonance at 5.49 ppm (d, representing the benzylic proton) *versus* the Knoevenagel aldol product resonance at 3.73 ppm (d, representing the proton alpha to the ester moieties). This Knoevenagel aldol product has been reported, but no ^{1}H NMR data was provided. The ^{1}H NMR of the OtBu diester displays a doublet at 3.51 ppm, see page 4 of the Supporting Information within: A. Massa, A. Scettri, R. Filosa, L. Capozzolo, *Tetrahedron Lett.* **2009**, *50*, 7318-7321).

Purification and yield: Silica gel chromatography (35 mm column outer diameter, 16 cm silica bed height) was performed using gradient elution (20 to 30 vol% ethyl acetate in petroleum ether). The crude product

was loaded onto the column in a minimum volume of CH_2Cl_2. The *anti*- and *syn*-aldol **4b** diastereomers were isolated as a light-yellow solid (310.9 mg, MW= 249.27, 1.25 mmol, 83% yield) of

TLC: R_f=0.27, ethyl acetate/petroleum ether (2:3)

99% *ee*: Chiralpak OD-H, n-hexane/*i*PrOH (93:7), flow rate= 1.0 mL/min, λ = 254 nm, *anti*-aldol product t_{major} = 21.4 min, t_{minor}= 28.2 min (**Figure S18**).

^{1}H NMR (400 MHz, $CDCl_3$) (ppm) *anti*-aldol product **4b**: δ 8.21 (d, 2H, J= 8.8 Hz), 7.50 (d, 2H, J= 8.9 Hz), 4.90 (d, 1H, J= 8.4 Hz), 2.54-2.63 (m, 1H), 2.46-2.53 (m, 1H), 2.31-2.41 (m, 1H), 2.07-2.16 (m, 1H), 1.78-1.87 (m, 1H), 1.48-1.75 (m, 3H), 1.30-1.44 (m, 1H)

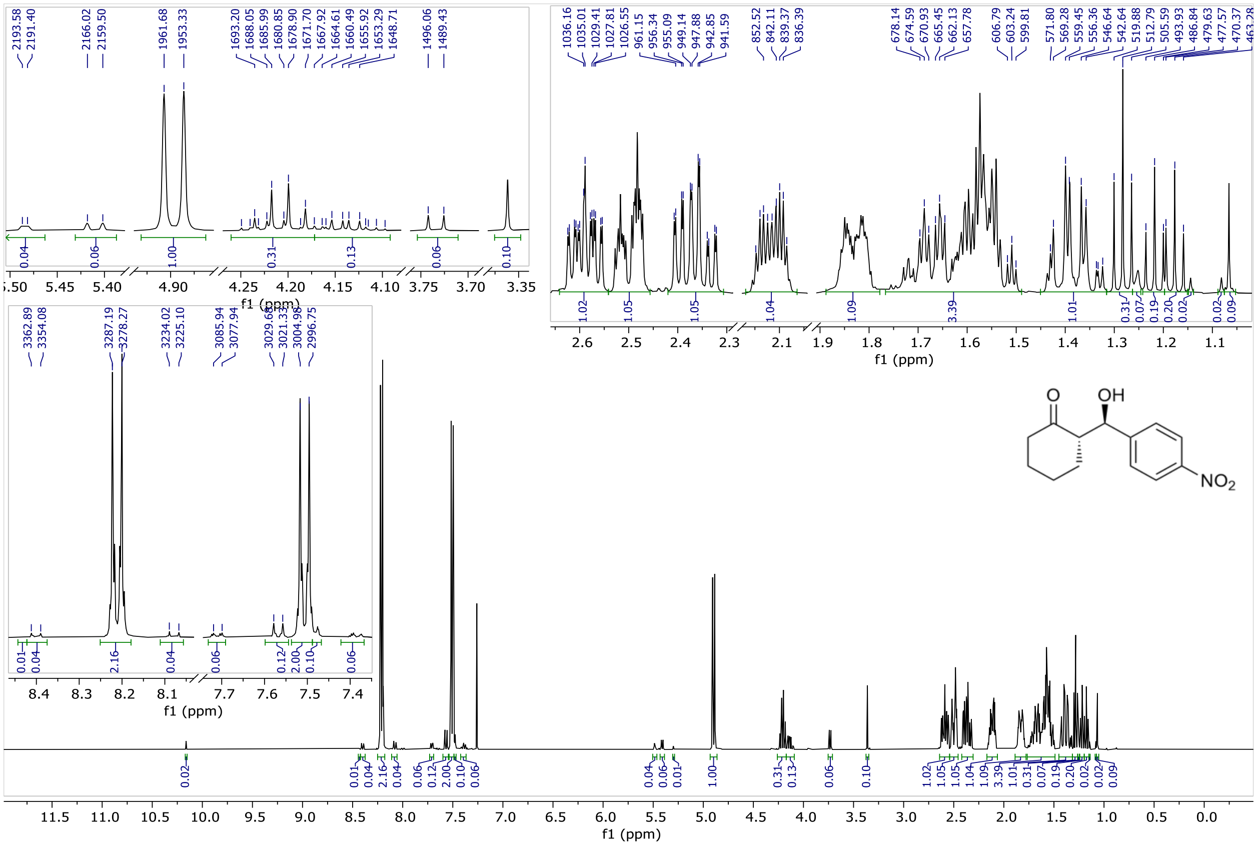

Figure S15. Crude ^{1}H NMR spectrum after high vacuum drying overnight (above).

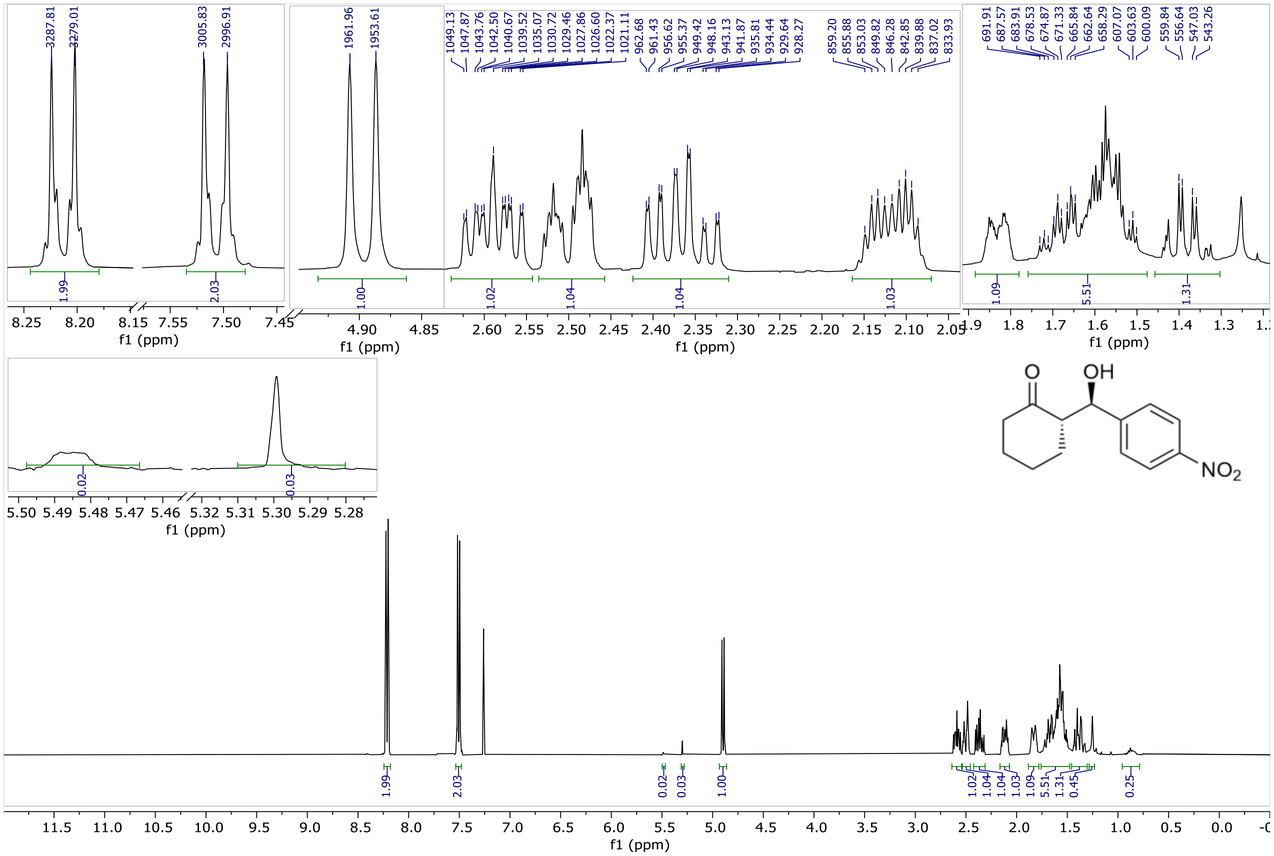

Figure S16. ¹H NMR spectrum of the purified *anti*-(major) and *syn*-(minor) aldol products **4b** (above).

SHIMADZU LabSolutions **Analysis Report**

ADV-A45-7% IPA/nHex 5uL 1mL/min 12May2022

Sample Name	: A46 (RACE)7% IPAnHex 5uL1mLmin 26May 2		
Sample ID	:		
Data Filename	: A46 (RACE)7% IPAnHex 5uL1mLmin 26May 2.lcd		
Method Filename	: trial.lcm		
Batch Filename	:		
Vial #	: 1-1	Sample Type	: Unknown
Injection Volume	: 20 uL		
Date Acquired	: 5/26/2022 2:38:31 PM	Acquired by	: System Administrator
Date Processed	: 5/26/2022 3:28:36 PM	Processed by	: System Administrator

ADV-A46-7% IPA/nHex 5uL 1mL/min 12May2022

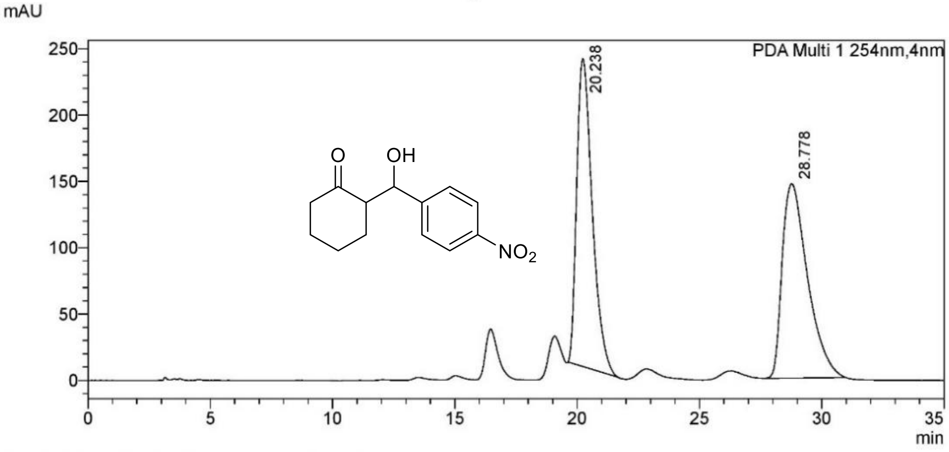

ADV-A45-7% IPA/nHex 5uL 1mL/min 12May2022

PDA Ch1 254nm

Peak#	Ret. Time	Area	Height	Area%
1	20.238	10283216	232276	49.316
2	28.778	10568658	146536	50.684
Total		20851874	378812	100.000

C:\Users\Shimadzu\Desktop\ADV\Competition aldol samples\A46 (RACE)7% IPAnHex 5uL1mLmin 26May 2.lcd

Figure S17. HPLC trace of racemic aldol product **4b** (above).

SHIMADZU LabSolutions **Analysis Report**

<Sample Information>

Sample Name	: HAB-I35 7%IPAnHex 20uL 1mLmin 31oct22		
Sample ID	:		
Data Filename	: HAB-I35 7%IPAnHex 20uL 1mLmin 31oct22.lcd		
Method Filename	: trial.lcm		
Batch Filename	:		
Vial #	: 1-3	Sample Type	: Unknown
Injection Volume	: 20 uL		
Date Acquired	: 10/31/2022 4:08:45 PM	Acquired by	: System Administrator
Date Processed	: 10/31/2022 4:50:19 PM	Processed by	: System Administrator

<Chromatogram>

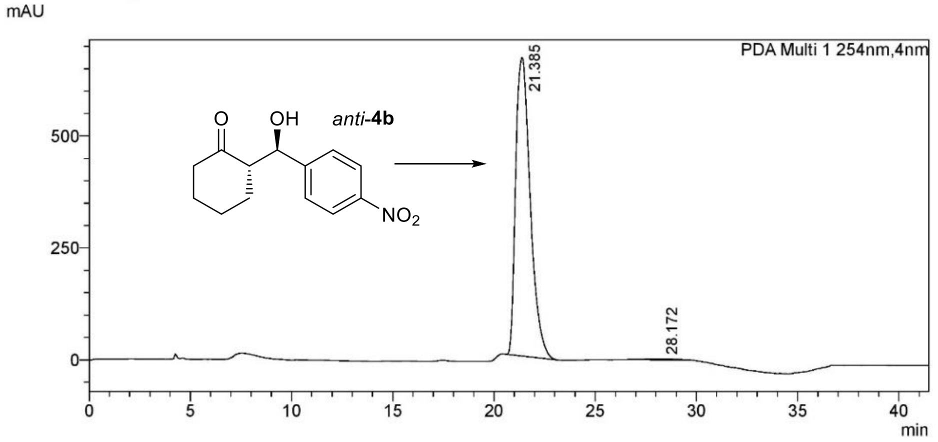

<Peak Table>

PDA Ch1 254nm

Peak#	Ret. Time	Area	Height%	Area%
1	21.385	33389736	99.691	99.291
2	28.172	238503	0.309	0.709
Total		33628239	100.000	100.000

C:\Users\Shimadzu\Desktop\Hanaa\HAB-I35 7%IPAnHex 20uL 1mLmin 31oct22.lcd

Figure S18. HPLC trace of the enantioenriched anti-aldol (major) product **4b** (above).

Table 2, entry 5: Competition reaction between cyclohexanone and methanesulfonylacetone for the limiting reagent 4-(trifluoromethyl)benzaldehyde

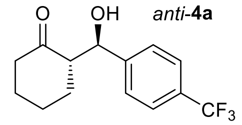

anti-**4a**

(*S*)-2-((*R*)-hydroxy(4-(trifluoromethyl)phenyl)methyl)cyclohexan-1-one

To a clean screw cap V-shaped reaction vessel (5.0 mL) equipped with a pyramidal stir bar, were subsequently added cyclohexanone (MW = 98.14, 1.50 equiv, 2.25 mmol, density = 0.947 g/mL, 233 µL), methanesulfonylacetone (MW= 136.17, 1.50 equiv, 2.25 mmol, 306 mg), *trans*-4-(tert-butyldiphenylsilyloxy)-L-proline catalyst (MW = 369.54, 5.0 mol%, 0.075 mmol, 27.7 mg) and purified 4-(trifluoromethyl)benzaldehyde (MW = 174.12, 1.00 equiv, 1.50 mmol, density = 1.275 g/mL, 205 µL) in the stated order. Within a minute of adding the catalyst, water (MW = 18.02, 15.0 equiv, 22.5 mmol, 405 µL) was added. The resulting heterogenous solution was milky white in appearance and readily stirred (fully fluid) for the 20 h reaction time. The reaction solution was efficiently stirred albeit without causing the contents to splash against the vessel walls, by doing so reproducible yield data was achieved.

This compound has been synthesized by many research groups, one example is:
Miura, T.; Imai, K.; Ina, M.; Tada, N.; Imai, N.; Itoh, A. Direct asymmetric aldol reaction with recyclable fluorous organocatalyst. *Org. Lett.* **2010**, *12*, 1620–1623.

Crude product ^{1}H NMR analysis (**Figure S19** below) allowed the following diastereo- and chemoselectivity ratios to be determined. Note: For the crude ^{1}H NMR data, taking a random small portion of the crude semi-solid reaction product of this reaction did not result in reproducible data, thus the entire crude reaction product was dissolved in $CDCl_3$ and then the ^{1}H NMR data was collected. That is, in our hand, when examining a random sample of these crude reaction products, a solid/simultaneously viscous oil, a different chemoselectivity was noted each time.

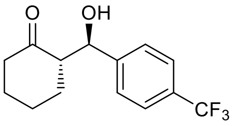

anti-aldol (major)

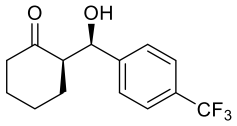

syn-aldol (minor)

Diastereoselectivity: The *anti*-aldol/*syn*-aldol product ratio was determined as >19:1, based on the *anti*-aldol product resonance at 4.84 ppm (the doublet of doublets representing the benzylic proton) and the *syn*-aldol product resonance at 5.45 ppm (doublet representing the benzylic proton). For literature regarding the *syn*-aldol product chemical shift, see page 7, product **6d**, within the Supporting Information of: Y. Wu, Y. Zhang, M. Yu, G. Zhao, S. Wang, *Org. Lett.* **2006**, *8*, 4417–4420.

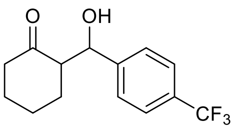

aldol products

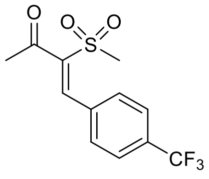

Knoevenagel product

Chemoselectivity: The aldol/Knoevenagel product ratio was determined to be >19:1. In this instance, no Knoevenagel aldol or Knoevenagel condensation product was observed by crude ^{1}H NMR analysis. No literature exists for the Knoevenagel aldol or condensation product.

Purification and yield: Silica gel chromatography (35 mm column outer diameter, 16 cm silica bed height) was performed using gradient elution (1.5 to 2 vol% acetone in CH_2Cl_2) to remove the starting material traces. The crude product was always loaded onto the column in a minimum volume of CH_2Cl_2. The *anti*-aldol product **4a** was isolated as a single diastereomer as a white solid (250.9 mg, MW = 272.27, 0.92

mmol, 61% yield). Under the same reaction conditions but for a 36 h reaction time the obtained yield was the same (61%).

TLC: R_f = 0.20 acetone/CH_2Cl_2 (1:66).

99% *ee*: Chiralpak OD-H chiral HPLC column, n-hexane/iPrOH (80:20), flow rate = 0.5 mL/min, λ = 216 nm, *anti*-aldol product t_{major}= 11.2 min, *syn*-aldol product t_{minor}= 13.2 min retention times were observed (**Figure S22**).

^{1}H NMR (400 MHz, $CDCl_3$) (ppm) *anti*-aldol product **4a**: δ 7.61 (d, 1H, J= 8.2 Hz), 7.45 (d, 1H, J= 8.1 Hz), 4.84 (dd, 1H, J = 8.6, 2.9 Hz), 4.03 (d, 1H, J = 3.0 Hz), 2.64–2.55 (m, 1H), 2.54 – 2.45 (dm, 1H, J = 13.6 Hz), 2.42 – 2.31 (dt, 1H, J = 13.2, 6.0 Hz), 2.16 – 2.01 (m, 1H), 1.87 – 1.76 (dm, 1H, J = 13.8 Hz), 1.74 – 1.62 (m, 1H), 1.62–1.50 (m, 4H), 1.39–1.26 (m, 1H).

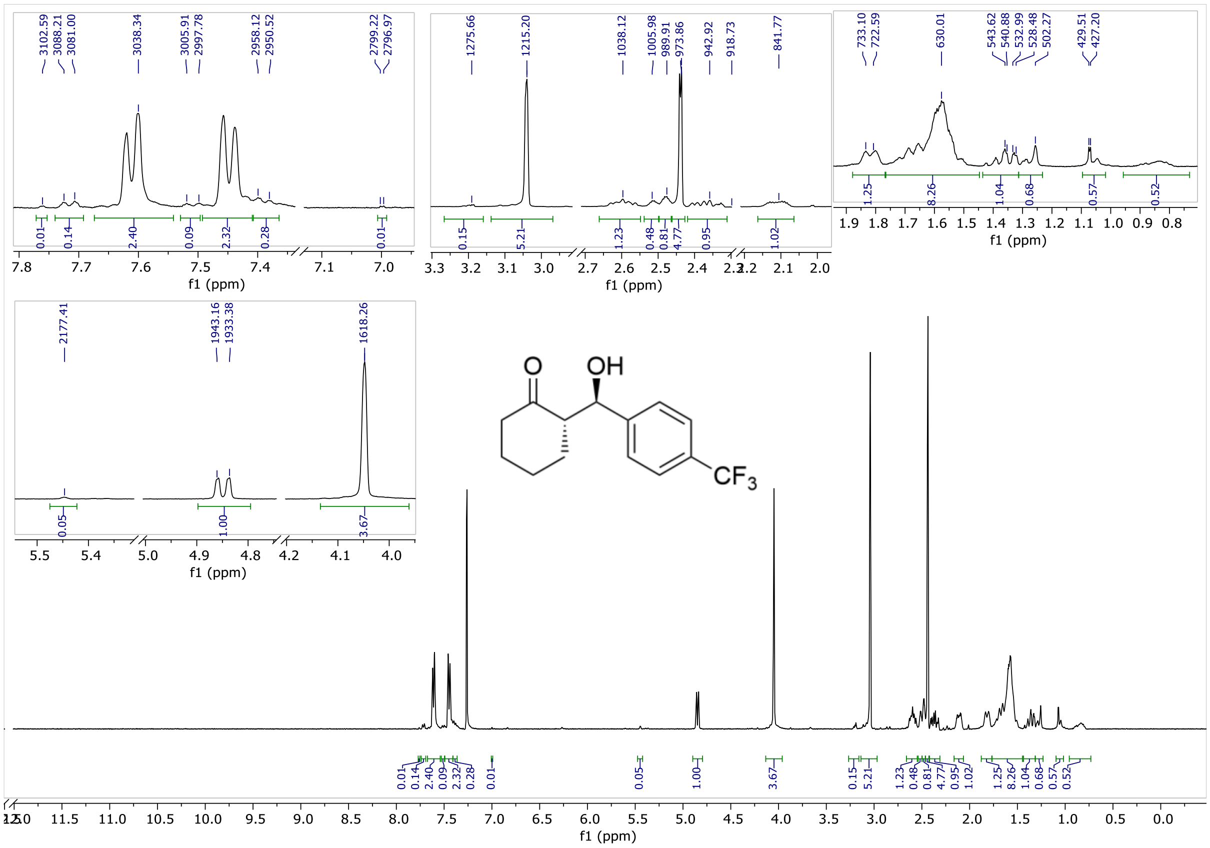

Figure S19. Crude ^{1}H NMR spectrum after high vacuum drying overnight (above).

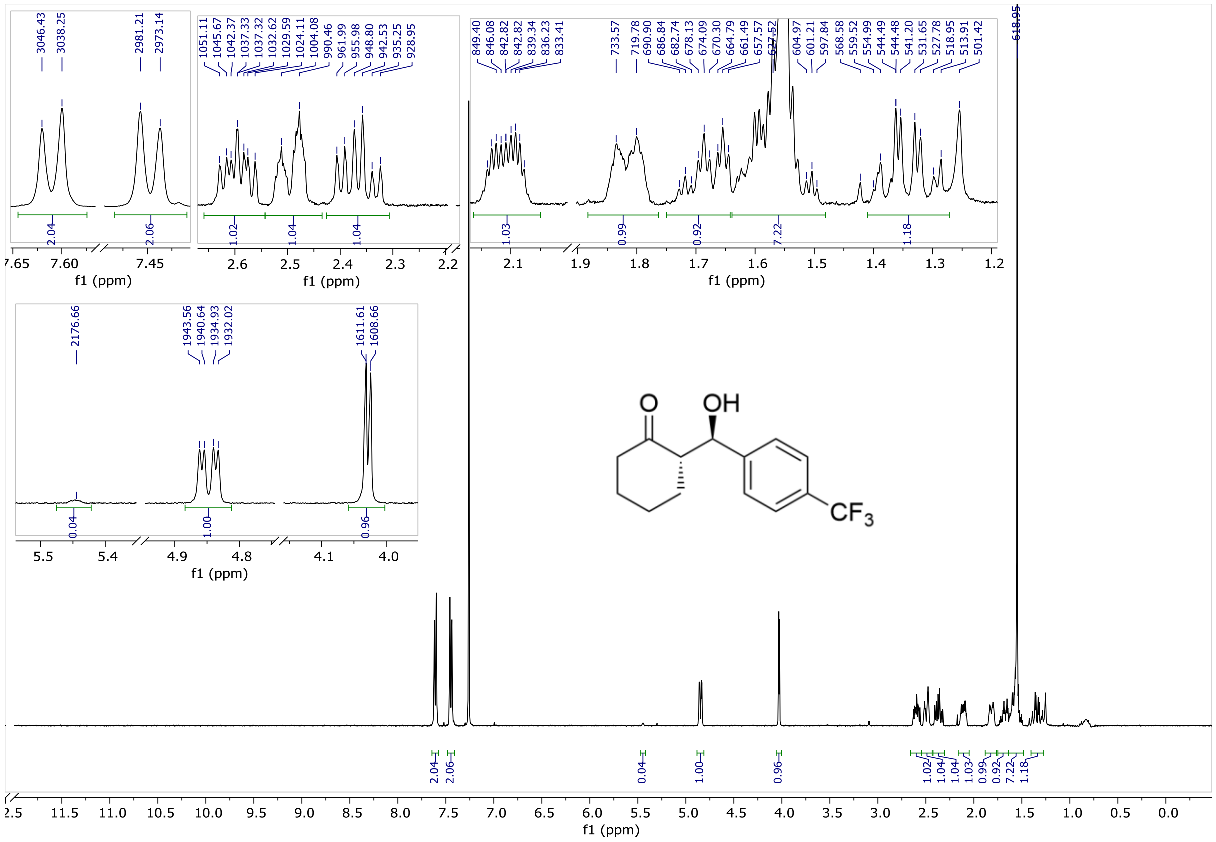

Figure S20. ^{1}H NMR spectrum of the purified *anti*(major)- and *syn*(minor)-aldol products **4a** (above).

SHIMADZU LabSolutions

Analysis Report

<Sample Information>

Sample Name	: HC-I-26 20%IPAnHex 20uL 0.5 mLmin 08Feb23 1mgmL		
Sample ID	:		
Data Filename	: HC-I-26 20%IPAnHex 20uL 0.5 mLmin 08Feb23 1mgmL.lcd		
Method Filename	: trial.lcm		
Batch Filename	:		
Vial #	: 1-4	Sample Type	: Unknown
Injection Volume	: 20 uL		
Date Acquired	: 2/8/2023 3:15:20 PM	Acquired by	: System Administrator
Date Processed	: 2/8/2023 3:39:35 PM	Processed by	: System Administrator

<Chromatogram>

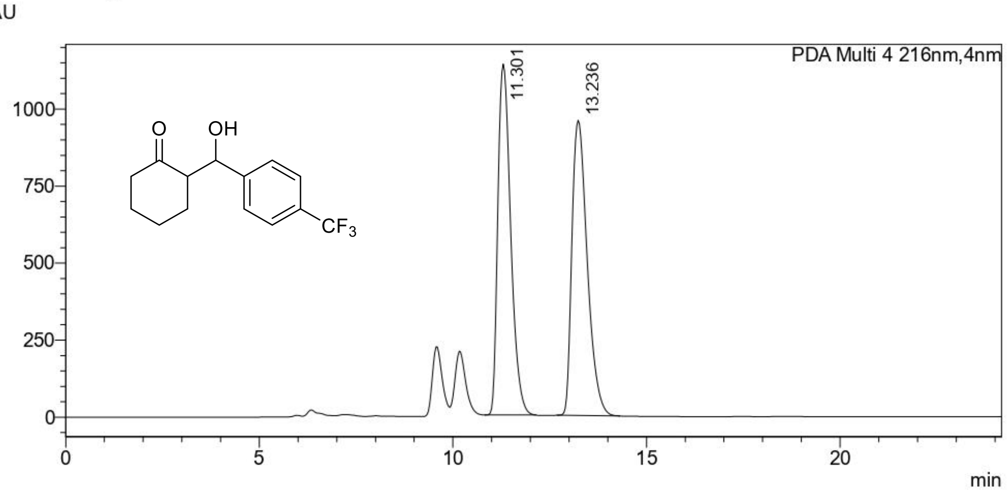

<Peak Table>

PDA Ch4 216nm

Peak#	Ret. Time	Area	Height	Area%
1	11.301	26277677	1138787	49.940
2	13.236	26341028	958736	50.060
Total		52618705	2097523	100.000

Figure S21. HPLC trace of racemic aldol product **4a** (above).

SHIMADZU LabSolutions

Analysis Report

<Sample Information>

Sample Name	: SS21-trial3 20%IPAnHex 10uL 0.5 mLmin 21Feb23 1mgmL
Sample ID	:
Data Filename	: SS21-trial3 20%IPAnHex 10uL 0.5 mLmin 21Feb23 1mgmL.lcd
Method Filename	: trial.lcm
Batch Filename	:
Vial #	: 1-3
Sample Type	: Unknown
Injection Volume	: 10 uL
Date Acquired	: 2/21/2023 12:44:14 PM
Acquired by	: System Administrator
Date Processed	: 2/21/2023 1:04:50 PM
Processed by	: System Administrator

<Chromatogram>

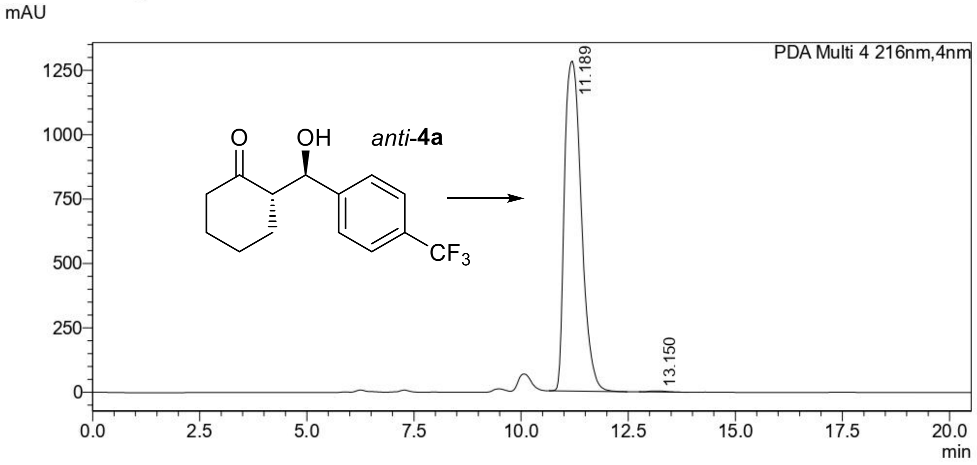

<Peak Table>

PDA Ch4 216nm

Peak#	Ret. Time	Area	Height	Area%
1	11.189	35146060	1281437	99.685
2	13.150	111209	3980	0.315
Total		35257269	1285417	100.000

Figure S22. HPLC trace of the enantioenriched *anti*-aldol (major) product **4a**.

Table 2, entry 6: Competition reaction between cyclohexanone and ethyl-2-phenylacetate for the limiting reactant methyl 4-formylbenzoate

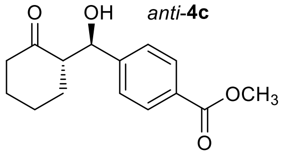

Methyl 4-((*R*)-hydroxy((*S*)-2-oxocyclohexyl)methyl)benzoate

To a clean screw cap V-shaped reaction vessel (5.0 mL) equipped with a pyramidal stir bar, were added cyclohexanone (MW= 98.14, 1.50 equiv, 2.25 mmol, 221 mg, density= 0.947 g/mL, 233 µL), ethyl-2- phenylacetate (MW= 164.20, 1.50 equiv, 2.25 mmol, 369 mg, density= 1.03 g/mL, 359 µL), mortar and pestle ground methyl 4-formylbenzoate (MW= 164.16, 1.00 equiv, 1.50 mmol, 246 mg), and *trans*-4-(*tert*-butyldiphenylsilyloxy)-L-proline catalyst (MW= 369.54, 5.0 mol%, 0.075 mmol, 27.7 mg) in the stated order. Within a minute of adding the catalyst, distilled water (MW= 18.02, 15.0 equiv, 22.5 mmol, 405.5 µL) was added. After several minutes the solid reactants fully dissolved, but an emulsion was noted for the entirety of the reaction. The resulting heterogenous solution was rigorously stirred for 24 h albeit without causing splashing against the vessel walls, by doing so reproducible yield data was achieved.

This compound has been synthesized by many research groups, one example is:
Inoue, H.; Kikuchi, M.; Ito, J.-i.; Nishiyama, H. Chiral phebox-rhodium complexes as catalysts for asymmetric direct aldol reaction. Tetrahedron 2008, 64, 493-499.

Crude product ^{1}H NMR analysis (Figure **S23**) allowed the following diastereo- and chemoselectivity ratios to be determined.

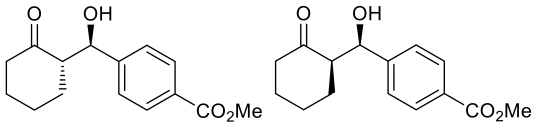

anti-aldol (major) *syn*-aldo (minor)

Diastereoselectivity: The *anti*/*syn* aldol ratio was determined to be 16.7:1 by comparing the *anti*-aldol product resonance at 4.84 ppm (d, representing the benzylic proton) and the *syn*-aldol product resonance at 5.44 ppm (bs, representing the benzylic proton). For literature regarding the *syn*-aldol product chemical shift, see page 2 of the Supporting Information within: N. Mase, Y. Nakai, N. Ohara, H. Yoda, K. Takabe, F. Tanaka, C. F. Barbas III, *J. Am. Chem. Soc.* **2006**, *128*, 734–735.

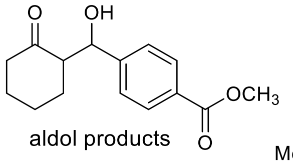

aldol products

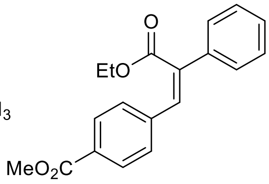

Knoevenagel product

Chemoselectivity: The aldol/Knoevenagel product ratio was determined to be >19:1. In this instance, no Knoevenagel aldol or Knoevenagel condensation product was observed by crude ^{1}H NMR analysis. The Knoevenagel aldol and Knoevenagel condensation products of ethyl phenylacetate and methyl 4-formylbenzoate have not been previously reported. However, the Knoevenagel condensation product of methyl phenylacetate and methyl 4-formylbenzoate has been reported. See compound **7l** on page 5201, within: F.-X. Felpin, K. Miqueu, J.-M. Sotiropoulos, E. Fouquet, O. Ibarguren, J. Laudien, *Chem. Eur. J.* **2010**, *16*, 5191–5204. For that compound, the olefinic proton is located at 7.06 ppm (Z isomer) and at 7.86 ppm (E isomer). Analysis of our crude ^{1}H NMR shows no obvious singlet of significance appearing at ± 0.15 ppm from either 7.06 or 7.86 ppm.

Purification and yield: Silica gel chromatography (35 mm column outer diameter, 16 cm silica bed height) was performed using gradient elution (15 to 30 vol% ethyl acetate in petroleum ether). The crude product

was loaded onto the column in a minimum volume of dichloromethane. The *anti*-aldol **4c** product was isolated as a single diastereomer as a light-yellow solid (328.4 mg, MW= 262.31, 1.25 mmol, 83% yield).

TLC: R_f=0.42, ethyl acetate/petroleum ether (2:3)

99% *ee*: Chiralpak OD-H, n-hexane/*i*PrOH (95:5), flow rate= 1.0 mL/min, λ = 254 nm, *anti*-aldol product t_{major} = 19.5 min, t_{minor}= 22.5 min (**Figure S26**).

^{1}H NMR (400 MHz, $CDCl_3$) (ppm) *anti*-aldol product **4c**: δ 8.02 (d, 2H, J= 8.1 Hz), 7.40 (d, 2H, J= 8.6 Hz), 4.84 (dd, 1H, J= 8.6 Hz and 3.1 Hz), 4.02 (d, 1H, *OH,* J= 3.0 Hz), 3.91 (s, 3H), 2.55-2.65 (m, 1H), 2.44-2.53 (m, 1H), 2.30-2.42 (m, 1H), 2.05-2.15 (m, 1H), 1.75-1.84 (m, 1H), 1.60-1.73 (m, 1H) 1.47-1.60 (m, 2H), 1.30-1.39 (m, 1H)

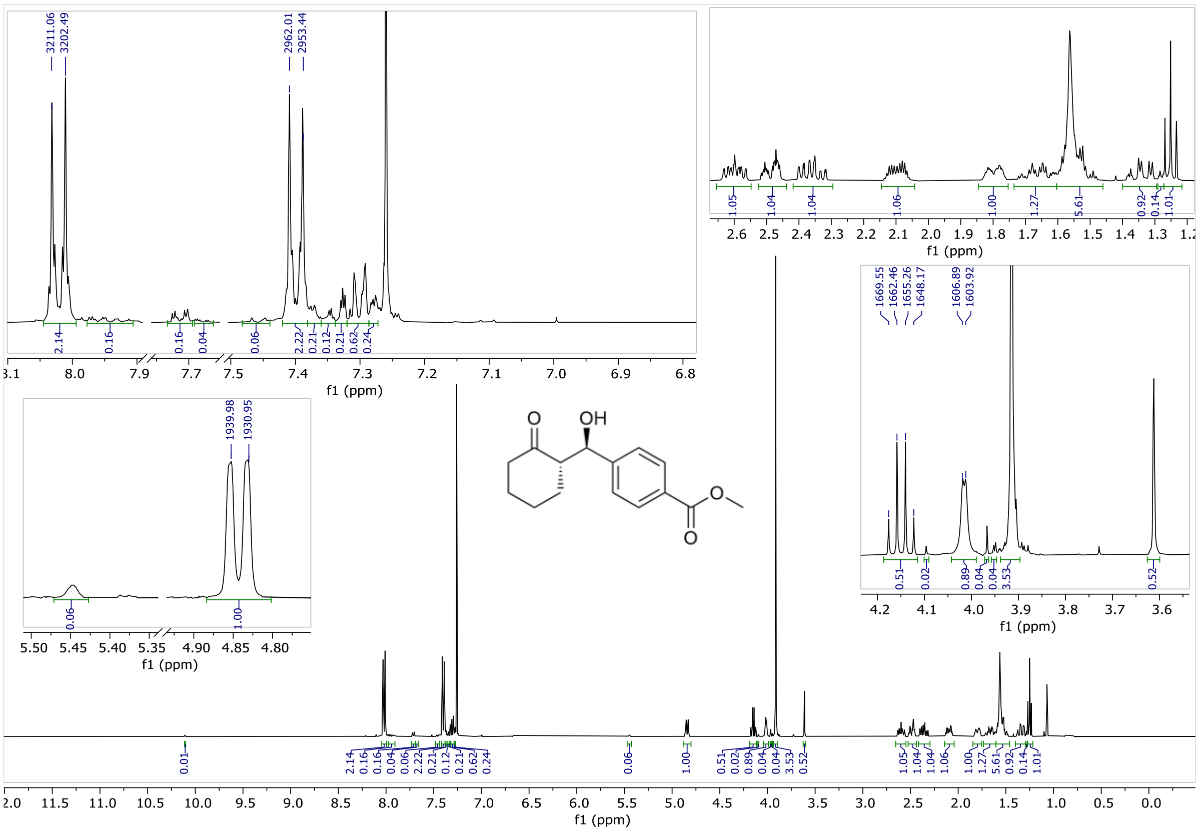

Figure S23. Crude ^{1}H NMR spectrum after high vacuum drying overnight (above).

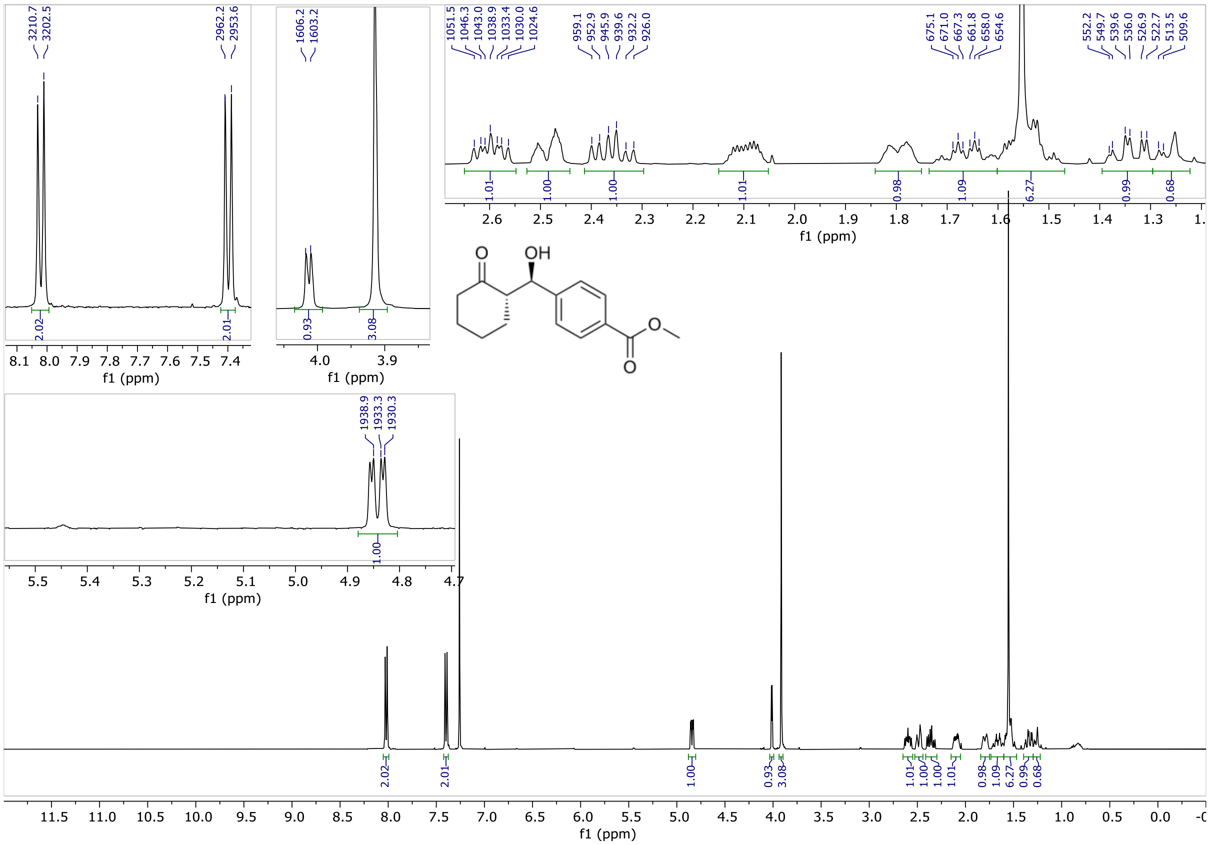

Figure S24. ^{1}H NMR spectrum of the purified *anti*-aldol (major) product **4c** (above).

SHIMADZU LabSolutions

Analysis Report

<Sample Information>

Sample Name	: SSZ02 RACE 5%IPAnHex 20uL 1mLmin 02nov22		
Sample ID	:		
Data Filename	: SSZ02 RACE 5%IPAnHex 20uL 1mLmin 02nov22.lcd		
Method Filename	: trial.lcm		
Batch Filename	:		
Vial #	: 1-4	Sample Type	: Unknown
Injection Volume	: 20 uL		
Date Acquired	: 11/2/2022 6:45:55 PM	Acquired by	: System Administrator
Date Processed	: 11/2/2022 7:17:43 PM	Processed by	: System Administrator

<Chromatogram>

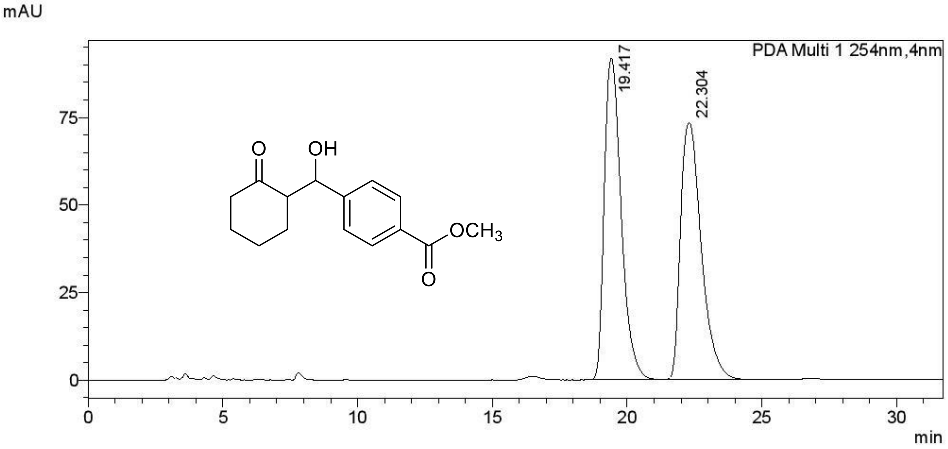

<Peak Table>

PDA Ch1 254nm

Peak#	Ret. Time	Area	Height	Area%
1	19.417	3982137	91586	50.486
2	22.304	3905444	73306	49.514
Total		7887580	164893	100.000

C:\Users\Shimadzu\Desktop\SZ\HPLC\Runs\SSZ02 RACE 5%IPAnHex 20uL 1mLmin 02nov22.lcd

Figure S25. HPLC trace of racemic aldol product **4c** (above).

SHIMADZU LabSolutions

Analysis Report

<Sample Information>

Sample Name	: HAB-I54 5%IPAnHex 120uL 1 mLmin 06Mar23		
Sample ID	:		
Data Filename	: HAB-I54 5%IPAnHex 120uL 1 mLmin 06Mar23.lcd		
Method Filename	: trial.lcm		
Batch Filename	:		
Vial #	: 1-3	Sample Type	: Unknown
Injection Volume	: 20 uL		
Date Acquired	: 3/6/2023 2:14:33 PM	Acquired by	: System Administrator
Date Processed	: 3/6/2023 2:45:48 PM	Processed by	: System Administrator

<Chromatogram>

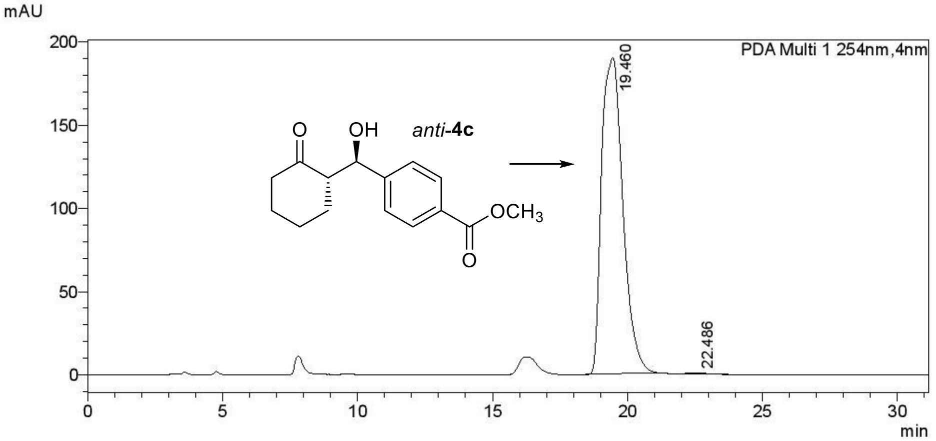

<Peak Table>

PDA Ch1 254nm

Peak#	Ret. Time	Area	Height	Area%
1	19.460	10400695	190217	99.673
2	22.486	34110	638	0.327
Total		10434804	190854	100.000

C:\Users\Shimadzu\Desktop\Hanaa\HAB-I54 5%IPAnHex 120uL 1 mLmin 06Mar23.lcd

Figure S26. HPLC trace of enantioenriched *anti*-aldol (major) product **4c** (above).

Table 2, entry 7: Competition reaction between cyclohexanone and chloroacetone for the limiting reactant methyl 4-formylbenzoate

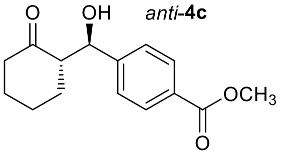
anti-**4c**

Methyl 4-((*R*)-hydroxy((*S*)-2-oxocyclohexyl)methyl)benzoate

To a clean screw cap V-shaped reaction vessel (5.0 mL) equipped with a pyramidal stir bar, were added cyclohexanone (MW= 98.14, 1.50 equiv, 2.25 mmol, 221 mg, density= 0.947 g/mL, 233 µL), chloroacetone (MW= 92.52, 1.50 equiv, 2.25 mmol, 208 mg, density= 1.162 g/mL, 179 µL), mortar and pestle ground methyl 4-formylbenzoate (MW= 164.16, 1.0 equiv, 1.5 mmol, 246.2 mg), and *trans*-4-(*tert*-butyldiphenylsilyloxy)-L-proline catalyst (MW= 369.54, 2.5 mol%, 0.0375 mmol, 13.9 mg) in the stated order. Within a minute of adding the catalyst, distilled water (MW= 18.02, 15 equiv, 22.5 mmol, 405 µL) was added. Chloroacetone is light-sensitive so the reaction vessel was always wrapped in aluminum foil to exclude light. After several minutes the solid reactants fully dissolved. The resulting heterogenous solution was rigorously stirred for 30 h such that an emulsion was always noted, albeit without causing content splashing against the vessel walls, by doing so reproducible yield data was achieved.

This compound has been synthesized by many research groups, one example is:
Inoue, H.; Kikuchi, M.; Ito, J.-i.; Nishiyama, H. Chiral phebox-rhodium complexes as catalysts for asymmetric direct aldol reaction. *Tetrahedron* **2008**, *64*, 493-499.

Crude product ^{1}H NMR analysis (**Figure S27**) allowed the following diastereo- and chemoselectivity ratios to be determined.

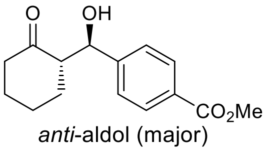
anti-aldol (major)

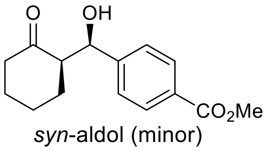
syn-aldol (minor)

Diastereoselectivity: The *anti*/*syn* aldol ratio was determined as 16.7:1 by comparing the *anti*-aldol product resonance at 4.84 ppm (the doublet representing the benzylic proton) and the *syn*-aldol product resonance at 5.44 ppm (bs, representing the benzylic proton). For literature regarding the *syn*-aldol product chemical shift, see page 2 of the Supporting Information within: N. Mase, Y. Nakai, N. Ohara, H. Yoda, K. Takabe, F. Tanaka, C. F. Barbas III, *J. Am. Chem. Soc.* **2006**, *128*, 734–735.

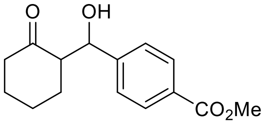
aldol product

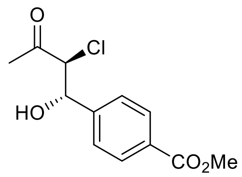
Knoevenagel
anti-aldol product

Chemoselectivity: The term regioselectivity is more appropriate for this aldol *versus* aldol competition reaction. Despite this the chloroacetone product is labeled as the Knoevenagel product, and only the Knoevenagel *anti*-aldol product was observed. The aldol/Knoevenagel *anti*-aldol product ratio was determined to be 15.1:1 based on the addition of the *anti*-aldol product resonance at 4.84 ppm (d, representing the benzylic proton) and the *syn*-aldol product resonance at 5.44 ppm (d, representing the benzylic proton) *versus* the Knoevenagel *anti*-aldol product resonance at 5.09 ppm (d, representing the benzylic proton). For literature regarding the chloroacetone *anti*-aldol product chemical shifts, see page 350 within: L. He, Z. Tang, L. Cun, A. Mi, Y. Jiang, L. Gong, *Tetrahedron* **2006**, *62*, 346–351. The absolute stereochemistry of the shown Knoevenagel *anti*-aldol product is assumed based on reports that L-proline templated catalysts form it.

The *syn*-aldol product of chloroacetone and methyl 4-formylbenzoate has a doublet at 5.33 representing the benzylic proton, but it is not observed in our crude ^{1}H NMR (**Figure S27**). For the literature regarding the Knoevenagel *syn*-aldol product chemical shifts, see page S-7 of the Supporting Information within: A. Umehara, T. Kanemitsu, K. Nagata, T. Itoh, *Synlett* **2012**, *23*, 453-457.

The Knoevenagel condensation product for chloroacetone and methyl 4-formylbenzoate is not reported in the literature. The methyl group of the Z- and E-Knoevenagel condensation products of chloroacetone with benzaldehyde respectively appear at 2.50 and 2.27 ppm and the alkene proton is found at either 7.72 ppm (Z-isomer) or 7.17 (E-isomer), see page 2643 within: K.-M. Kim, I.-H. Park, *Synthesis* **2004**, *16*, 2641–2644. Examination of our crude ^{1}H NMR spectrum (**Figure S27**) did not reveal similar chemical shifts for either the E- or Z-Knoevenagel condensation products.

An unknown product shows a doublet at 5.03 ppm within the crude ^{1}H NMR spectrum (**Figure S27**) and based on the above analysis it is not the Knoevenagel: (i) *anti*-aldol, (ii) *syn*-aldol, or (iii) E- or Z-condensation product.

Purification and yield: Silica gel chromatography (35 mm column outer diameter, 16 cm silica bed height) was performed using gradient elution (20 to 30 vol% ethyl acetate in petroleum ether). The crude product was loaded onto the column in a minimum volume of dichloromethane. The *anti*-aldol product **4c** was isolated as a single diastereomer as a light-yellow solid (323.1 mg, MW= 262.31, 1.23 mmol, 82% yield).

TLC: R_f=0.42, ethyl acetate/petroleum ether (2:3)

98% *ee*: Chiralpak OD-H, n-hexane/*i*PrOH (95:5), flow rate= 1.0 mL/min, λ = 254 nm, *anti*-aldol product t_{major} = 18.8 min, t_{minor}= 21.6 min (**Figure S30**).

^{1}H NMR (400 MHz, $CDCl_3$) (ppm) anti-aldol product **4c**: δ 8.02 (d, 2H, J= 8.5 Hz), 7.40 (d, 2H, J= 8.1 Hz), 4.84 (dd, 1H, J= 2.8, 8.7 Hz), 4.02 (d, 1H, *OH,* J= 2.9 Hz), 3.91 (s, 3H), 2.55-2.65 (m, 1H), 2.44-2.53 (m, 1H), 2.30-2.42 (m, 1H), 2.05-2.15 (m, 1H), 1.75-1.84 (m, 1H), 1.60-1.73 (m, 1H) 1.47-1.60 (m, 2H), 1.30-1.39 (m, 1H)

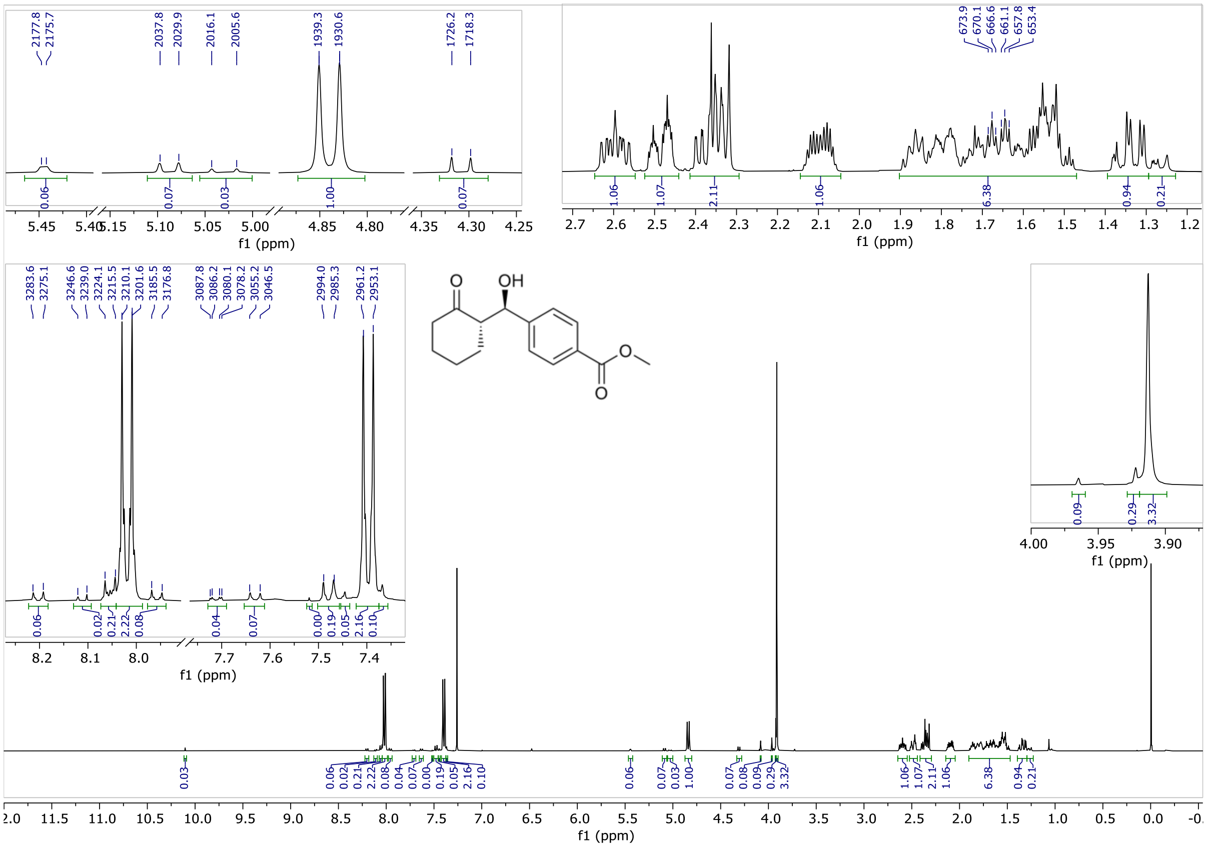

Figure S27. Crude ^{1}H NMR spectrum after high vacuum drying overnight (above).

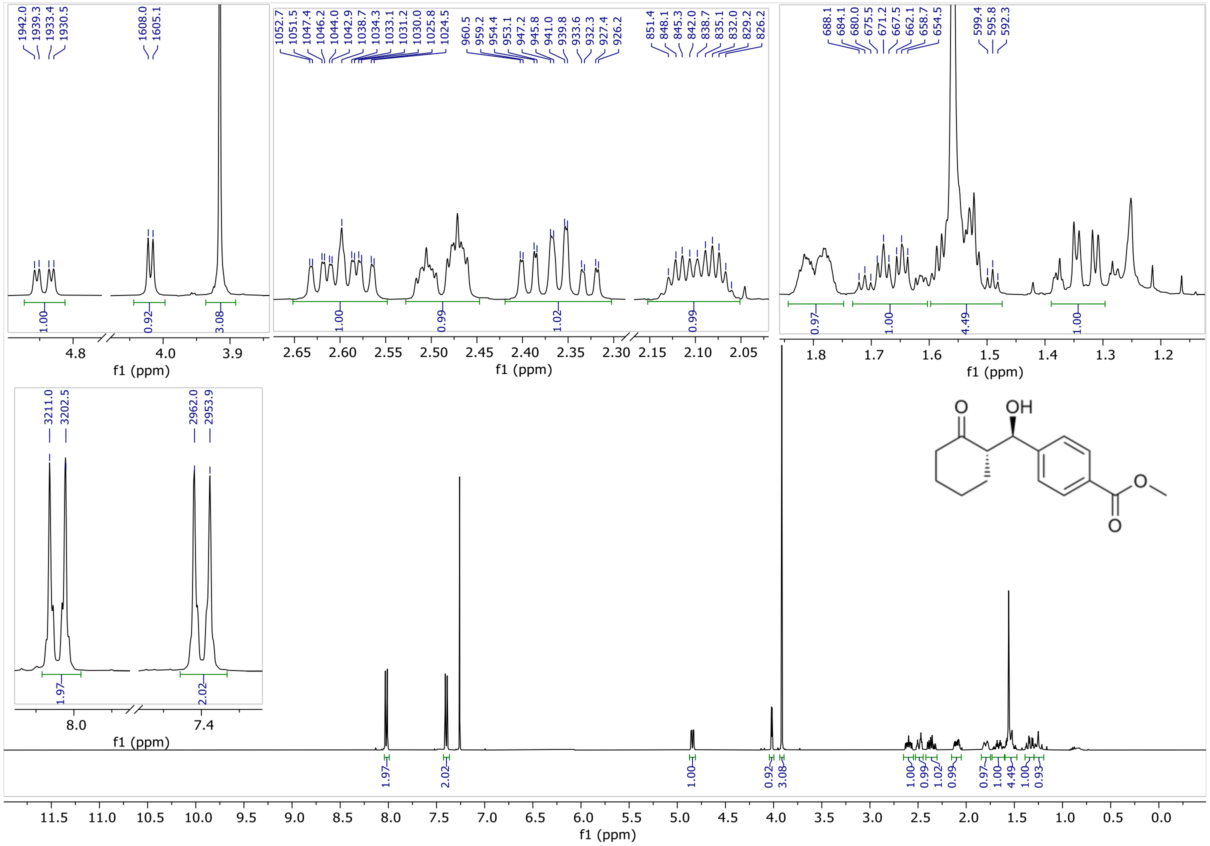

Figure S28. ^{1}H NMR spectrum of the purified *anti*-aldol (major) product **4c** (above).

SHIMADZU LabSolutions

Analysis Report

<Sample Information>

Sample Name	: SSZ02 RACE 5%IPAnHex 20uL 1mLmin 02nov22		
Sample ID	:		
Data Filename	: SSZ02 RACE 5%IPAnHex 20uL 1mLmin 02nov22.lcd		
Method Filename	: trial.lcm		
Batch Filename	:		
Vial #	: 1-4	Sample Type	: Unknown
Injection Volume	: 20 uL		
Date Acquired	: 11/2/2022 6:45:55 PM	Acquired by	: System Administrator
Date Processed	: 11/2/2022 7:17:43 PM	Processed by	: System Administrator

<Chromatogram>

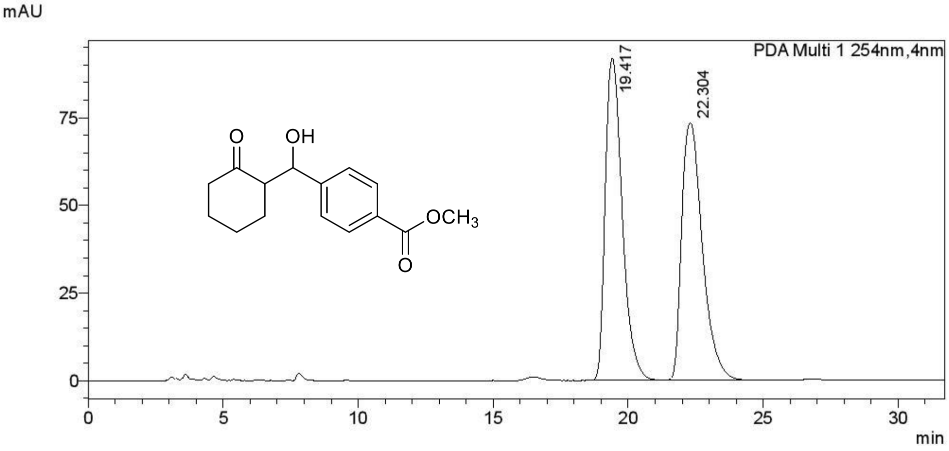

<Peak Table>

PDA Ch1 254nm

Peak#	Ret. Time	Area	Height	Area%
1	19.417	3982137	91586	50.486
2	22.304	3905444	73306	49.514
Total		7887580	164893	100.000

C:\Users\Shimadzu\Desktop\SZ\HPLC\Runs\SSZ02 RACE 5%IPAnHex 20uL 1mLmin 02nov22.lcd

Figure S29. HPLC trace of racemic aldol product **4c** (above).

SHIMADZU LabSolutions

Analysis Report

<Sample Information>

Sample Name	: HAB-I47 5%IPAnHex 20uL 1 mLmin 03Feb23		
Sample ID	:		
Data Filename	: HAB-I47 5%IPAnHex 20uL 1 mLmin 03Feb23.lcd		
Method Filename	: trial.lcm		
Batch Filename	:		
Vial #	: 1-3	Sample Type	: Unknown
Injection Volume	: 20 uL		
Date Acquired	: 2/3/2023 5:54:48 PM	Acquired by	: System Administrator
Date Processed	: 2/3/2023 6:25:54 PM	Processed by	: System Administrator

<Chromatogram>

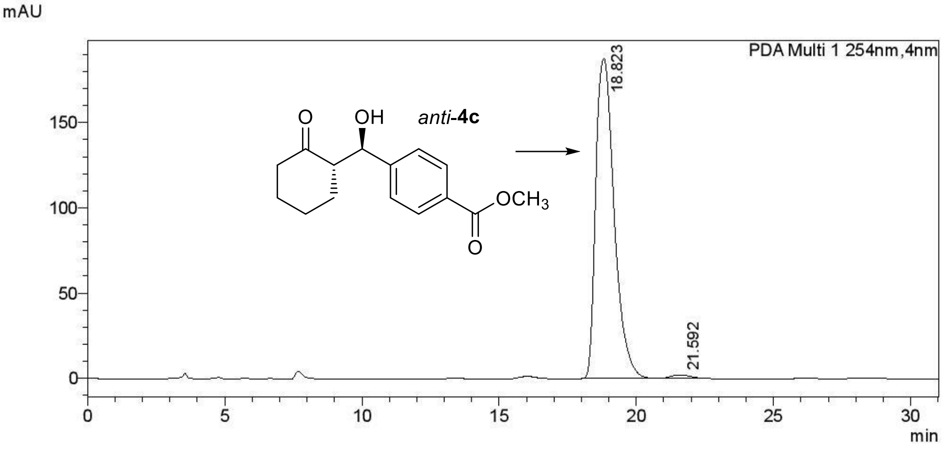

<Peak Table>

PDA Ch1 254nm

Peak#	Ret. Time	Area	Height	Area%
1	18.823	8745306	187688	99.030
2	21.592	85680	1897	0.970
Total		8830985	189585	100.000

C:\Users\Shimadzu\Desktop\Hanaa\HAB-I47 5%IPAnHex 20uL 1 mLmin 03Feb23.lcd

Figure S30. HPLC trace of enantioenriched *anti*-aldol (major) product **4c** (above).

Table 2, entry 8: Competition reaction between cyclohexanone and chloroacetone for the limiting reactant benzaldehyde

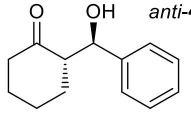

anti-**4d**

(*S*)-2-((*R*)-hydroxy(phenyl)methyl)cyclohexanone

To a clean screw cap V-shaped reaction vessel (5.0 mL) equipped with a pyramidal stir bar, were added cyclohexanone (MW= 98.14, 2 equiv, 3.0 mmol, 294.4 mg, density= 0.947 g/mL, 310.9 µL), chloroacetone (MW= 92.52, 2.00 equiv, 3.00 mmol, 277.6 mg, density= 1.162 g/mL, 238.9 µL), purified benzaldehyde (MW= 106.12, 1.00 equiv, 1.50 mmol, 159.2 mg, density= 1.044 g/mL, 152.5 µL), and *trans*-4-(*tert*-butyldiphenylsilyloxy)-L-proline catalyst (MW= 369.54, 5.0 mol%, 0.075 mmol, 27.7 mg) in the stated order. Within a minute of adding the catalyst, deoxygenated distilled water (MW= 18.02, 15 equiv, 22.5 mmol, 405.5 µL) was added. Chloroacetone is light-sensitive and aluminum foil was used to exclude light. Within 10 sec the solid catalyst fully dissolved leaving a transparent biphasic solution. The resulting heterogenous solution was rigorously stirred for 42 h such that an emulsion was always noted, albeit without causing splashing against the vessel walls, by doing so reproducible yield data was achieved.

This compound has been synthesized by many research groups, one example is:
Hayashi, Y.; Sumiya, T.; Takahashi, J.; Gotoh, H.; Urushima, T.; Shoji, M. Highly diastereo- and enantioselective direct aldol reactions in water. *Angew. Chem. Int. Ed.* **2006**, *45*, 958–961.

Crude product ^{1}H NMR analysis (**Figure S31**, see below) allowed the following diastereo- and chemoselectivity ratios to be determined.

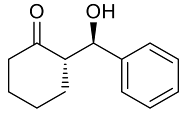

anti-aldol (major)

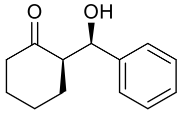

syn-aldol (minor)

Diastereoselectivity: The *anti*/*syn* aldol ratio was determined as 10:1 by comparing the *anti*-aldol product resonance at 4.78 ppm (d, representing the benzylic proton) and the *syn*-aldol product resonance at 5.39 ppm (bs, representing the benzylic proton). For literature regarding the *syn*-aldol product chemical shift, see page 2 of the Supporting Information within: N. Mase, Y. Nakai, N. Ohara, H. Yoda, K. Takabe, F. Tanaka, C. F. Barbas III, *J. Am. Chem. Soc.* **2006**, *128*, 734–735.

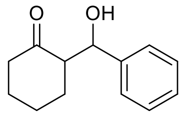

aldol products

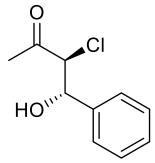

Knoevenagel *anti*-aldol product

Chemoselectivity: The term regioselectivity is more appropriate for this aldol *versus* aldol competition reaction. Despite this the chloroacetone product is labeled as the Knoevenagel product, and only the Knoevenagel *anti*-aldol product was observed. The aldol/Knoevenagel aldol product ratio was determined to be 11:1, based on the addition of the *anti*-aldol product resonance at 4.78 ppm (d, representing the benzylic proton) and the *syn*-aldol product resonance at 5.39 ppm (bs, representing the benzylic proton) *versus* the Knoevenagel aldol product resonance at 5.03 ppm (d, representing the benzylic proton). For literature regarding the chloroacetone aldol product chemical shift, see page S26 of the Supporting Information of the following reference: A. Martínez-CastaÇeda, B. Poladura, H. Rodríguez-Solla, C. Concellón, V. d. Amo, *Chem. Eur. J.* **2012**, *18*, 5188 – 5190. The absolute stereochemistry of the shown Knoevenagel *anti*-aldol product is assumed based on reports that L-proline templated catalysts form it.

The methyl group of the *Z*- and E-Knoevenagel condensation products of chloroacetone with benzaldehyde (not shown) respectively appear at 2.50 and 2.27 ppm and the alkene proton is found at either 7.72 ppm (Z-isomer) or 7.17 (E-isomer), see page 2643 within: K.-M. Kim, I.-H. Park, *Synthesis* **2004**, *16*, 2641–2644. Examination of our crude ^{1}H NMR spectrum shows that neither the E or Z-Knoevenagel condensation products are present.

Purification and yield: Silica gel chromatography (35 mm column outer diameter, 16 cm silica bed height) was performed using gradient elution (10 to 20 vol% ethyl acetate in petroleum ether). The crude product was loaded onto the column in a minimum volume of dichloromethane. The *anti*-aldol product **4d** was isolated as a single diastereomeric as a light-yellow oil (180.7 mg, MW= 204.27, 0.88 mmol, 59% yield.

TLC: R_f=0.34, ethyl acetate/petroleum ether (1:4)

95% *ee*: Chiralpak OD-H, nHexane/*i*PrOH (90:10), flow rate= 0.5 mL/min, λ = 221 nm, *anti*-aldol product t_{major} = 15.8 min, t_{minor}= 23.1 min (**Figure S34**).

^{1}H NMR (400 MHz, $CDCl_3$) (ppm) *anti*-aldol product **4d**: δ 7.27-7.37 (m, 5H), 4.78 (dd, 1H, J= 8.8 Hz, 2.6 Hz), 3.95 (d, 1H, *OH,* J= 2.8 Hz), 2.57-2.67 (m, 1H), 2.44-2.52 (m, 1H), 2.31-2.41 (m, 1H), 2.04-2.13 (m, 1H), 1.74-1.83 (m, 1H), 1.62-1.73 (m, 1H) 1.48-1.61 (m, 2H), 1.27-1.36 (m, 1H)

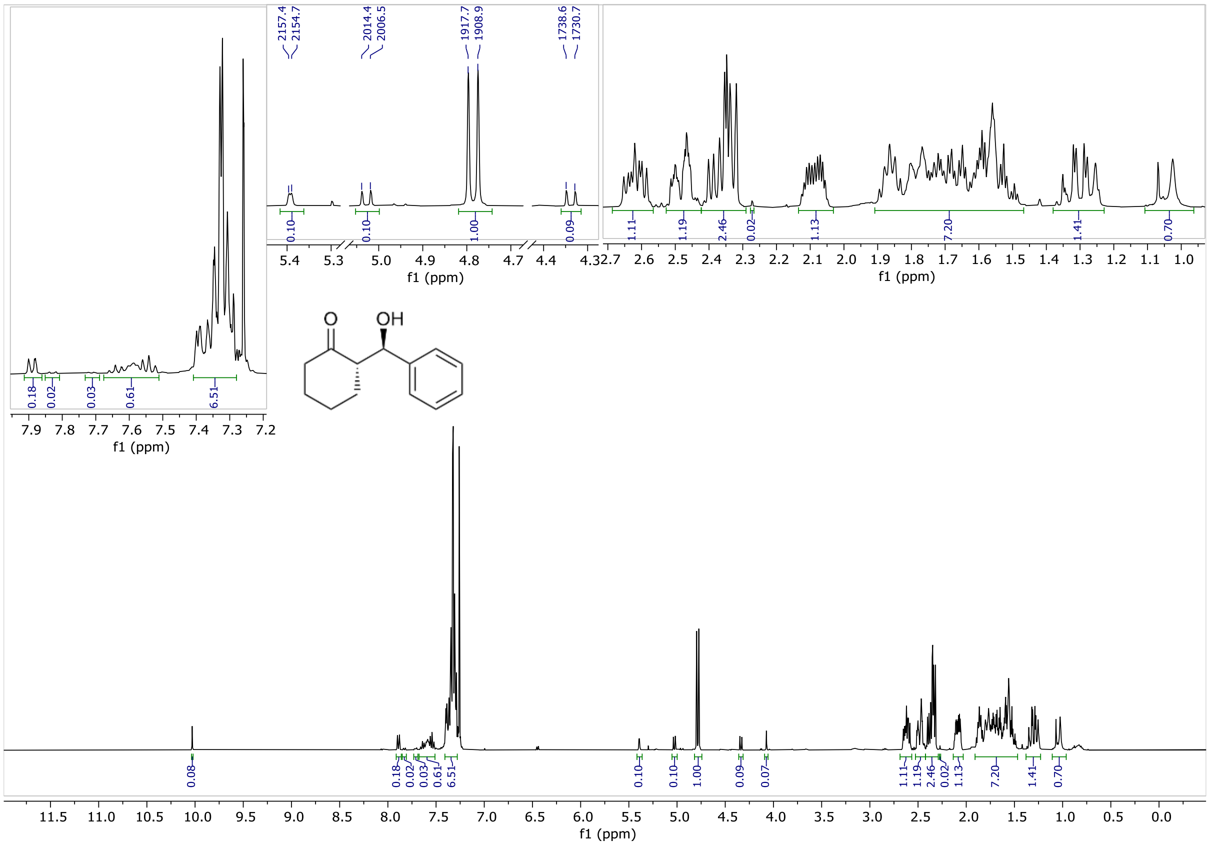

Figure S31. Crude ^{1}H NMR spectrum after high vacuum for 1 h (above).

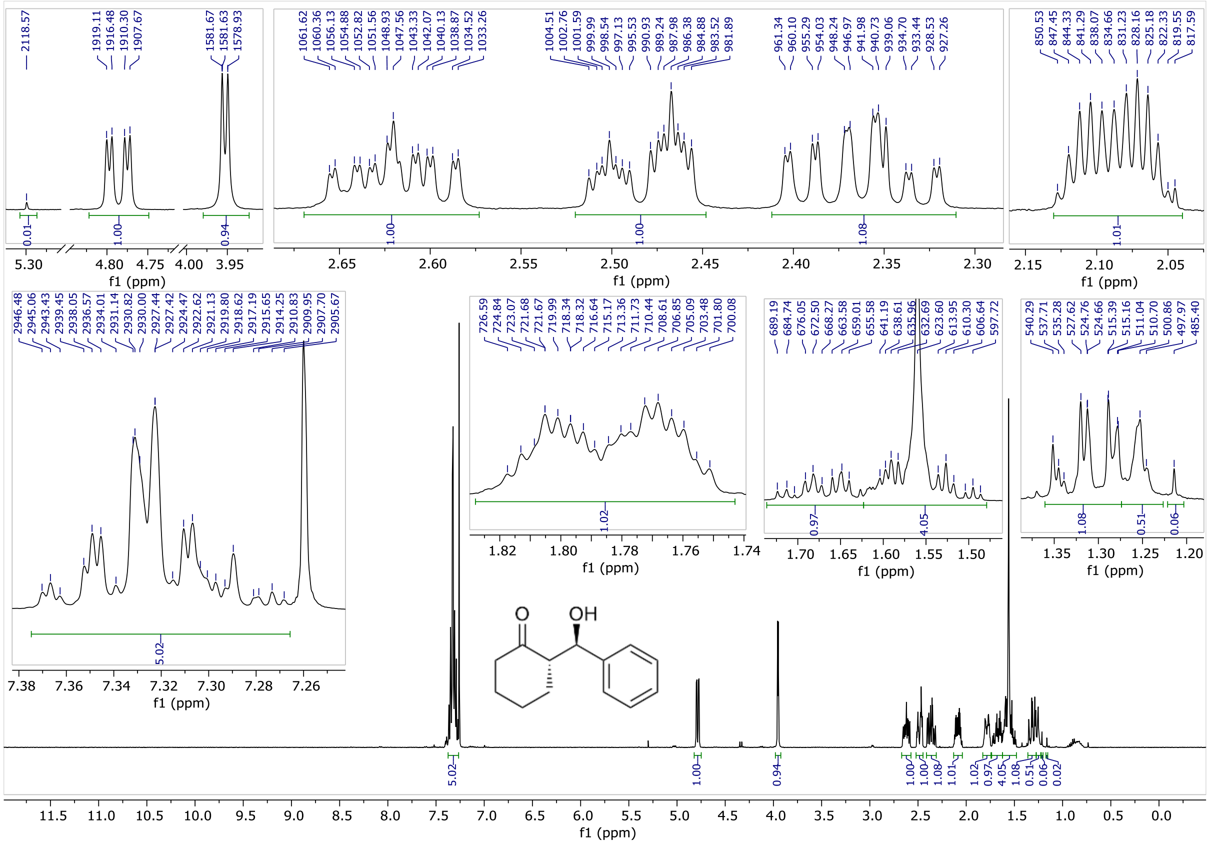

Figure S32. ^{1}H NMR spectrum of the purified *anti*-aldol (major) product **4d** (above).

SHIMADZU LabSolutions

Analysis Report

<Sample Information>

Sample Name	: HAB-I53 RACE 10%IPAnHex 20uL 0.5 mLmin 2mgmL 03Mar23		
Sample ID	:		
Data Filename	: HAB-I53 RACE 10%IPAnHex 20uL 0.5 mLmin 2mgmL 03Mar23.lcd		
Method Filename	: trial.lcm		
Batch Filename	:		
Vial #	: 1-5	Sample Type	: Unknown
Injection Volume	: 20 uL		
Date Acquired	: 3/3/2023 2:51:49 PM	Acquired by	: System Administrator
Date Processed	: 3/3/2023 3:23:24 PM	Processed by	: System Administrator

<Chromatogram>

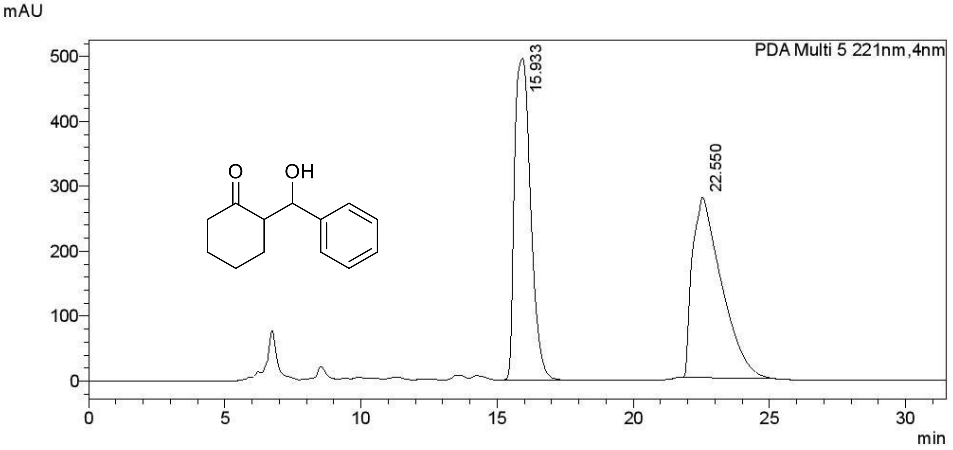

<Peak Table>

PDA Ch5 221nm

Peak#	Ret. Time	Area	Height	Area%
1	15.933	20501576	496127	49.736
2	22.550	20719405	278399	50.264
Total		41220981	774526	100.000

C:\Users\Shimadzu\Desktop\Hanaa\HAB-I53 RACE 10%IPAnHex 20uL 0.5 mLmin 2mgmL 03Mar23.lcd

Figure S33. HPLC trace of racemic aldol product **4d** (above).

Analysis Report

SHIMADZU LabSolutions

<Sample Information>

Sample Name : HAB-I52 10%IPAnHex 20uL 0.5 mLmin 2mgmL 03Mar23
Sample ID :
Data Filename : HAB-I52 10%IPAnHex 20uL 0.5 mLmin 2mgmL 03Mar23.lcd
Method Filename : trial.lcm
Batch Filename :
Vial # : 1-4 Sample Type : Unknown
Injection Volume : 20 uL
Date Acquired : 3/3/2023 4:43:51 PM Acquired by : System Administrator
Date Processed : 3/3/2023 5:14:14 PM Processed by : System Administrator

<Chromatogram>

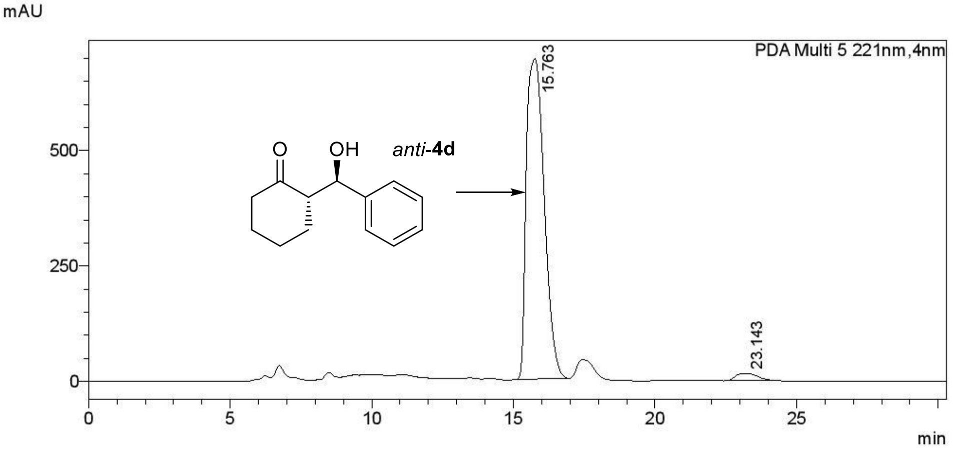

<Peak Table>

PDA Ch5 221nm

Peak#	Ret. Time	Area	Height	Area%
1	15.763	30359611	695412	97.307
2	23.143	840278	15366	2.693
Total		31199889	710778	100.000

Figure S34. HPLC trace of enantioenriched *anti*-aldol (major) product **4d** (above).

Table 2, entry 9: Competition reaction between cyclohexanone and 4-nitroacetophenone for the limiting reactant 4-(trifluoromethyl)benzaldehyde.

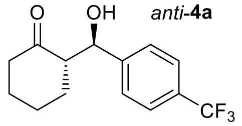
anti-**4a**

(*S*)-2-((*R*)-hydroxy(4-(trifluoromethyl)phenyl)methyl)cyclohexan-1-one

To a clean screw cap V-shaped reaction vessel (5.0 mL) equipped with a pyramidal stir bar, were added cyclohexanone (MW= 98.14, 1.5 equiv, 2.25 mmol, density= 0.947 g/mL, 233 µL), 4-nitroacetophenone (MW= 165.15, 1.5 equiv, 2.25 mmol, 371.6 mg), purified 4-(trifluoromethyl)benzaldehyde (MW= 174.12, 1.0 equiv, 1.5 mmol, 261.2 mg, density= 1.275 g/mL, 205 µL), and *trans*-4-(*tert*-butyldiphenylsilyloxy)-L-proline catalyst (MW= 369.54, 5.0 mol%, 0.075 mmol, 27.7 mg) in the stated order. Within a minute of adding the catalyst, deoxygenated distilled water (MW= 18.02, 15.0 equiv, 22.5 mmol, 405.5 µL) was added. The resulting heterogenous solution was rigorously stirred for 36 h. The 4-nitroacetophenone did not fully dissolve, but was efficiently stirred with the liquids. As the reaction time progressed, the amount of solid material appeared to decrease. The reaction solution was efficiently stirred albeit without causing the contents to splash against the vessel walls, by doing so reproducible yield data was achieved.

This compound has been synthesized by many research groups, one example is:
Miura, T.; Imai, K.; Ina, M.; Tada, N.; Imai, N.; Itoh, A. Direct asymmetric aldol reaction with recyclable fluorous organocatalyst. *Org. Lett.* **2010**, *12*, 1620–1623.

Crude product ^{1}H NMR analysis (**Figure S35**, see below) allowed the following diastereo- and chemoselectivity ratios to be determined.

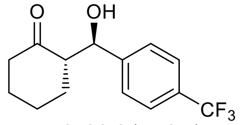
anti-aldol (major)

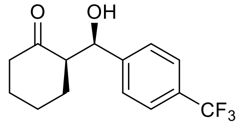
syn-aldol (minor)

Diastereoselectivity: The *anti*/*syn* aldol product ratio was determined to be 14.3:1, by comparing the *anti*-aldol product resonance at 4.85 ppm (d, representing the benzylic proton) and the *syn*-aldol product resonance at 5.44 ppm (d, representing the benzylic proton). For literature regarding the *syn*-aldol product chemical shifts, see page 14 of the Supporting Information within: J. Gao, S. Bai, Q. Gao, Y. Liu, Q. Yang, *Chem. Commun*. **2011**, *47*, 6716-6718.

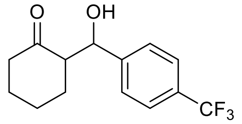
aldol products

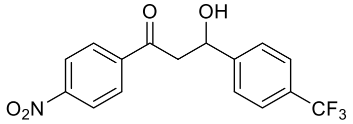
alternative aldol product

Chemoselectivity: The term regioselectivity is more appropriate for this aldol *versus* aldol competition reaction. This reaction is 100% regioselective for cyclohexanone, *i.e.*, we could not identify any aldol or aldol condensation product from the reaction of 4-nitroacetophenone.

Purification and yield: Silica gel chromatography (35 mm column outer diameter, 16 cm silica bed height) was performed using gradient elution (10 to 20 vol% ethyl acetate in petroleum ether). The crude product was loaded onto the column in a minimum volume of CH_2Cl_2. The *anti*-aldol product **4a** was isolated as a single diastereomer as a light-yellow solid (336.1 mg, MW= 272.26, 1.23 mmol, 82% yield).

TLC: R_f= 0.20, ethyl acetate/petroleum ether (15:85).

98% *ee*: Chiralpak OD-H chiral HPLC column, *i*PrOH/*n*-hexane (20:80), flow rate = 0.5 mL/min, λ= 216 nm, *anti*-aldol product t_{major}= 11.1 min, t_{minor}= 13.1 min was observed (**Figure S38**).

^{1}H NMR (400 MHz, $CDCl_3$) (ppm) *anti*-aldol (major) product **4a**: δ 7.61 (d, 2H, J= 8.3 Hz), 7.44 (d, 2H, J= 8.1 Hz), 4.85 (dd, 1H, J= 3.0 Hz), 4.03 (d, 1H, J= 2.9 Hz), 2.54-2.65 (m, 1H), 2.45-2.53 (m, 1H), 2.30-2.42 (m, 1H), 2.05-2.16 (m, 1H), 1.76-1.87 (m, 1H), 1.48-1.74 (m, 3H), 1.27-1.41 (m, 1H).

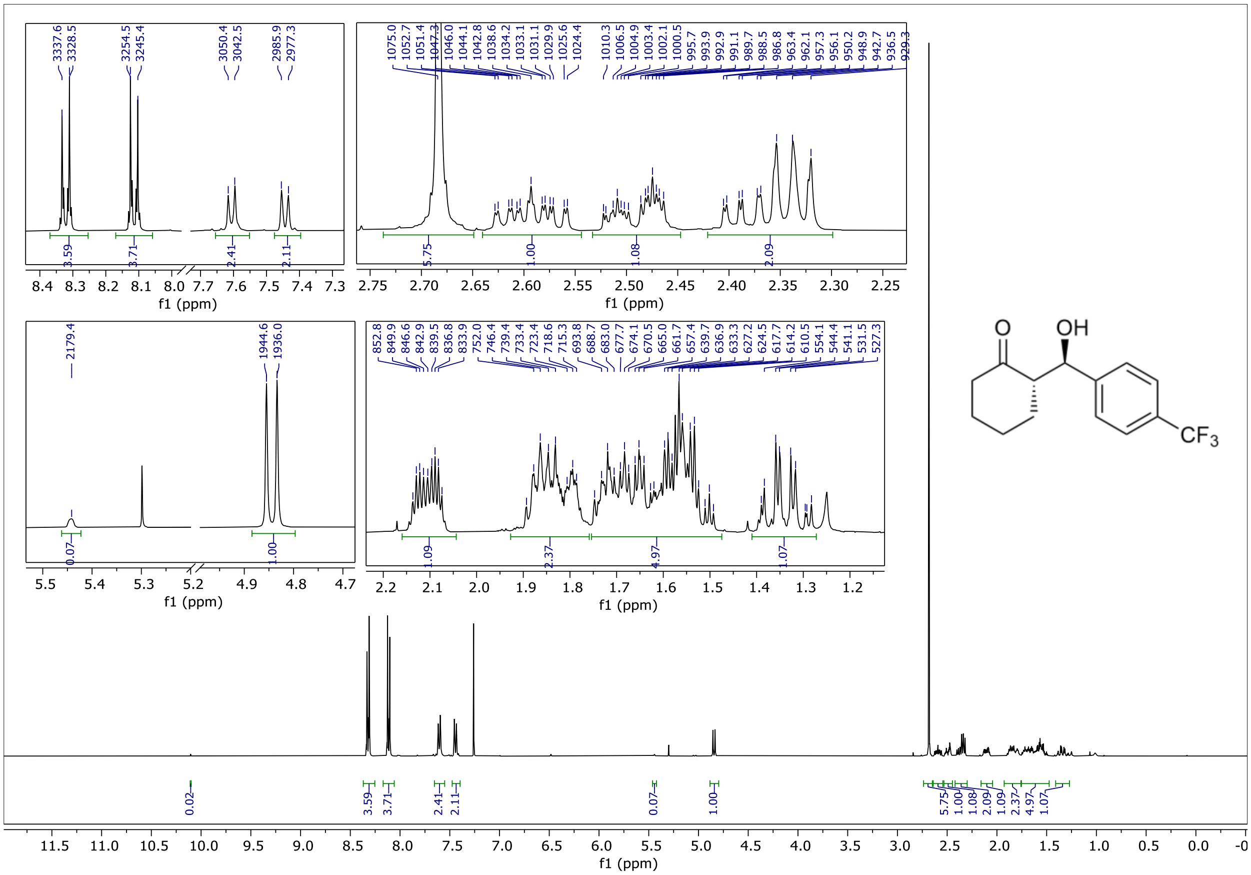

Figure S35. Crude ^{1}H NMR spectrum after high vacuum drying overnight (above).

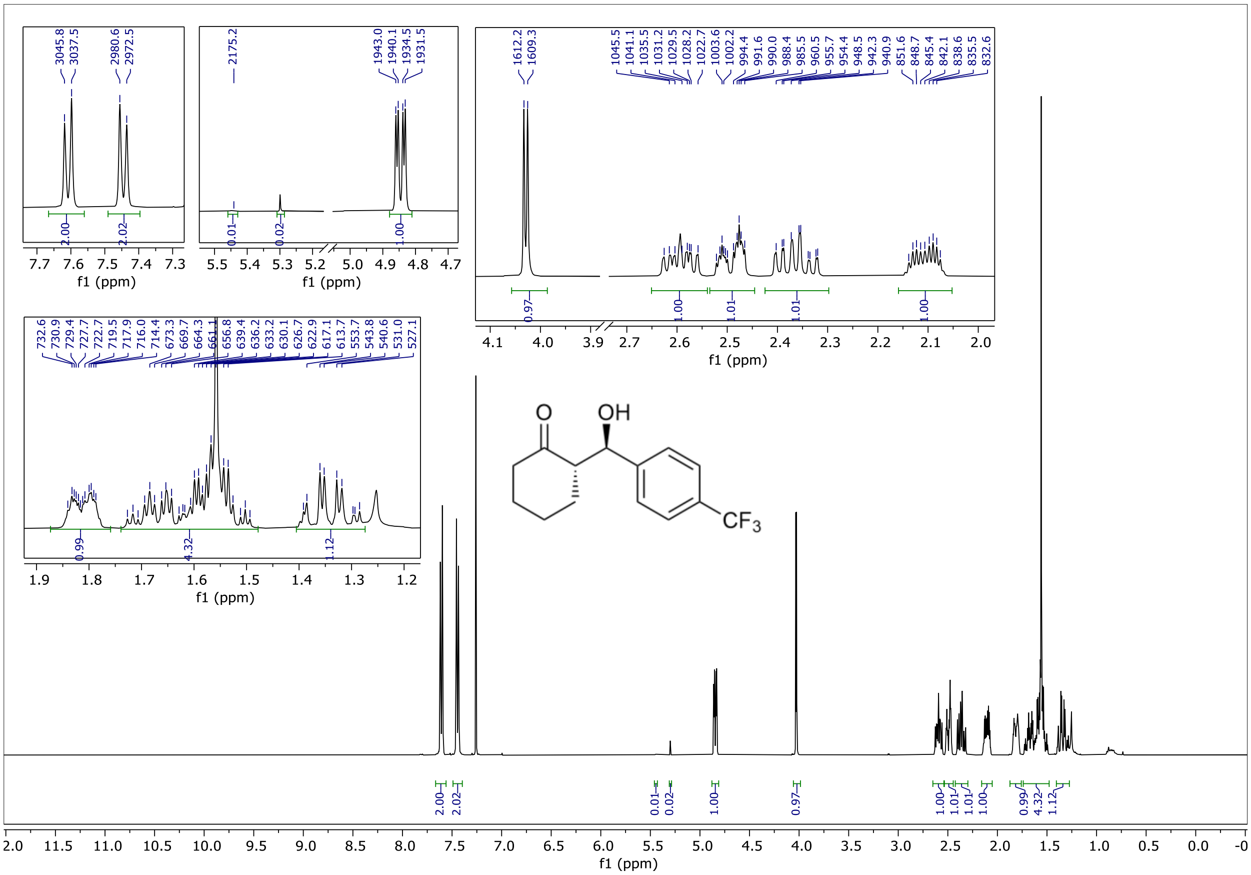

Figure S36. ^{1}H NMR spectrum of the purified *anti*-aldol product **4a** (above).

SHIMADZU LabSolutions

Analysis Report

<Sample Information>

Sample Name	: HC-I-26 20%IPAnHex 20uL 0.5 mLmin 08Feb23 1mgmL		
Sample ID	:		
Data Filename	: HC-I-26 20%IPAnHex 20uL 0.5 mLmin 08Feb23 1mgmL.lcd		
Method Filename	: trial.lcm		
Batch Filename	:		
Vial #	: 1-4	Sample Type	: Unknown
Injection Volume	: 20 uL		
Date Acquired	: 2/8/2023 3:15:20 PM	Acquired by	: System Administrator
Date Processed	: 2/8/2023 3:39:35 PM	Processed by	: System Administrator

<Chromatogram>

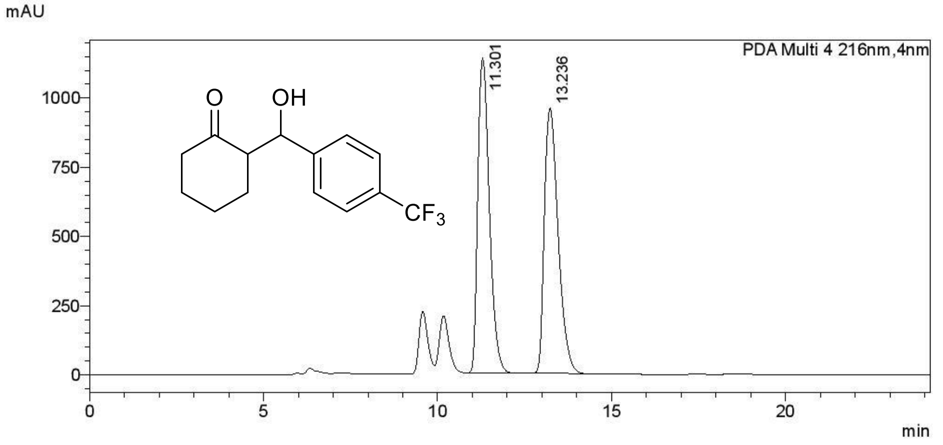

<Peak Table>

PDA Ch4 216nm

Peak#	Ret. Time	Area	Height	Area%
1	11.301	26277677	1138787	49.940
2	13.236	26341028	958736	50.060
Total		52618705	2097523	100.000

C:\Users\Shimadzu\Desktop\Hayley\HC-I-26 20%IPAnHex 20uL 0.5 mLmin 08Feb23 1mgmL.lcd

Figure S37. HPLC trace of racemic aldol product **4a** (above).

SHIMADZU LabSolutions

Analysis Report

<Sample Information>

Sample Name	: HC-I-30-20%IPAnHex 20uL 0.5 mLmin 20Feb23 1mgmL		
Sample ID	:		
Data Filename	: HC-I-30-20%IPAnHex 20uL 0.5 mLmin 20Feb23 1mgmL.lcd		
Method Filename	: trial.lcm		
Batch Filename	:		
Vial #	: 1-32	Sample Type	: Unknown
Injection Volume	: 20 uL		
Date Acquired	: 2/20/2023 5:10:28 PM	Acquired by	: System Administrator
Date Processed	: 2/20/2023 5:35:11 PM	Processed by	: System Administrator

<Chromatogram>

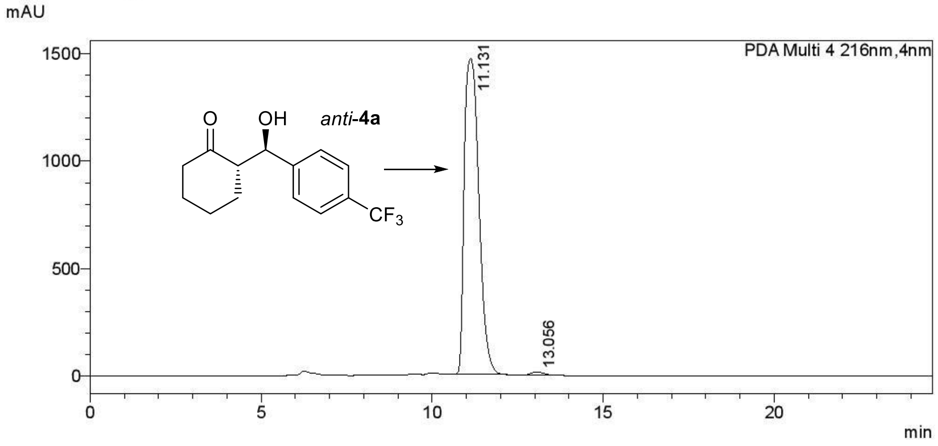

<Peak Table>

PDA Ch4 216nm

Peak#	Ret. Time	Area	Height	Area%
1	11.131	43674340	1474783	99.178
2	13.056	362026	13088	0.822
Total		44036367	1487871	100.000

C:\Users\Shimadzu\Desktop\Hayley\HC-I-30-20%IPAnHex 20uL 0.5 mLmin 20Feb23 1mgmL.lcd

Figure S38. HPLC trace of the enantioenriched *anti*-aldol (major) product **4a** (above).

Table 2, entry 10: Competition reaction between 4-methylcyclohexanone and acetylacetone for the limiting reactant 4-nitrobenzaldeyhde.

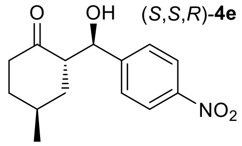
(*S*,*S*,*R*)-**4e**

(2*S*,4*S*)-2-((*R*)-hydroxy(4-nitrophenyl)methyl)-4-methylcyclohexanone:

To a clean screw cap V-shaped reaction vessel (5.0 mL) equipped with a pyramidal stir bar, were added 4-methylcyclohexanone (MW= 112.17, 2.00 equiv, 3.00 mmol, 337 mg, density= 0.914 g/mL, 368 µL), acetylacetone (MW= 100.12, 2.00 equiv, 3.00 mmol, 300 mg, density= 0.975 g/mL, 308 µL), *trans*-4-(tert-butyldiphenylsilyloxy)-L-proline catalyst (MW= 369.54, 5.0 mol%, 0.075 mmol, 27.7 mg), and mortar and pestle ground 4-nitrobenzaldehyde (MW= 151.12, 1.00 equiv, 1.50 mmol, 227 mg) in the stated order. Within a minute of adding the catalyst, distilled water (MW= 18.02, 15 equiv, 22.5 mmol, 405.5 µL) was added. The resulting heterogenous solution was rigorously stirred for 28 h such that an emulsion was always noted. The reaction solution was efficiently stirred albeit without causing the contents to splash against the vessel walls, by doing so reproducible yield data was achieved.

This compound has been synthesized by many research groups, one example is:
Li, S.; Wu, C.; Long, X.; Fu, X.; Chen, G.; Liu, Z. Simple proline derivatives as recoverable catalysts for the large-scale stoichiometric aldol reactions. *Catal. Sci. Technol.* **2012**, *2*, 1068–1071.

Crude product ^{1}H NMR analysis (**Figure S39**, see below) allowed the following diastereo- and chemoselectivity ratios to be determined.

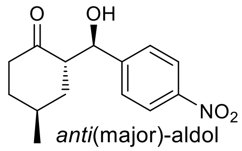

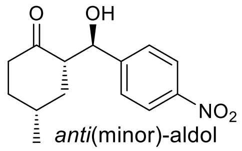

Diastereoselectivity (*anti*/*anti*): The *anti*(major)-/*anti*(minor)-aldol ratio was determined as 16.7:1. See the resonances at 4.92 ppm (d, representing the benzylic proton of the *anti*(major)-product) and 4.87 ppm (d, representing the benzylic proton of the *anti*(minor)-aldol product). For literature regarding the *anti*(minor)-aldol product chemical shift, see the manuscript text and page 82 of the Supporting Information within: T. C. Nugent, P. Spiteller, I. Hussain, H. A. E. D. Hussein, F. Tehrani Najafian, *Adv. Synth. Catal.* **2016**, *358*, 3706–3713.

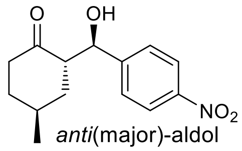

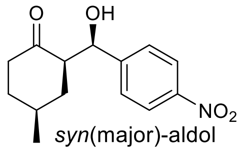

Diastereoselectivity (*anti*/*syn*):
The *anti*(major)/*syn(major)* aldol ratio was determined as 12.5:1. See the *anti*(major)-aldol product resonance at 4.92 ppm (d, representing the benzylic proton) and the the *syn*(major)-aldol product at 5.49 ppm (d, representing the benzylic proton). For literature regarding the *syn*-aldol product, see page 3676 within the following reference: Y. Qian, X. Zheng, and Y. Wang, *Eur. J. Org. Chem.* **2010**, 3672-3677.

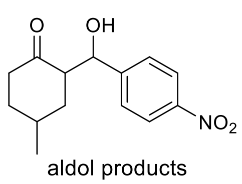
aldol products

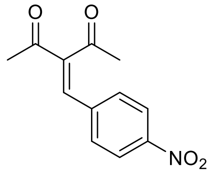
Knoevenagel product

Chemoselectivity: The *anti-*/*syn*-aldol/Knoevenagel product ratio was determined to be 9.9:1 based on the addition of all aldol product resonances at 5.49, 5.47, 4.92, and 4.87 ppm (doublets representing the benzylic protons) and the Knoevenagel product resonance at 2.28 ppm (a singlet representing one of the methyl groups). For the literature regarding the Knoevenagel condensation product chemical

shift, see page 2258 within the following reference: Y. Zhang, C. Sun, J. Liang, Z. Shang, *Chin. J. Chem*. **2010**, *28*, 2255-2259.

Purification and yield: Silica gel chromatography (35 mm column outer diameter, 16 cm silica bed height) was performed using gradient elution (20 to 30 vol% ethyl acetate in petroleum ether). The crude product was loaded onto the column in a minimum volume of dichloromethane. The *anti*(major)-aldol product (*S*,*S*,*R*)-**4e** was isolated as a single diastereomer as a light-yellow solid (255.1 mg, MW= 263.29, 0.969 mmol, 65% yield). The ^{1}H NMR of the purified product is contaminated with minute quantities (<3%) of the Knoevenagel product, see the resonance at 2.28 ppm (a singlet representing one of the methyl groups of the Knoevenagel product).

TLC: R_f=0.27, ethyl acetate/petroleum ether (2:3)

98% *ee*: Chiralpak OD-H, nHexane/*i*PrOH (95:5), flow rate= 1.0 mL/min, λ = 254 nm, major *anti*-aldol product t_{major} = 34.7 min, t_{minor}= 46.0 min (**Figure S42**).

^{1}H NMR (400 MHz, $CDCl_3$)(ppm) *anti*-aldol (major) product (*S*,*S*,*R*)-**4e**: δ 8.22 (d, 2H, J= 8.9 Hz), 7.50 (d, 2H, J= 8.3 Hz), 4.91 (d, 1H, J= 8.4 Hz), 2.70-2.79 (m, 1H), 2.49-2.60 (m, 1H), 2.35-2.44 (m, 1H), 2.02-2.15 (m,1H), 1.88-2.00 (m, 1H), 1.75-1.85 (m,1H), 1.49-1.65 (m, 1H), 1.27-1.36 (m, 1H), 1.06 (d, 3H, J= 7.0 Hz).

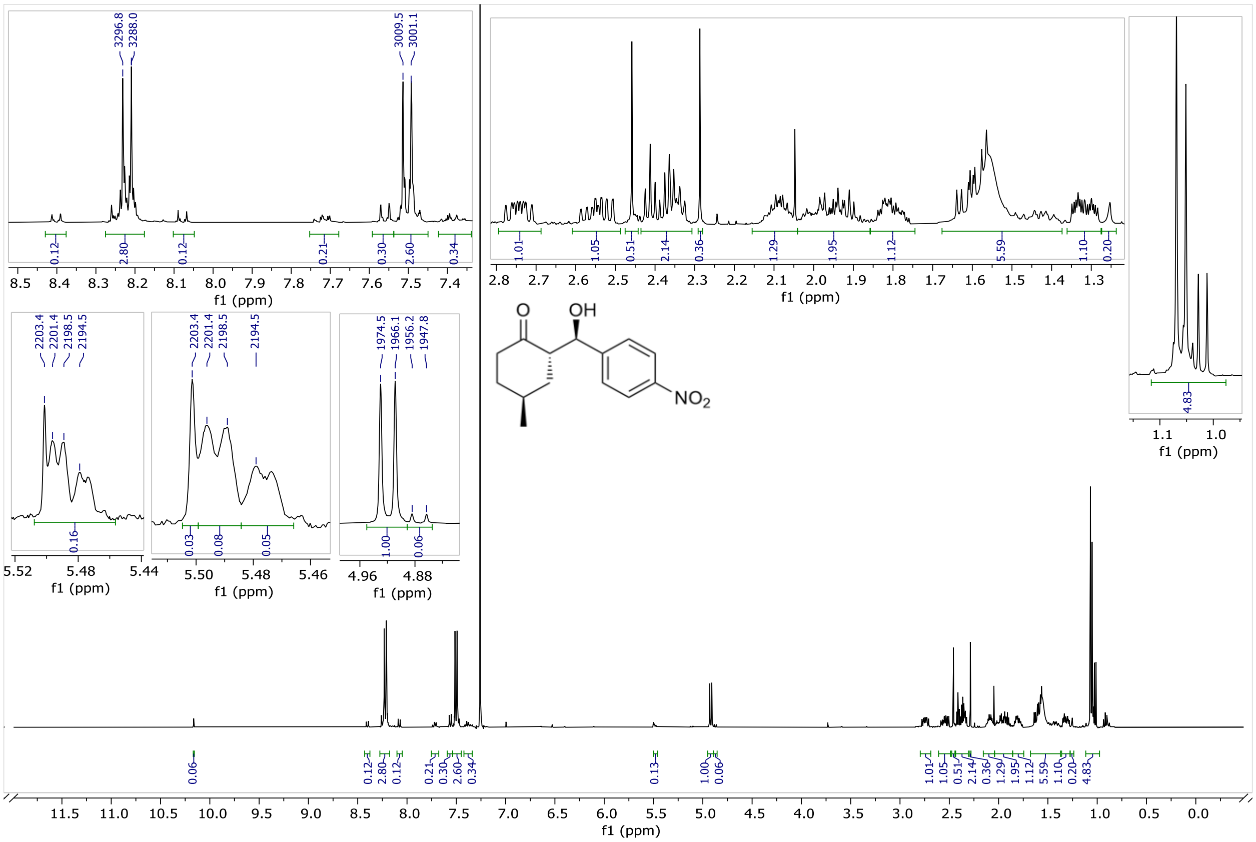

Figure S39. Crude ^{1}H NMR spectrum after high vacuum drying overnight (above).

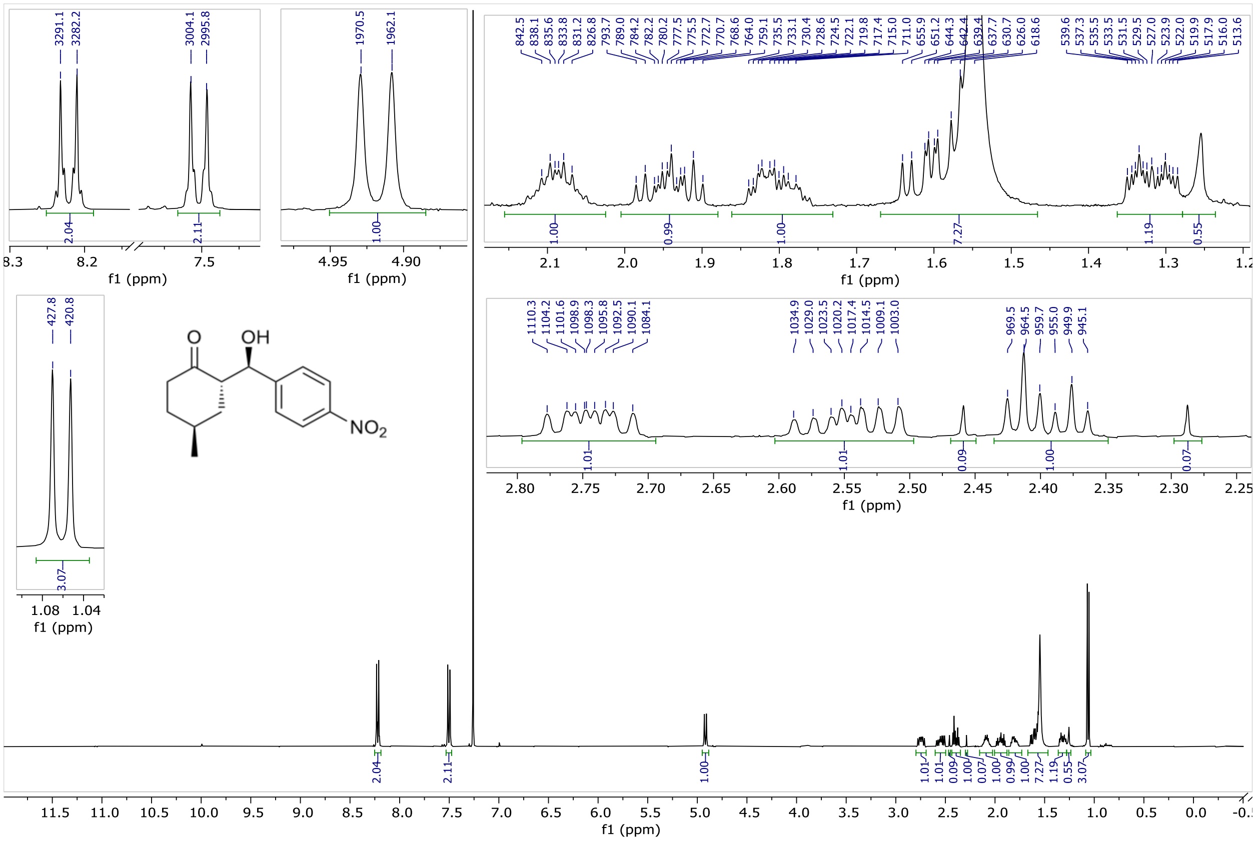

Figure S40. ^{1}H NMR spectrum of the purified *anti*-aldol product **4e** (above).

SHIMADZU LabSolutions

Analysis Report

<Sample Information>

Sample Name	: HAB21 Race 5%IPAnHex 10uL 1mLmin 10Aug		
Sample ID	:		
Data Filename	: HAB21 Race 5%IPAnHex 10uL 1mLmin 10Aug.lcd		
Method Filename	: trial.lcm		
Batch Filename	:		
Vial #	: 1-3	Sample Type	: Unknown
Injection Volume	: 10 uL		
Date Acquired	: 8/10/2022 1:43:42 PM	Acquired by	: System Administrator
Date Processed	: 8/10/2022 2:43:47 PM	Processed by	: System Administrator

<Chromatogram>

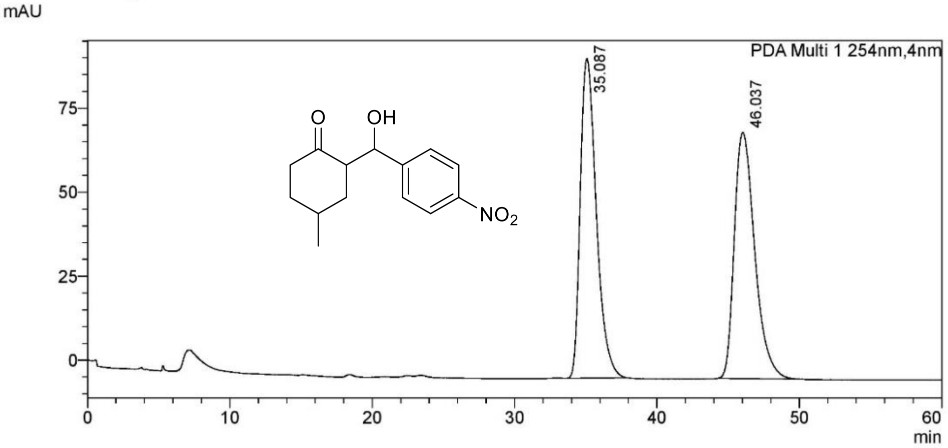

<Peak Table>

PDA Ch1 254nm

Peak#	Ret. Time	Area	Height	Area%
1	35.087	7421140	95157	50.429
2	46.037	7294940	73334	49.571
Total		14716080	168491	100.000

C:\Users\Shimadzu\Desktop\Hanaa\4methcyc4nitro Racemate\HAB21 Race 5%IPAnHex 10uL 1mLmin 10Aug.lcd

Figure S41. HPLC trace of racemic aldol product **4e** (above).

SHIMADZU LabSolutions **Analysis Report**

<Sample Information>

Sample Name	: HAB22 frac12-14 5%IPAnHex 20uL 1mLmin 11Aug
Sample ID	:
Data Filename	: HAB22 frac12-14 5%IPAnHex 20uL 1mLmin 11Aug.lcd
Method Filename	: trial.lcm
Batch Filename	:
Vial #	: 1-4
Injection Volume	: 20 uL
Date Acquired	: 8/11/2022 3:03:39 PM
Date Processed	: 8/11/2022 4:03:45 PM
Sample Type	: Unknown
Acquired by	: System Administrator
Processed by	: System Administrator

<Chromatogram>

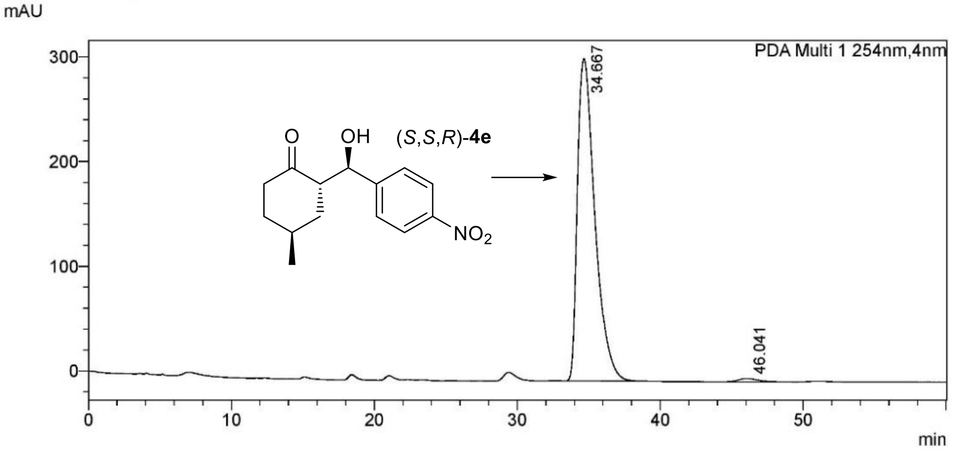

<Peak Table>

PDA Ch1 254nm

Peak#	Ret. Time	Area	Height	Area%
1	34.667	26307593	307917	98.904
2	46.041	291528	3202	1.096
Total		26599121	311119	100.000

C:\Users\Shimadzu\Desktop\Hanaa\HAB22 frac12-14 5%IPAnHex 20uL 1mLmin 11Aug.lcd

Figure S42. HPLC trace of the enantioenriched *anti*-aldol (major) product (*S*,*S*,*R*)-**4e**.

Table 2, entry 11: Competition reaction between cyclopentanone and acetylacetone for the limiting reactant 2-chlorobenzaldeyhde.

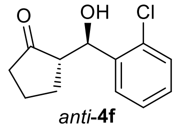

anti-**4f**

(S)-2-((R)-(2-chlorophenyl)(hydroxy)methyl)cyclopentan-1-one

To a clean screw cap V-shaped reaction vessel (5.0 mL) equipped with a pyramidal stir bar, were added cyclopentanone (MW= 84.12, 1.50 equiv, 2.25 mmol, density= 0.951 g/mL, 199 μL), acetylacetone (MW= 100.12, 1.50 equiv, 2.25 mmol, density= 0.975 g/mL, 231 μL), 2-chlorobenzaldehyde (MW= 140.57, 1.00 equiv, 1.50 mmol, density= 1.248 g/mL, 169 μL), and *trans*-4-(*tert*-butyldiphenylsilyloxy)-L-proline catalyst (MW= 369.54, 2.5 mol%, 0.0375 mmol, 13.9 mg) in the stated order. Within a minute of adding the catalyst, deoxygenated distilled water (MW= 18.02, 15 equiv, 22.5 mmol, 405 μL) was added. The catalyst (the only solid) fully dissolved within 20 min. Note that rigorous stirring was performed such that the reaction solution did not splash against the vessel walls, by doing so reproducible yield data was achieved. At 24 h stirring was arrested, a brown albeit transparent concentrated organic layer and a water layer were observed. Note screening reactions for this particular reaction showed that greater catalyst loading and/or longer reaction times resulted in reduced *dr*, see manuscript text.

This compound has been synthesized by many research groups, one example is:
Giacalone, F.; Gruttadauria, M.; Meo, P. L.; Riela, S.; Noto, R. New simple hydrophobic proline derivatives as highly active and stereoselective catalysts for the direct asymmetric aldol reaction in aqueous dedium. *Adv. Synth. Catal.* **2008**, *350*, 2747–2760.

Crude product ^{1}H NMR analysis (**Figure S43**, see below) allowed the following diastereo- and chemoselectivity ratios to be determined.

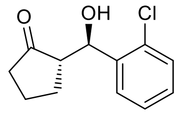

anti-aldol (major)

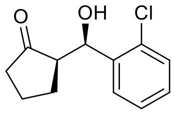

syn-aldol (minor)

Diastereoselectivity: The *anti*/*syn* aldol product ratio was determined to be 4:1, based on the *anti*-aldol product resonance at 5.31 ppm (d, representing the benzylic proton) and the *syn*-aldol product resonance at 5.69 ppm (d, representing the benzylic proton). For literature regarding the *syn*-aldol product chemical shift, see page 5 of the Supporting Information within: G. D. Yadav, S. Singh, *RCS Adv.* **2016**, *6,* 100459-100466.

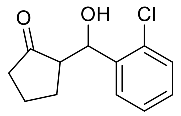

aldol products

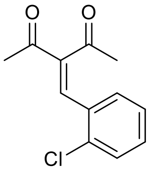

Knoevenagel product

Chemoselectivity: The aldol/Knoevenagel product ratio was determined to be >19:1, based on the addition of the *anti*-aldol product resonance at 5.31 ppm (d, representing the benzylic proton) and the *syn*- aldol product resonance at 5.69 ppm (d, representing the benzylic proton) *versus* the Knoevenagel product resonance at 7.79 ppm (the singlet representing the β-carbon of the enone. For literature regarding the Knoevenagel condensation product chemical shifts, see page 127 within: J. W. Patterson, J. T. Nelson, *J. Heterocyclic Chem*. **1988**, *25*, 125-128.

Purification and yield: Silica gel chromatography (35 mm column outer diameter, 16 cm silica bed height) was performed using gradient elution (5 to 15 vol% ethyl acetate in petroleum ether). The crude product was loaded onto the column in a minimum volume of CH_2Cl_2. The *anti-*/*syn*-aldol products **4f** was isolated as a light-brown liquid (316.3 mg, MW= 224.68, 1.41 mmol, 94% yield).

TLC: R_f = 0.27, EtOAc/petroleum ether (15:85)

>99% *ee*: Chiralpak OD-H chiral HPLC column, *i*PrOH/*n*-hexane (4:96), flow rate = 0.5 mL/min, λ= 221 nm, *anti*-aldol product t_{major}= 24.8 min, t_{minor}= 26.1 min. Due to the small area of the minor product it could not be accurately integrated in **Figure S46**, the minor product retention time shown here was therefore taken from the racemate HPLC (**Figure S45**). We also acknowledge that the racemate of the *anti*-aldol product is not baseline resolved. Thus, while we claim >99% *ee*, we cannot be 100% certain that this is correct. An indirect support of our high *ee* is literature stating a >99% *ee* when using proline or proline-based catalysts, see page 959 of the following reference: Y. Hayashi, S. Aratake, T. Itoh, T. Okano, T. Sumiya, M. Shoji, *Chem. Commun*. **2007**, 957–959. For literature stating a 98% *ee*, see page 2752 of the following reference: F. Giacalone, M. Gruttadauria, P. L. Meo, S. Riela, R. Noto, *Adv*. Synth*. Catal*. **2008**, *350*, 2747-2760.

^{1}H NMR (400 MHz, $CDCl_3$) (ppm) *anti*-aldol (major) product **4f**: δ 7.54-7.62 (m, 1H), 7.27-7.36 (m, 2H), 7.17-7.25 (m, 1H), 5.31 (d, 1H, J= 9.3 Hz), 4.54 (br, s, 1H), 2.25-2.51 (m, 3H), 1.92-2.07 (m, 1H), 1.61-1.82 (m, 3H).

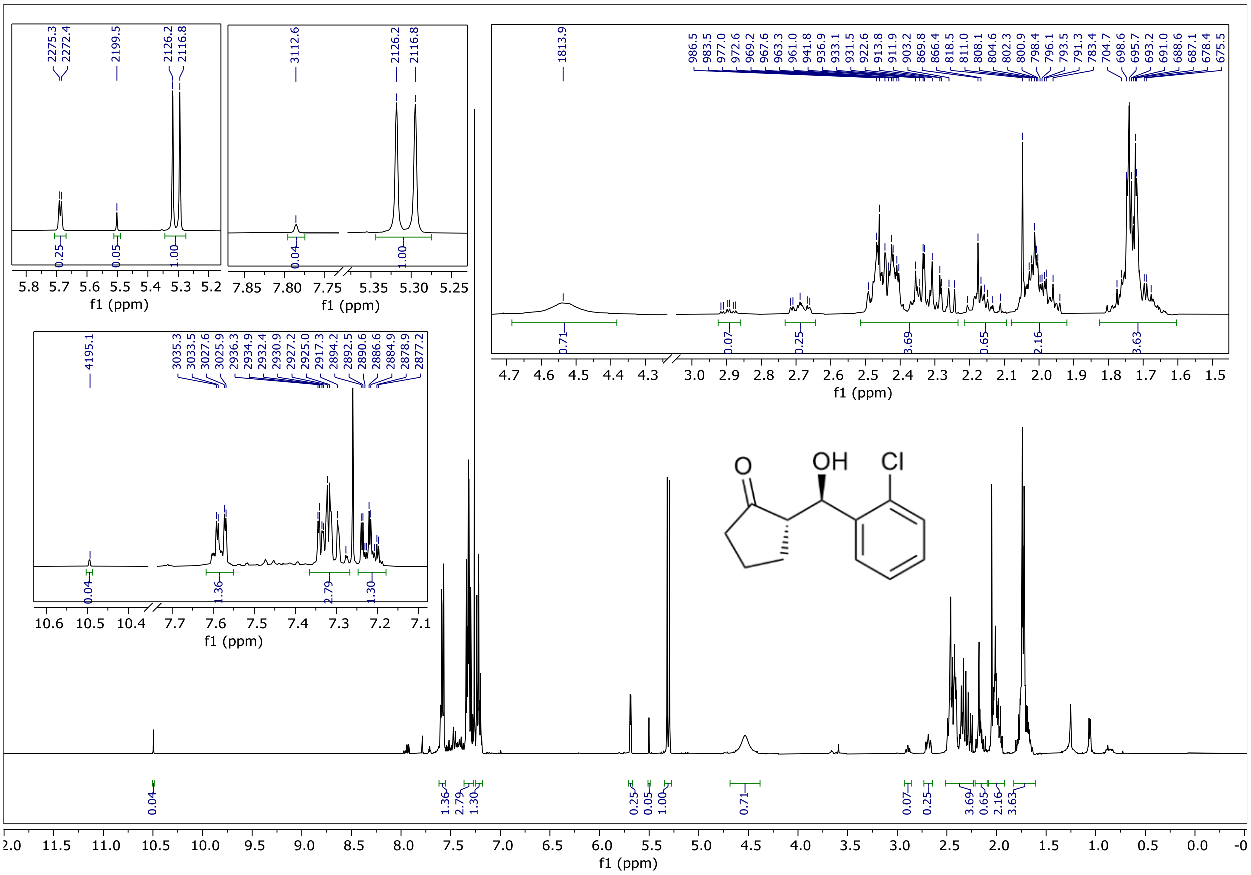

Figure S43. Crude ^{1}H NMR spectrum after high vacuum drying overnight (above).

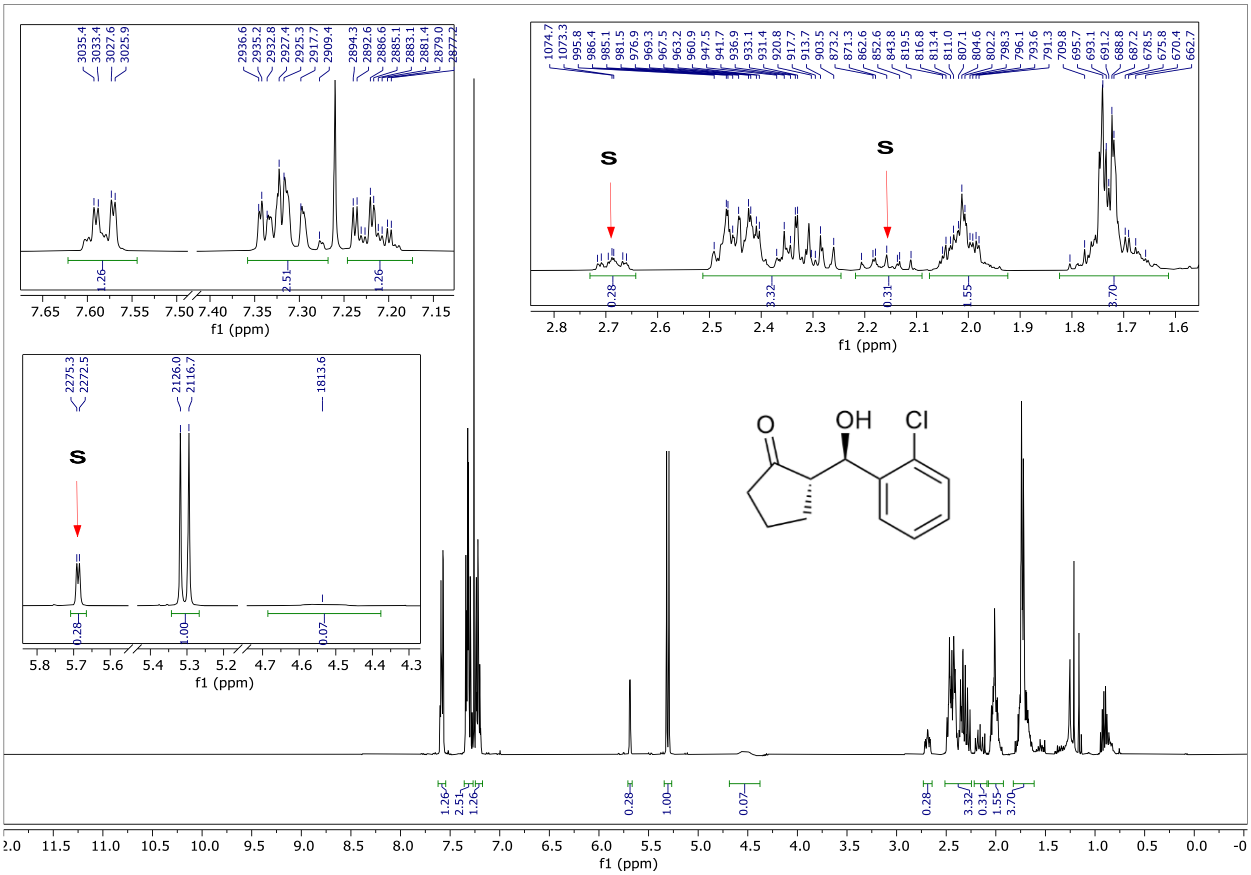

Figure S44. ^{1}H NMR spectrum of the purified *anti*-(major) and syn-aldol products **4f** (above). We did not attempt to separate these diastereomers during our chromatographic purification because the vast majority of literature reported isolating the *anti*- and *syn*-aldol products together for the yield determination. In some of the above expansions, red arrows labelled with an 'S' indicate some of the critical resonances from the minor *syn*-aldol product. For literature regarding the *syn*-aldol product chemical shifts, see page 5 of the Supporting Information within: G. D. Yadav, S. Singh, *RCS Adv.* **2016**, *6,* 100459-100466.

SHIMADZU LabSolutions Analysis Report

<Sample Information>

Sample Name	: HC-I-37 Race 4%IPAnHex 20uL 0.5 mLmin 22Mar23
Sample ID	:
Data Filename	: HC-I-37 Race 4%IPAnHex 20uL 0.5 mLmin 22Mar23.lcd
Method Filename	: trial.lcm
Batch Filename	:
Vial #	: 1-62
Sample Type	: Unknown
Injection Volume	: 20 uL
Date Acquired	: 3/22/2023 6:50:01 PM
Acquired by	: System Administrator
Date Processed	: 3/22/2023 7:21:45 PM
Processed by	: System Administrator

<Chromatogram>

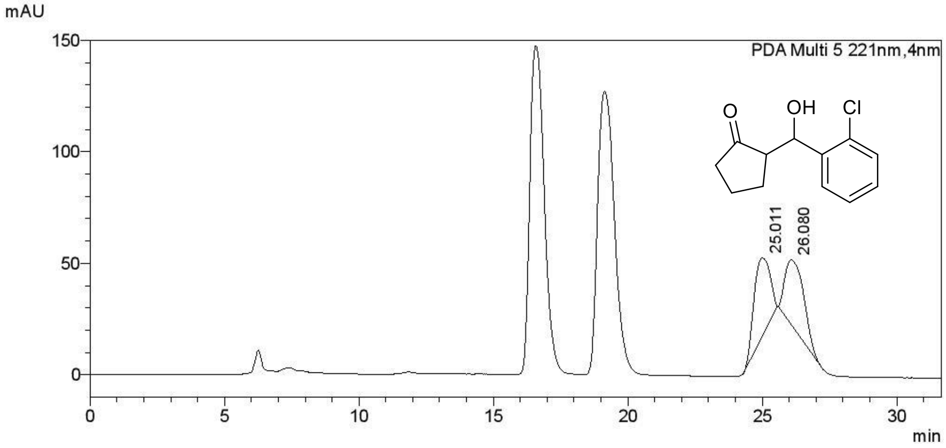

<Peak Table>

PDA Ch5 221nm

Peak#	Ret. Time	Area	Height	Area%
1	25.011	1482514	34889	50.467
2	26.080	1455104	29031	49.533
Total		2937618	63921	100.000

C:\Users\Shimadzu\Desktop\Hayley\cyclopentanone reaction\HC-I-37 Race 4%IPAnHex 20uL 0.5 mLmin 22Mar23.lcd

Figure S45. HPLC trace of racemic aldol product **4f** (above).

SHIMADZU LabSolutions

Analysis Report

<Sample Information>

Sample Name	: HC-I-31 4%IPAnHex 20uL 0.5 mLmin 23Mar23		
Sample ID	:		
Data Filename	: HC-I-31 4%IPAnHex 20uL 0.5 mLmin 23Mar23.lcd		
Method Filename	: trial.lcm		
Batch Filename	:		
Vial #	: 1-76	Sample Type	: Unknown
Injection Volume	: 20 uL		
Date Acquired	: 3/23/2023 4:34:32 PM	Acquired by	: System Administrator
Date Processed	: 3/23/2023 5:10:54 PM	Processed by	: System Administrator

<Chromatogram>

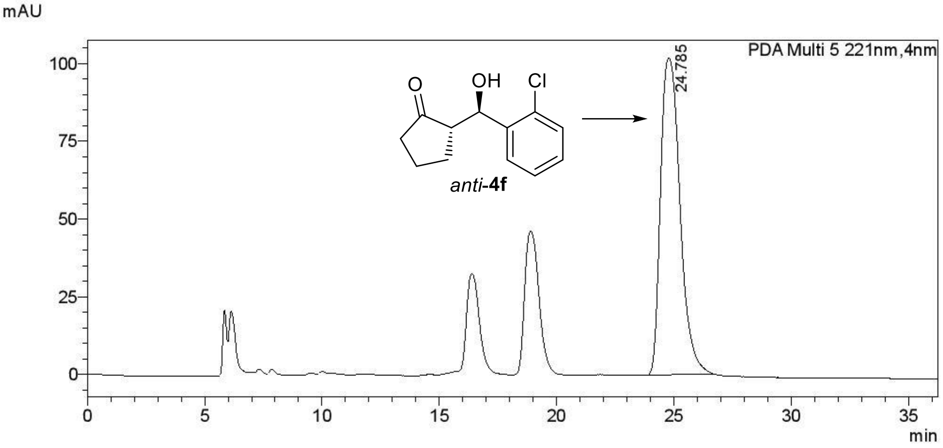

<Peak Table>

PDA Ch5 221nm

Peak#	Ret. Time	Area	Height	Area%
1	24.785	5862180	101873	100.000
Total		5862180	101873	100.000

Figure S46. HPLC trace of the enantioenriched *anti*-aldol (major) product **4f** (above).

Section S7. Table 3 entries and characterization of *anti*-aldol product **4g** and *syn*-aldol products **4h**, **i**, **j**

Table 3, entry 1: Competition reaction between 2,2-dimethyl-1,3-dioxan-5-one and acetylacetone for the limiting reagent 4-nitrobenzaldehyde

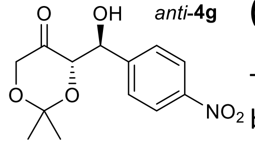
anti-**4g**

(*S*)-4-((*S*)-hydroxy(4-nitrophenyl)methyl)-2,2-dimethyl-1,3-dioxan-5-one

To a clean screw cap V-shaped reaction vessel (5 mL) equipped with a pyramidal stir bar were subsequently added 2,2-dimethyl-1,3-dioxan-5-one (MW = 130.14, 2.00 equiv, 2.00 mmol, density = 1.09 g/mL, 239 µL), acetylacetone (MW = 100.12, 2.00 equiv, 2.00 mmol, density = 0.975 g/mL, 205 µL), mortar and pestle ground 4-nitrobenzaldehyde (MW = 151.12, 1.0 equiv, 1.0 mmol, 151.12 mg), brine (MW = 18.02, 15 equiv, 15.0 mmol, density = 1.00 g/mL, 270 µL), and *trans*-4-(*tert*-butyldiphenylsilyloxy)-L-proline catalyst (MW = 369.54, 5.0 mol%, 0.05 mmol, 18.48 mg) in the stated order. The resulting heterogenous reaction mixture was light yellow and turned orange by the end of the 30 h reaction time. Stirring was performed such that the reaction did not splash against the vessel walls, by doing so reproducible yield data was achieved. Note that water provided lower *ee*, but that needs to be confirmed using an alternative chiral HPLC column because the two anti-aldol enantiomers are not base line resolved (**Figures S49** and **S50**).

This compound has been synthesized by many research groups, one example is:
Ramasastry, S. S. V.; Albertshofer, K.; Utsumi, N.; Tanaka, F.; Barbas III, C. F. Mimicking fructose and rhamnulose aldolases: organocatalytic syn-aldol reactions with unprotected dihydroxyacetone. *Angew. Chem. Int. Ed.* 2007, *46*, 5572-5575.

Crude product ^{1}H NMR analysis (**Figure S47**, see below) allowed the following diastereo- and chemoselectivity ratios to be determined.

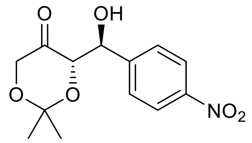
anti-aldol (major)

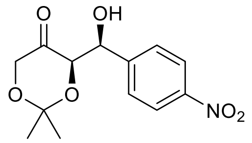
syn-aldol (minor)

Diastereoselectivity: The *anti*-aldol/*syn*-aldol product ratio was determined as 16.7:1 based on the *anti*-aldol product resonance at 5.00 ppm (d, representing the benzylic proton) and the *syn*-aldol product resonance at 5.32 ppm (d, representing the benzylic proton). For literature regarding the *syn*-aldol product chemical shift, see page S9, product **18** within the Supporting Information of: S. Ramasastry, K. Albertshofer, N. Utsumi, F. Tanaka, and C. Barbas, *Angew. Chem. Int. Ed.* **2007**, *46*, 5572-5575.

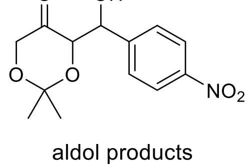
aldol products

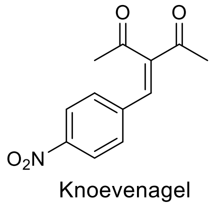
Knoevenagel product

Chemoselectivity: The aldol/Knoevenagel product ratio was determined to be 6.8:1 based on the *anti*-aldol product resonance at 5.00 ppm (d representing the benzylic proton) and the *syn*-aldol product resonance at 5.32 ppm (d, representing the benzylic proton) *versus* the Knoevenagel condensation product resonance at 2.27 ppm (s, representing the methyl group). For literature regarding the Knoevenagel condensation product chemical shift, see page 2258, product **3g**, of Y. Zhang, C. Sun, J. Liang, and Z. Shang, *Chin. J. Chem.* **2010**, *28*, 2255—2259.

Purification and yield: Silica gel chromatography (35 mm column outer diameter, 16 cm silica bed height) was performed using gradient elution (0.25-0.75 vol% acetone in CH_2Cl_2). The crude product was always loaded onto the column in a minimum volume of CH_2Cl_2. The *anti*-aldol product **4g** was isolated as a single diastereomer as a white solid (177.2 mg, MW = 281.26, 0.63 mmol, 63% yield). A 58% yield was obtained for a reaction under the same conditions but using 3 equiv deoxygenated H_2O instead of 15 equiv of brine.

TLC: R_f = 0.3 acetone/CH_2Cl_2 (0.5:99.5)

95% *ee*: Chiralpak OD-H chiral HPLC column, n-hexane/iPrOH (95:5), flow rate = 1.0 mL/min, λ = 254 nm, *anti*-aldol product t_{major}= 17.7 min, t_{minor}= 20.1 min retention times were observed (**Figure S50**).

^{1}H NMR (400 MHz, $CDCl_3$) [ppm] *anti*-aldol (major) product **4g**: δ 8.21 (d, 2H, J = 8.9 Hz), 7.59 (d, 2H, J = 8.8 Hz), 5.00 (dd, 1H, J = 7.9, 2.6 Hz), 4.29 (dd, 1H, J = 17.7, 1.5 Hz), 4.21 (dd, 1H, J = 8.0, 1.5 Hz), 4.08 (d, 1H, J = 17.7 Hz), 3.81 (d, 1H, J = 2.7 Hz), 1.39 (s, 3H), 1.21 (s, 3H).

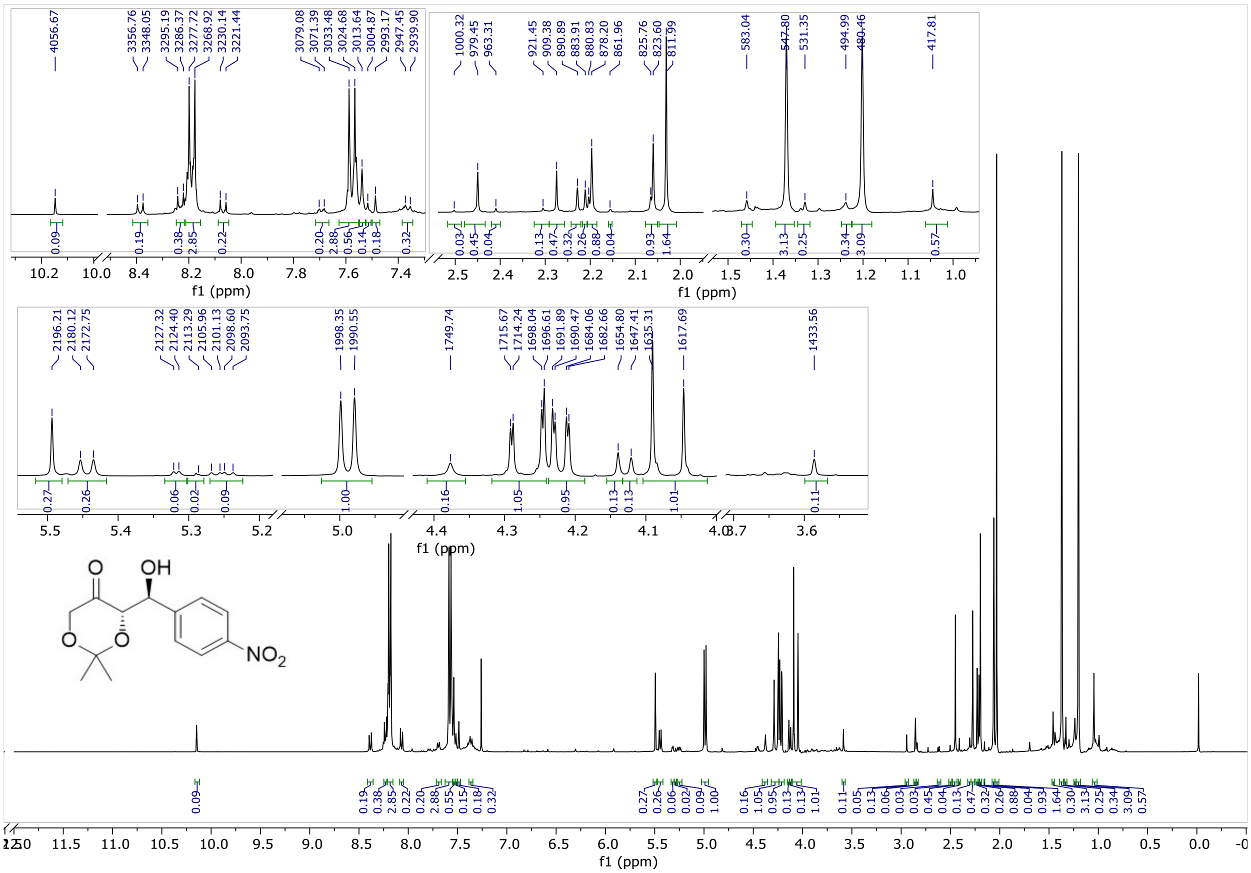

Figure S47. Crude ^{1}H NMR spectrum after high vacuum drying overnight (above).

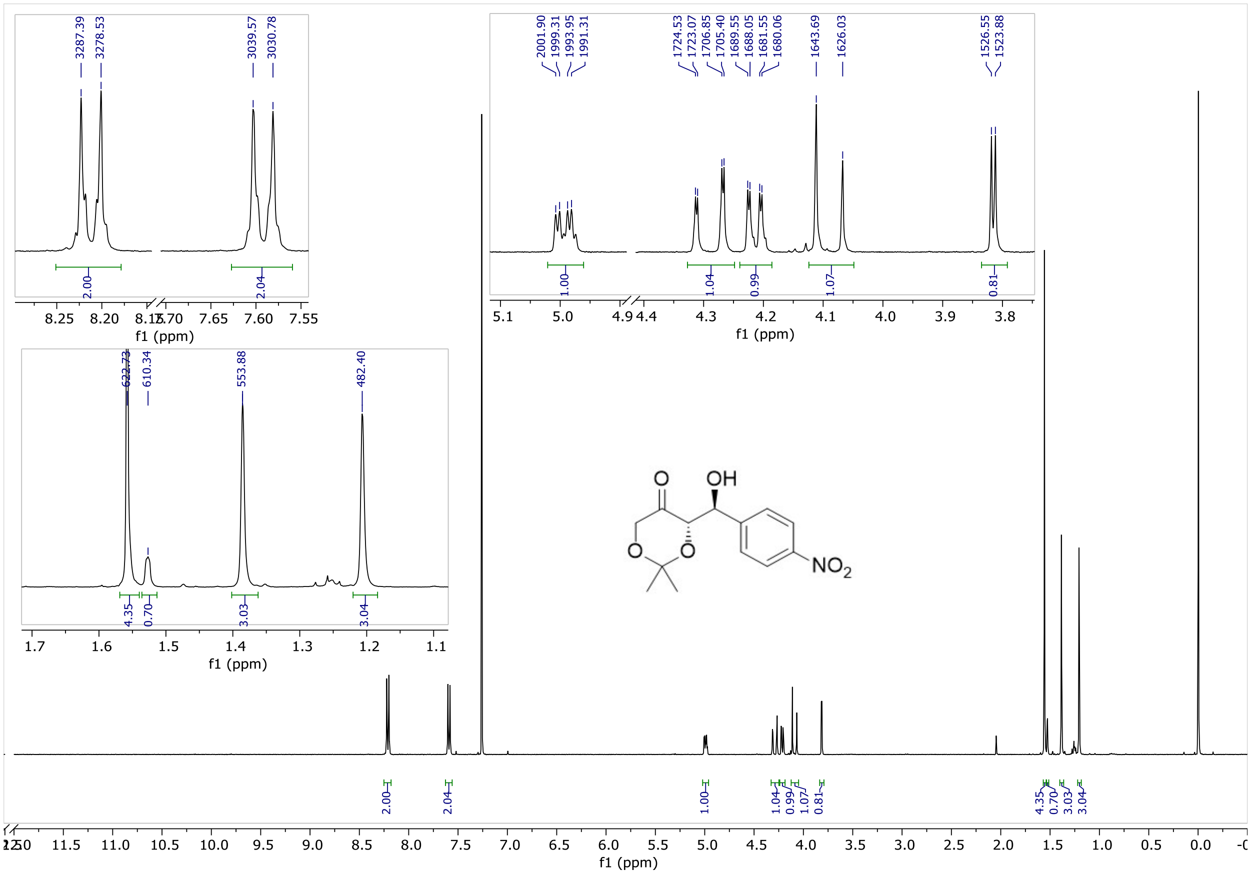

Figure S48. ^{1}H NMR spectrum of the purified *anti*-aldol product **4g** (above).

SHIMADZU LabSolutions

Analysis Report

<Sample Information>

Sample Name	: SSZ33-RACE 5%IPAnHex 1mLmin 1mgmL 10Aug23		
Sample ID	:		
Data Filename	: SSZ33-RACE 5%IPAnHex 1mLmin 1mgmL 20 uL 10Aug23.lcd		
Method Filename	: trial.lcm		
Batch Filename	:		
Vial #	: 1-1	Sample Type	: Unknown
Injection Volume	: 20 uL		
Date Acquired	: 8/10/2023 3:25:07 PM	Acquired by	: System Administrator
Date Processed	: 8/14/2023 4:35:17 PM	Processed by	: System Administrator

<Chromatogram>

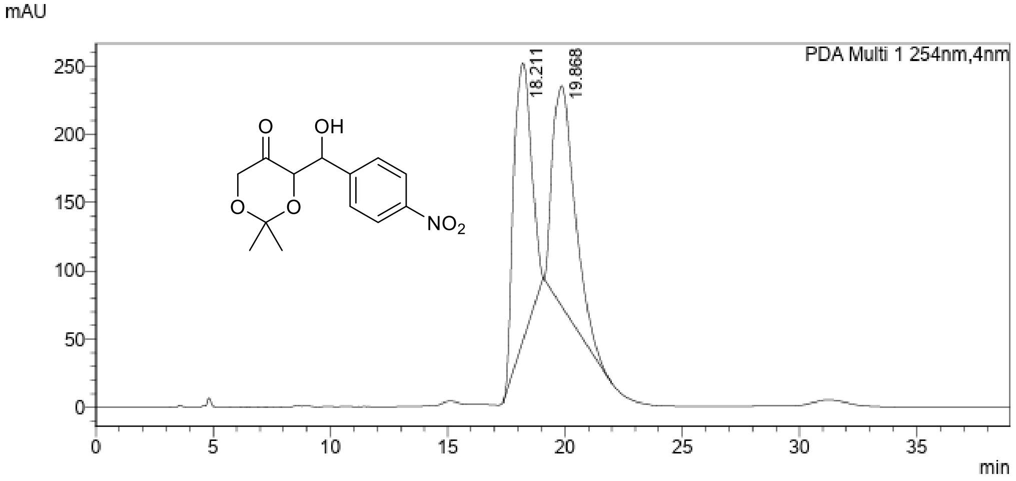

<Peak Table>

PDA Ch1 254nm

Peak#	Ret. Time	Area	Height	Area%
1	18.211	10783191	203257	50.099
2	19.868	10740403	161614	49.901
Total		21523593	364871	100.000

Figure S49. HPLC trace of racemic aldol product **4g** (above).

Analysis Report

<Sample Information>

Sample Name : SSZ42 5%IPAnHex 1mLmin 1mgmL 20uL 17Aug23
Sample ID :
Data Filename : SSZ42 5%IPAnHex 1mLmin 1mgmL 20uL 17Aug23.lcd
Method Filename : trial.lcm
Batch Filename :
Vial # : 1-1 Sample Type : Unknown
Injection Volume : 20 uL
Date Acquired : 8/17/2023 3:34:39 PM Acquired by : System Administrator
Date Processed : 8/18/2023 12:07:34 PM Processed by : System Administrator

<Chromatogram>

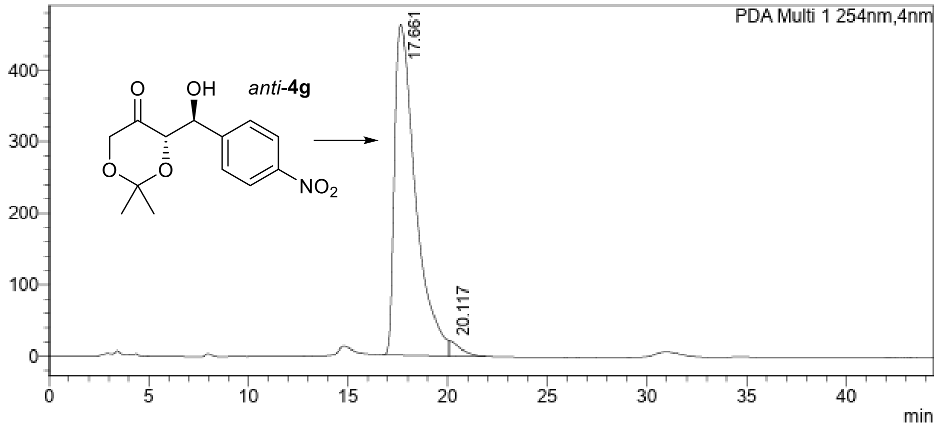

<Peak Table>

PDA Ch1 254nm

Peak#	Ret. Time	Area	Height	Area%
1	17.661	33660309	462212	97.690
2	20.117	795956	20824	2.310
Total		34456265	483036	100.000

Figure S50. HPLC trace of the enantioenriched *anti*-aldol product **4g** (above).

Table 3, entry 2: Competition reaction between 1-(*t*-butyldimethylsilyloxy)-2-propanone and acetylacetone for the limiting reactant 4-nitrobenzaldehyde

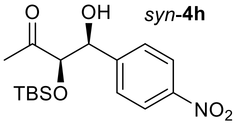

syn-**4h**

(3*R*,4*S*)-3-((*tert*-butyldimethylsilyl)oxy)-4-hydroxy-4-(4-nitrophenyl)butan-2-one

To a clean screw cap V-shaped reaction vessel (5.0 mL) equipped with a pyramidal stir bar, were added 1-(*t*-butyldimethylsilyloxy)-2-propanone (MW= 188.34, 1.5 equiv, 2.25 mmol, density= 0.976 g/mL, 434 µL), acetylacetone (MW= 100.12, 1.50 equiv, 2.25 mmol, density= 0.975 g/mL, 231 µL), O-tBu-L-threonine (MW= 175.23, 15 mol%, 0.225 mmol, 39.4 mg,) and mortar and pestle ground 4-nitrobenzaldehyde (MW= 151.12, 1.00 equiv, 1.50 mmol, 227 mg). Within a minute of adding 4-nitrobenzaldehyde, water (MW= 18.02, 7.5 equiv, 11.25 mmol, 203 µL) was added. The resulting heterogenous solution was rigorously stirred for 30 h such that an emulsion was always noted, albeit without causing splashing against the vessel walls, by doing so reproducible yield data was achieved. Screening reactions revealed that 15 equiv of water reduced the chemoselectivity.

This compound has been previously synthesized, see for example:
1. Utsumi, N.; Imai, M.; Ramasastry, S. S. V.; Barbas, C. F. III. Mimicking aldolases through organocatalysis: syn-selective aldol reactions with protected dihydroxyacetone. *Org. Lett.* **2007**, *9*, 3445-3448.
2. Kumar, A.; Singh, S.; Kumar, V.; Chimni, S. S. Asymmetric syn-selective direct aldol reaction of protected hydroxyacetone catalyzed by primary amino acid derived bifunctional organocatalyst in the presence of water. *Org. Biomol. Chem*. **2011**, *9*, 2731-2742.
3. Hikawa, R.; Shimogaki, M.; Kano, T. Design of threonine-derived amino sulfonamide organocatalysts for the Highly Stereoselective Aldol Reactions. *Asian J. Org. Chem.* **2023**, e202300113.

Crude product ^{1}H NMR analysis (**Figure S51**, see below) allowed the following diastereo- and chemoselectivity ratios to be determined.

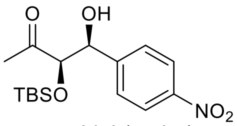

syn-aldol (major)

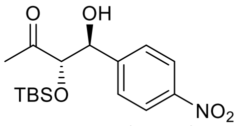

anti-aldol (minor)

Diastereoselectivity: The *syn*/*anti* ratio was determined as 9.1:1, by comparing the *syn*-aldol resonance at 5.02 ppm (bs, representing the benzylic proton) and the *anti*-aldol resonance at 4.91 ppm (d, representing the benzylic proton). For literature regarding the *anti*-aldol product chemical shifts, see page 2738 within A. Kumar, S. Singh, V. Kumar, S. S. Chimni, *Org. Biomol. Chem*. **2011**, *9*, 2731-2742.

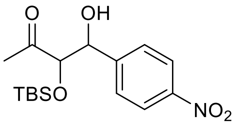

aldol products

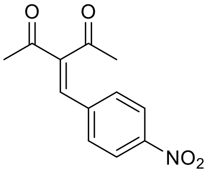

Knoevenagel product

Chemoselectivity: The aldol/Knoevenagel product ratio was determined to be 15.9:1, based on the addition of the *syn*-aldol product resonance at 5.02 ppm (bs, representing the benzylic proton) and the *anti*-aldol resonance at 4.91 ppm (d, representing the benzylic proton) *versus* the Knoevenagel product resonance at 2.28 ppm (s, representing one of the methyl groups). For the literature regarding the Knoevenagel condensation product chemical shift, see page 2258 within Y. Zhang, C. Sun, J. Liang, Z. Shang, *Chin. J. Chem*. **2010**, *28*, 2255-2259.

Purification and yield: Silica gel chromatography (3.5 cm column diameter, 16 cm silica gel height) was performed by adding the crude product dissolved in a minimum volume of CH_2Cl_2. Gradient elution was

used (15 to 20% ethyl acetate in petroleum ether). The *syn*- and *anti*-aldol products **4h** were isolated in pure form as a white solid (408.9 mg, MW= 339.46, 1.20 mmol, 80% yield).

TLC: R_f = 0.30, EtOAc/petroleum ether (1:4)

97% *ee*: Chiralpak OD-H HPLC column, n-hexane/iPrOH (95:5), flow rate= 1.0 mL/min, λ = 254 nm (and variations thereof) did not result in sufficient baseline separation of the *syn*(major)-aldol and *anti*(minor)-aldol products **4h**, see HPLC chromatograms for the racemic and enantioenriched products in **Figures S53** and **S54** (below). However, the *ee* of the acetate of the *syn*-aldol could be determined (**Figures S56** and **S57**, see respectively six and seven pages forward). For the acetylation procedure, ^{1}H NMR data and HPLC chromatograms, see four pages forward.

^{1}H NMR (400 MHz, $CDCl_3$) (ppm) *syn*-aldol (major) product: δ -0.36 (s, 3H), -0.03 (s, 3H), 0.86 (s, 9H), 2.21 (s, 3H), 3.14 (d, 1H, OH, J= 8.92 Hz), 4.17 (d, 1H, J= 2.86 Hz), 5.02 (dd, 1H, J= 2.75, 9.03 Hz), 7.52-7.54 (d, 2H, J= 9.03 Hz), 8.20-8.24 (d, 2H, J= 8.9 Hz).

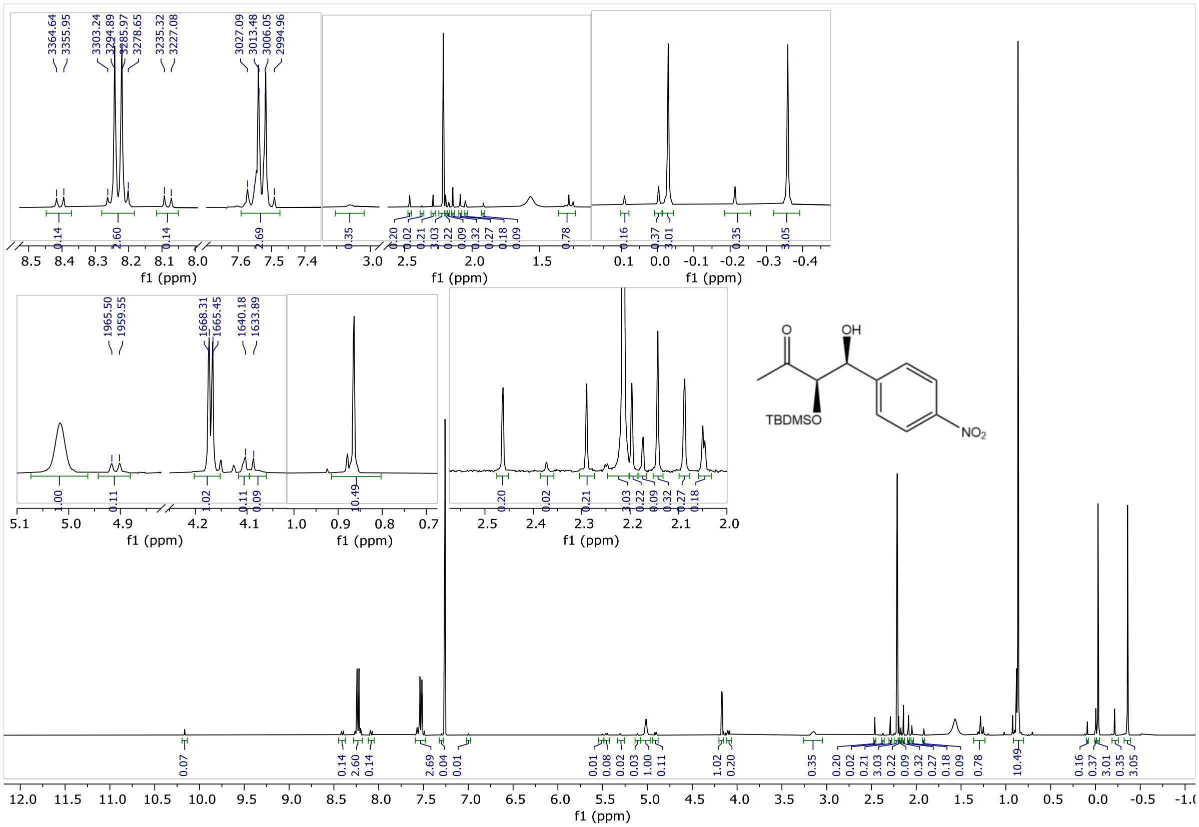

Figure S51. Crude ^{1}H NMR spectrum after high vacuum drying overnight (above).

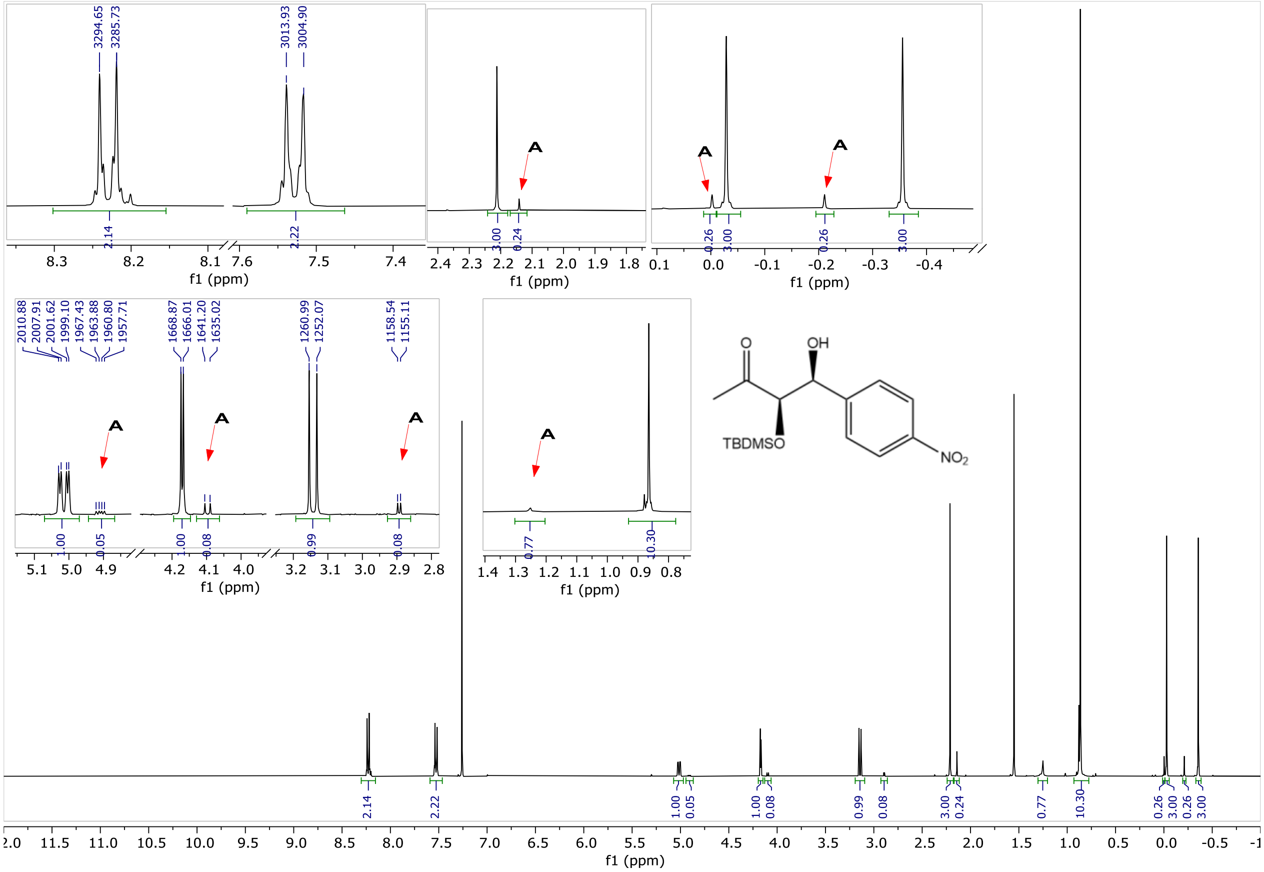

Figure S52. ^{1}H NMR spectrum of the purified *syn*(major)- and *anti*(minor)-aldol products (above). We followed literature precedent and isolated both the *syn*- and *anti*-aldol products together for the yield determination. Note the red arrows in the expansions show the minor *anti*-aldol product (A). For literature regarding the *anti*-aldol product chemical shift, see page 2738 within: A. Kumar, S. Singh, V. Kumar, S. S. Chimni, *Org. Biomol. Chem*. **2011**, *9*, 2731-2742.

SHIMADZU LabSolutions

Analysis Report

<Sample Information>

Sample Name	: NK36 racemate 5%IPAnHex 20uL 1.0 mLmin 03Feb23		
Sample ID	:		
Data Filename	: NK36 racemate2 5%IPAnHex 20uL 1.0 mLmin 03Feb23.lcd		
Method Filename	: trial.lcm		
Batch Filename	:		
Vial #	: 1-3	Sample Type	: Unknown
Injection Volume	: 20 uL		
Date Acquired	: 2/3/2023 5:00:26 PM	Acquired by	: System Administrator
Date Processed	: 2/3/2023 5:21:38 PM	Processed by	: System Administrator

<Chromatogram>

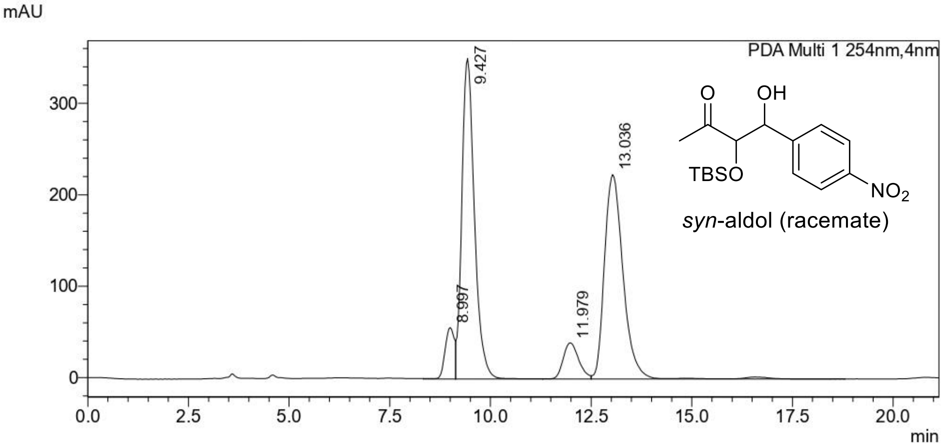

<Peak Table>

PDA Ch1 254nm

Peak#	Ret. Time	Area	Height	Area%
1	8.997	931449	56287	5.515
2	9.427	7625877	350435	45.155
3	11.979	1067583	39602	6.322
4	13.036	7263216	223430	43.008
Total		16888124	669754	100.000

C:\Users\Shimadzu\Desktop\Ninos\Runs\NK36 racemate2 5%IPAnHex 20uL 1.0 mLmin 03Feb23.lcd

Figure S53. HPLC trace of racemic *syn*(major)-aldol product **4h** (above).

SHIMADZU LabSolutions

Analysis Report

<Sample Information>

Sample Name	: NK35 product 5%IPAnHex 20uL 1.0 mLmin 03Feb23		
Sample ID	:		
Data Filename	: NK35 product 5%IPAnHex 20uL 1.0 mLmin 03Feb23.lcd		
Method Filename	: trial.lcm		
Batch Filename	:		
Vial #	: 1-4	Sample Type	: Unknown
Injection Volume	: 20 uL		
Date Acquired	: 2/3/2023 5:30:10 PM	Acquired by	: System Administrator
Date Processed	: 2/3/2023 5:51:40 PM	Processed by	: System Administrator

<Chromatogram>

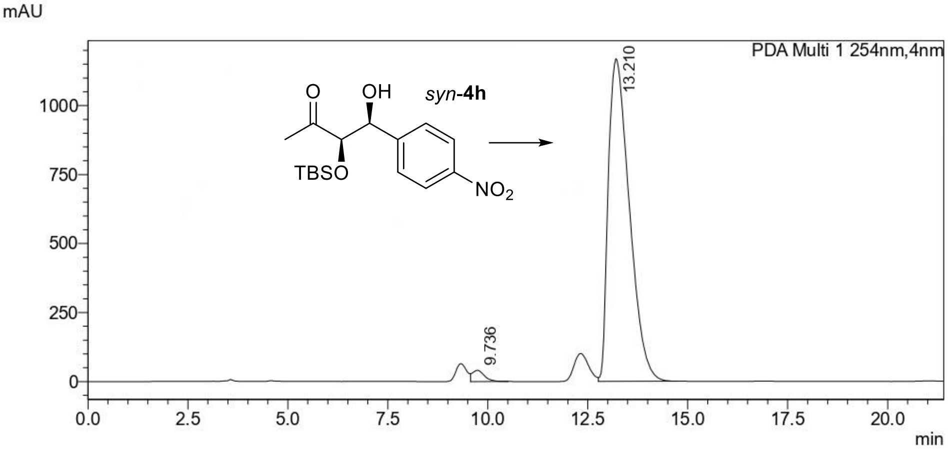

<Peak Table>

PDA Ch1 254nm

Peak#	Ret. Time	Area	Height	Area%
1	9.736	835371	40572	1.914
2	13.210	42816720	1169247	98.086
Total		43652091	1209818	100.000

C:\Users\Shimadzu\Desktop\Ninos\Runs\NK35 product 5%IPAnHex 20uL 1.0 mLmin 03Feb23.lcd

Figure S54. HPLC trace of the enantioenriched *syn*(major)-aldol product **4h** (above).

Acetylation of (3*R*,4*S*)-3-((*tert*-butyldimethylsilyl)oxy)-4-hydroxy-4-(4-nitrophenyl) butan-2-one (4h)

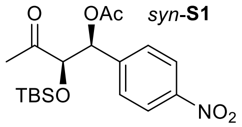

syn-**S1**

(1*S*,2*R*)-2-((*tert*-butyldimethylsilyl)oxy)-1-(4-nitrophenyl)-3-oxobutyl acetate (S1)

To a clean round bottom flask (50 mL) equipped with a magnetic stir bar, were added (3*R*,4*S*)-3-((*tert*-butyldimethylsilyl)oxy)-4-hydroxy-4-(4-nitrophenyl)butan-2-one (**4h**) (MW= 339.15, 1.0 equiv, 0.66 mmol, 224 mg), 4-dimethylaminopyridine (MW= 122.17, 1.2 equiv, 0.792 mmol, 96.8 mg). The round bottom flask was evacuated and flooded with nitrogen. Anhydrous dichloromethane (MW= 84.93, 0.09 M, density = 1.325 g/mL, 7.5 mL) was added, followed by triethylamine (MW= 101.19, 6.0 equiv, 3.96 mmol, density = 0.726 g/mL, 0.551 mL). Finally, acetic anhydride (MW= 102.09, 5.0 equiv, 3.3 mmol, density = 1.08 g/mL, 0.311 mL) was added dropwise over thirty seconds. The resulting solution was rigorously stirred for 36 h, but we inform the experimentalist that later experience informs us that 1-2 h is sufficient.

Work up: To a separatory funnel was added aqueous 0.5 N HCl (30 mL) and the reaction contents were then transferred to the separatory funnel. The round bottom flask was rinsed with CH_2Cl_2 (3 x 10 mL) and added to the separatory funnel. The CH_2Cl_2 layer was collected, and the aqueous solution was further extracted with CH_2Cl_2 (4 x 30.0 mL). The combined organic layers were dried over Na_2SO_4, filtered, and concentrated under rotary evaporation. The crude product was obtained after drying on high vacuum overnight.

Purification: Silica gel chromatography (3.5 cm column diameter, 16 cm silica gel height) was performed by adding the crude product dissolved in a minimum volume of CH_2Cl_2. Gradient elution was used (10 to 15% ethyl acetate in petroleum ether) and enabled isolation of the acetylated *syn*-product **S1** in pure form as a white solid. Note by TLC analysis this is a one spot to one spot reaction, unfortunately we did not record the yield but expect it to be quantitative.

TLC: R_f = 0.31, EtOAc/petroleum ether (15:85)

97% *ee*: Chiralpak OD-H, n-hexane/iPrOH (95:5), flow rate= 1.0 mL/min, λ = 254 nm, major *syn*-aldol product t_{minor} = 7.8 min, t_{major}= 8.6 min (**Figure S57**).

^{1}H NMR (400 MHz, $CDCl_3$) (ppm) acetylated *syn*-aldol product **4h**: δ -0.35 (s, 3H), -0.06 (s, 3H), 0.88 (s, 9H), 2.14 (s, 3H), 2.17 (s, 3H), 4.21 (d, 1H, J= 4.0 Hz), 5.97 (d, 1H, J= 4.01 Hz), 7.49-7.51 (d, 2H, J= 8.3 Hz), 8.20-8.23 (d, 2H, J= 8.9 Hz).

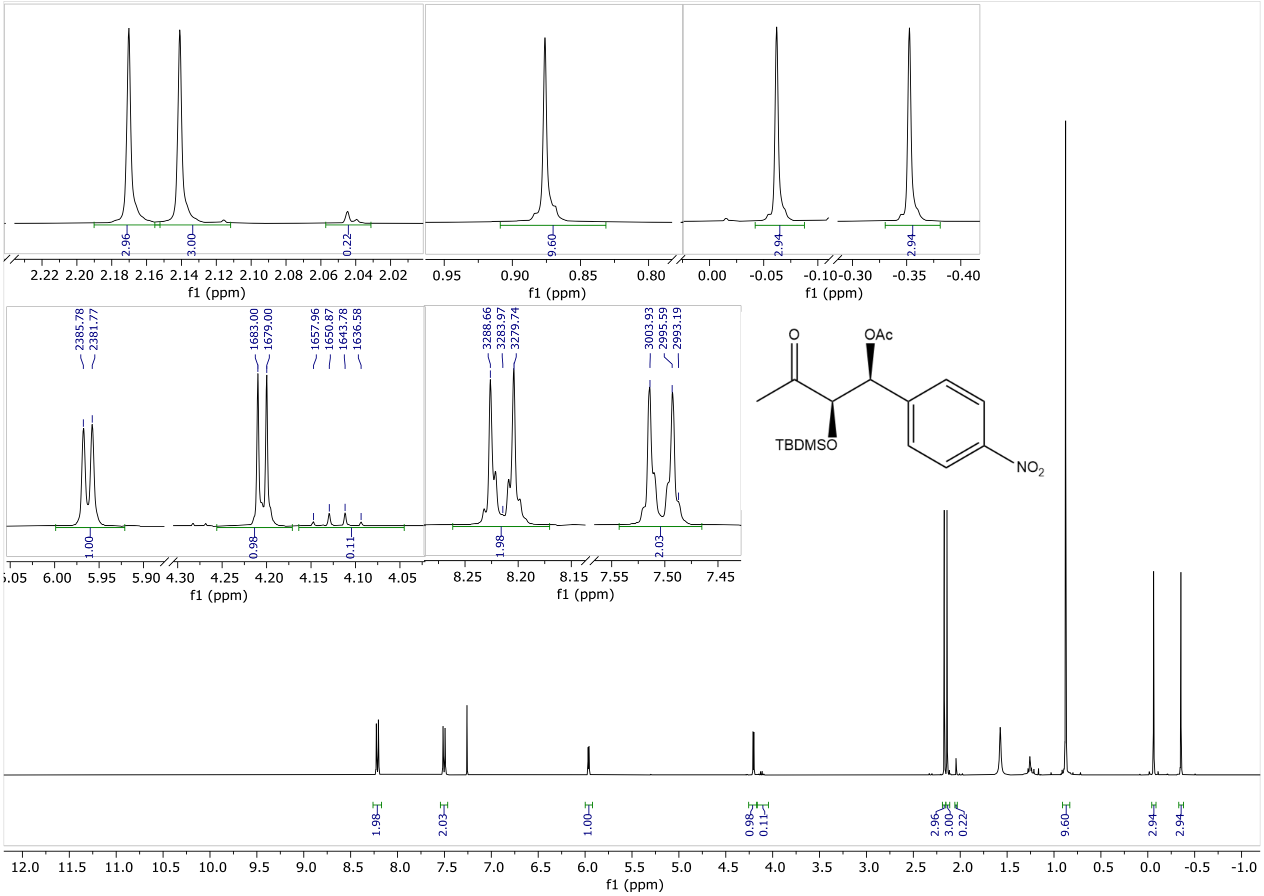

Figure S55. ^{1}H NMR spectrum of the purified acetylated *syn*-aldol product **S1** (above). For the literature regarding this acetylated *syn*-aldol product, see page 11 within the SI of N. Utsumi, M. Imai, F. Tanaka, S. S. V. Ramasastry, C. F. Barbas III, *Org. Lett.* **2007**, *9*, 3445–3448.

SHIMADZU LabSolutions

Analysis Report

<Sample Information>

Sample Name : NK53 5%IPAnHex 10uL 1.0 mLmin 9Apr23
Sample ID :
Data Filename : NK53 5%IPAnHex 10uL 1.0 mLmin 9Apr23.lcd
Method Filename : trial.lcm
Batch Filename :
Vial # : 1-6   Sample Type : Unknown
Injection Volume : 10 uL
Date Acquired : 4/9/2023 4:13:15 PM   Acquired by : System Administrator
Date Processed : 4/9/2023 4:28:13 PM   Processed by : System Administrator

<Chromatogram>

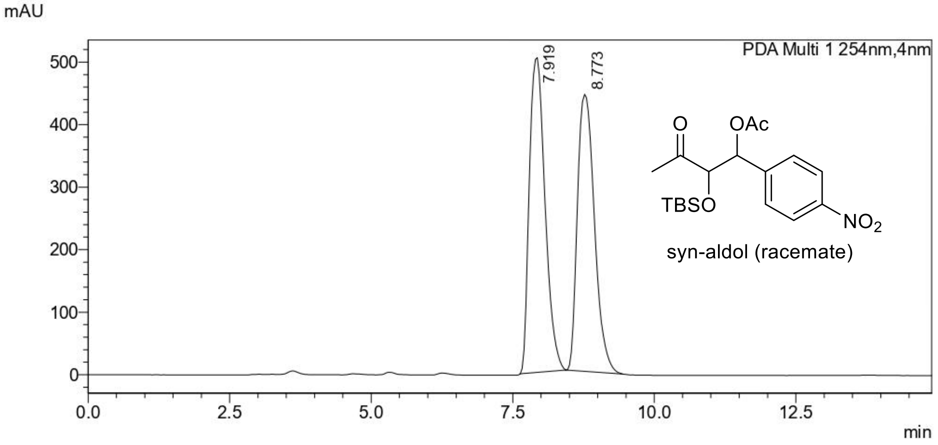

<Peak Table>

PDA Ch1 254nm

Peak#	Ret. Time	Area	Height	Area%
1	7.919	9735419	503466	51.210
2	8.773	9275275	443346	48.790
Total		19010694	946811	100.000

C:\Users\Shimadzu\Desktop\Ninos\Runs\NK53 5%IPAnHex 10uL 1.0 mLmin 9Apr23.lcd

Figure S56. HPLC trace of racemic acetylated *syn*-aldol product **S1** (above).

SHIMADZU LabSolutions

Analysis Report

<Sample Information>

Sample Name	: NK49 5%IPAnHex 10uL 1.0 mLmin 24Mar23
Sample ID	:
Data Filename	: NK49 5%IPAnHex 10uL 1.0 mLmin 24Mar23.lcd
Method Filename	: trial.lcm
Batch Filename	:
Vial #	: 1-4
Injection Volume	: 10 uL
Date Acquired	: 3/24/2023 4:03:01 PM
Date Processed	: 3/24/2023 4:20:25 PM

Sample Type	: Unknown
Acquired by	: System Administrator
Processed by	: System Administrator

<Chromatogram>

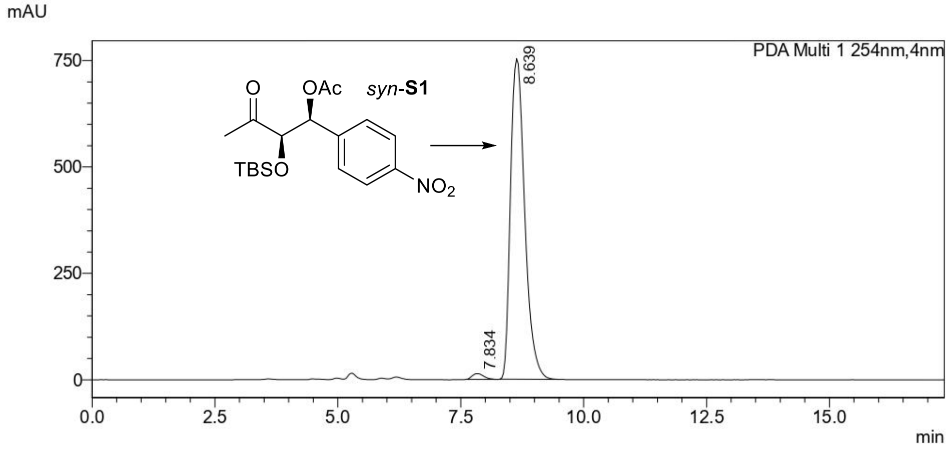

<Peak Table>

PDA Ch1 254nm

Peak#	Ret. Time	Area	Height	Area%
1	7.834	233021	13733	1.500
2	8.639	15302890	753274	98.500
Total		15535911	767007	100.000

C:\Users\Shimadzu\Desktop\Ninos\Runs\NK49 5%IPAnHex 10uL 1.0 mLmin 24Mar23.lcd

Figure S57. HPLC trace of the enantioenriched acetylated *syn*-aldol product **S1** (above).

Table 3, entry 3: Competition reaction between 1-(*t*-butydimethylsilyloxy)-2-propanone and acetylacetone for the limiting reactant 4-cyanobenzaldehyde

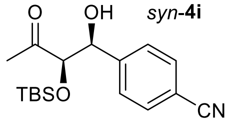

syn-**4i**

4-((1*S*,2*R*)-2-((*tert*-butyldimethylsilyl)oxy)-1-hydroxy-3-oxobutyl)benzonitrile

To a clean screw cap V-shaped reaction vessel (5.0 mL) equipped with a pyramidal stir bar, were added 1-(*t*-butyldimethylsilyloxy)-2-propanone (MW= 188.34, 1.50 equiv, 2.25 mmol, density= 0.976 g/mL, 434 μL), acetylacetone (MW= 100.12, 1.50 equiv, 2.25 mmol, density= 0.975 g/mL, 231 μL), O-tBu-L-threonine (MW= 175.23, 15 mol%, 0.225 mmol, 39.4 mg,) and mortar and pestle ground 4-cyanobenzaldehyde (MW= 131.13, 1.0 equiv, 1.5 mmol, 196.69 mg). Within a minute of adding 4-cyanobenzaldehyde, water (MW= 18.02, 15.0 equiv, 22.5 mmol, 405.45 μL) was added. The resulting heterogenous solution was rigorously stirred for 40 h such that an emulsion was always noted, albeit without causing splashing against the vessel walls; by doing so reproducible yield data was achieved.

This compound has been previously synthesized, see for example:
1. Kumar, A.; Singh, S.; Kumar, V.; Chimni, S. S. Asymmetric syn-selective direct aldol reaction of protected hydroxyacetone catalyzed by primary amino acid derived bifunctional organocatalyst in the presence of water. *Org. Biomol. Chem.* **2011**, *9*, 2731-2742.
2. Wu, X.; Jiang, Z.; Shen, H-M.; Lu, Y. Highly efficient threonine-derived organocatalysts for direct asymmetric aldol reactions in water. *Adv. Synth. Catal.* **2007**, *349*, 812-816.

Crude product ^{1}H NMR analysis (**Figure S58**, see below) allowed the following diastereo- and chemoselectivity ratios to be determined.

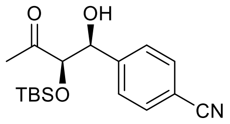

syn-aldol (major)

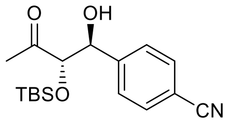

anti-aldol (minor)

Diastereoselectivity: The *syn*/*anti* ratio was determined as 8.3:1, by comparing the *syn*-aldol resonance at 4.97 ppm (bs, representing the benzylic proton) and the *anti*-aldol resonance at 4.84 ppm (d, representing the benzylic proton). For literature regarding the *anti*-aldol product chemical shift, please see page 2739 within A. Kumar, S. Singh, V. Kumar, S. S. Chimni, *Org. Biomol. Chem*. **2011**, *9*, 2731-2742.

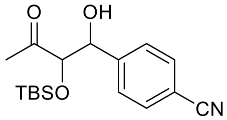

aldol products

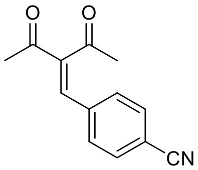

Knoevenagel product

Chemoselectivity: The aldol/Knoevenagel product ratio was determined to be 8.0:1, based on the addition of the *syn*-aldol product resonance at 4.97 ppm (bs, representing the benzylic proton) and the *anti*-aldol resonance at 4.84 ppm (d, representing the benzylic proton) *versus* the Knoevenagel product resonance at 2.28 ppm (the singlet representing one of the methyl groups). For literature regarding the Knoevenagel condensation product chemical shift, see page 2258 within Y. Zhang, C. Sun, J. Liang, Z. Shang, *Chin. J. Chem*. **2010**, *28*, 2255-2259.

Purification and yield: Silica gel chromatography (3.5 cm column diameter, 16 cm silica gel height) was performed by adding the crude product dissolved in a minimum volume of CH_2Cl_2. Gradient elution was used (10 to 20% ethyl acetate in petroleum ether). The *syn*- and *anti*-aldol products **4i** were isolated in pure form as a white solid (367.5 mg, MW= 319.48, 1.15 mmol, 77% yield).

TLC: R_f = 0.29, EtOAc/petroleum ether (1:4)

95% *ee*: Chiralpak OD-H HPLC column, n-hexane/iPrOH (95:5), flow rate= 1.0 mL/min, λ = 254 nm (and variations thereof) did not result in sufficient baseline separation of the *syn*(major)-aldol and *anti*(minor)-aldol products **4i**, see HPLC chromatograms for the racemic and enantioenriched products in **Figures S60** and **S61** (below). However, the *ee* of the acetylated derivative of the aldol product could be readily determined (**Figures S63** and **S64**, see respectively six and seven pages forward). For the acetylation procedure, ^{1}H NMR data and HPLC chromatograms, see four pages forward.

^{1}H NMR (400 MHz, $CDCl_3$) (ppm) *syn*(major)-aldol product **4i**: δ -0.37 (s, 3H), -0.04 (s, 3H), 0.85 (s, 9H), 2.21 (s, 3H), 3.06 (d, 1H, J= 8.71 Hz), 4.13 (d, 1H, J= 2.98 Hz), 4.97 (dd, 1H, J= 2.87, 8.82 Hz), 7.44-7.50 (d, 2H, J = 8.03 Hz), 7.63-7.70 (d, 2H, J = 8.58 Hz).

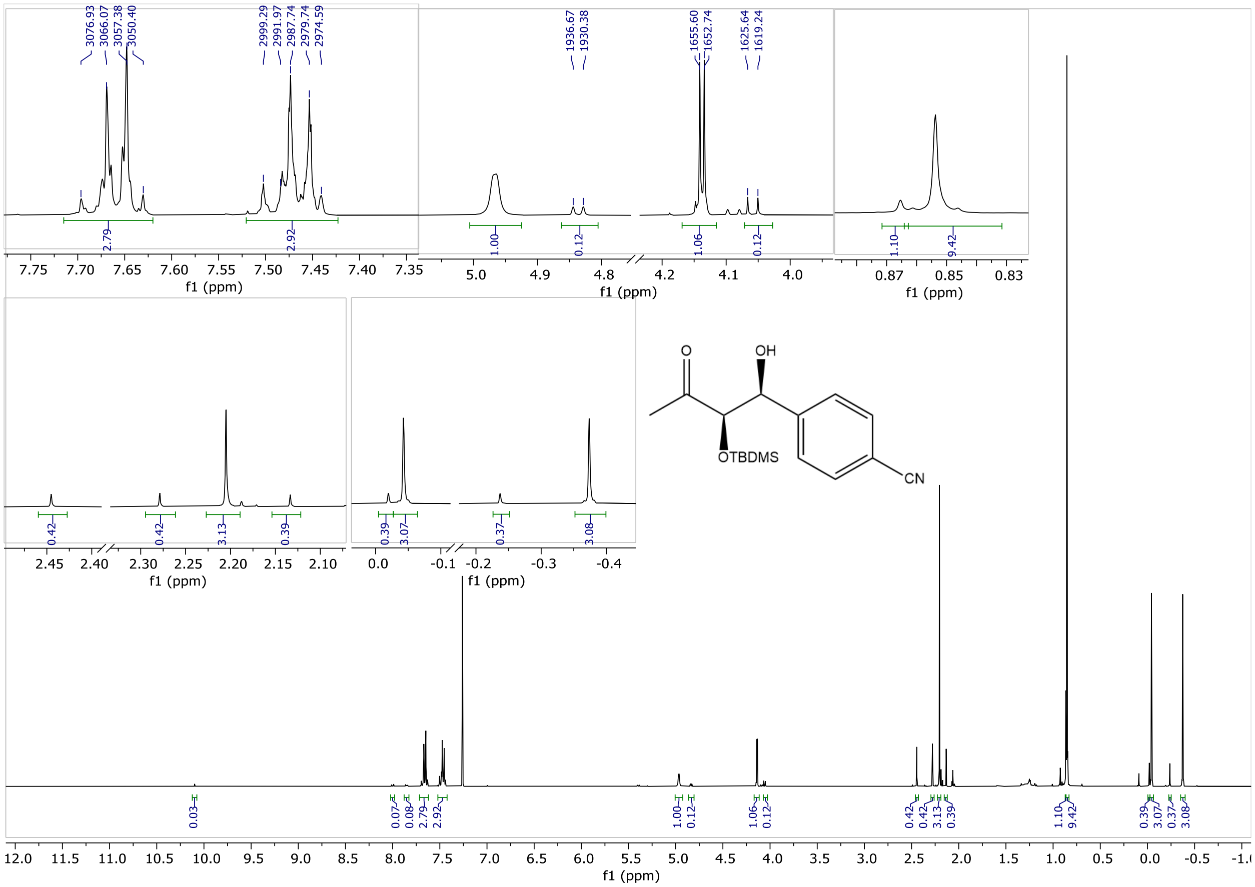

Figure S58. Crude ^{1}H NMR spectrum after high vacuum drying overnight (above).

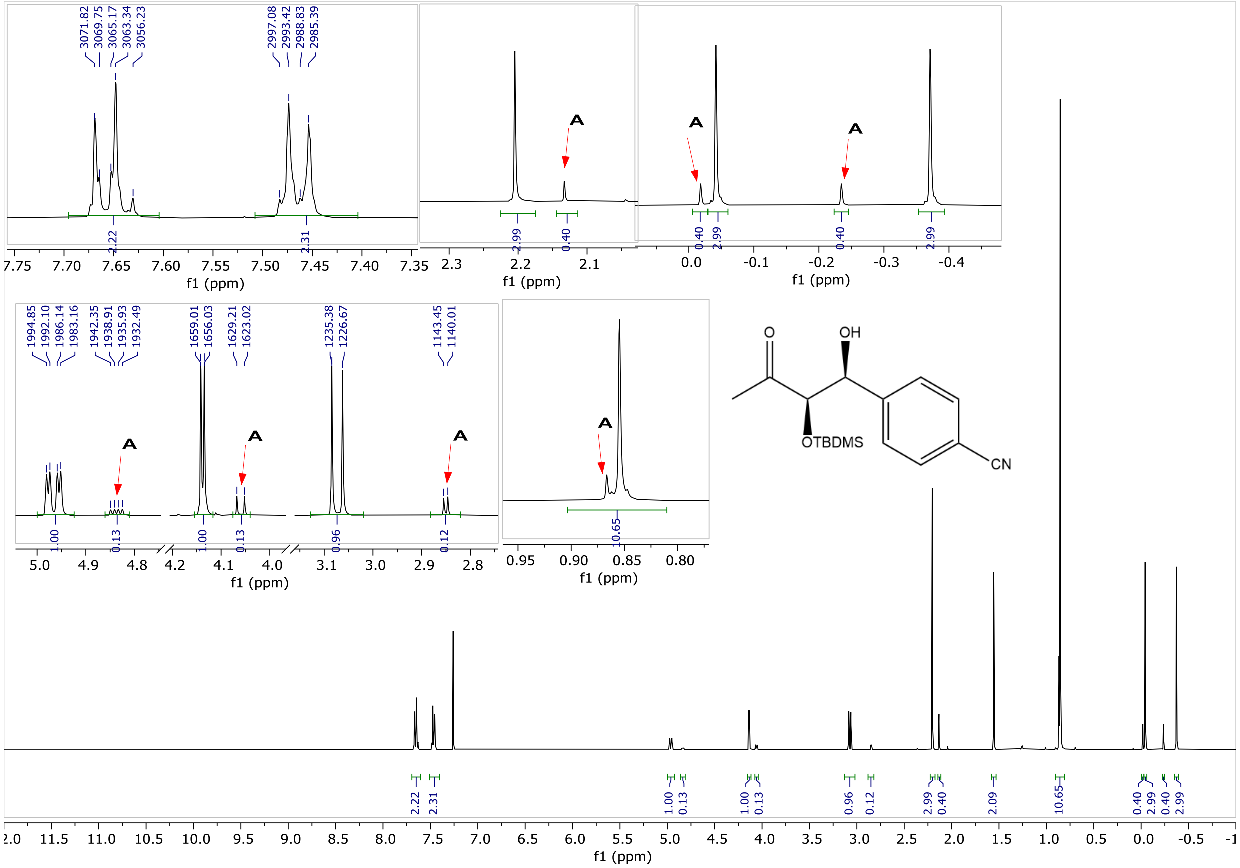

Figure S59. ^{1}H NMR spectrum of the purified *syn*-(major) and *anti*-(minor) aldol products (above). We followed literature precedent and isolated both the *syn*- and *anti*-aldol products together for the yield determination. Note the red arrows in the expansions show the minor *anti*-aldol product (A). For literature regarding the *anti*-aldol product chemical shift, see page 2739 within: A. Kumar, S. Singh, V. Kumar, S. S. Chimni, *Org. Biomol. Chem*. **2011**, *9*, 2731-2742.

SHIMADZU LabSolutions **Analysis Report**

<Sample Information>

Sample Name	: NK43 run race 5%IPAnHex 20uL 0.5 mLmin 20Feb23 1mgml .lcd		
Sample ID	:		
Data Filename	: NK43 run race 5%IPAnHex 20uL 0.5 mLmin 20Feb23 1mgml .lcd		
Method Filename	: trial.lcm		
Batch Filename	:		
Vial #	: 1-6	Sample Type	: Unknown
Injection Volume	: 20 uL		
Date Acquired	: 2/20/2023 12:39:45 PM	Acquired by	: System Administrator
Date Processed	: 2/20/2023 1:17:12 PM	Processed by	: System Administrator

<Chromatogram>

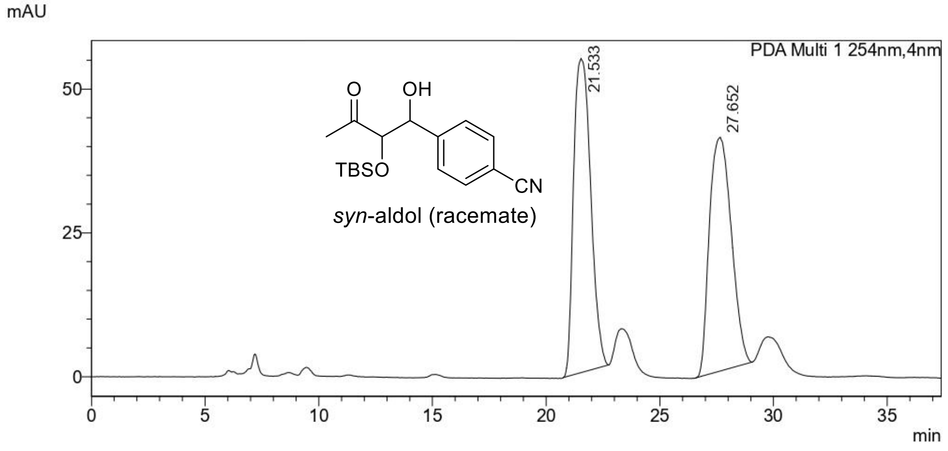

<Peak Table>

PDA Ch1 254nm

Peak#	Ret. Time	Area	Height	Area%
1	21.533	2867771	54630	51.151
2	27.652	2738710	40632	48.849
Total		5606481	95263	100.000

C:\Users\Shimadzu\Desktop\Ninos\Runs\NK43 run race 5%IPAnHex 20uL 0.5 mLmin 20Feb23 1mgml .lcd

Figure S60. HPLC trace of racemic *syn*(major)-aldol product **4i** (above).

SHIMADZU LabSolutions Analysis Report

<Sample Information>

Sample Name	: NK42 run Prod 5%IPAnHex 20uL 0.5 mLmin 20Feb23 1mgml .lcd		
Sample ID	:		
Data Filename	: NK42 run Prod 5%IPAnHex 20uL 0.5 mLmin 20Feb23 1mgml .lcd		
Method Filename	: trial.lcm		
Batch Filename	:		
Vial #	: 1-6	Sample Type	: Unknown
Injection Volume	: 20 uL		
Date Acquired	: 2/20/2023 2:01:33 PM	Acquired by	: System Administrator
Date Processed	: 2/20/2023 2:39:44 PM	Processed by	: System Administrator

<Chromatogram>

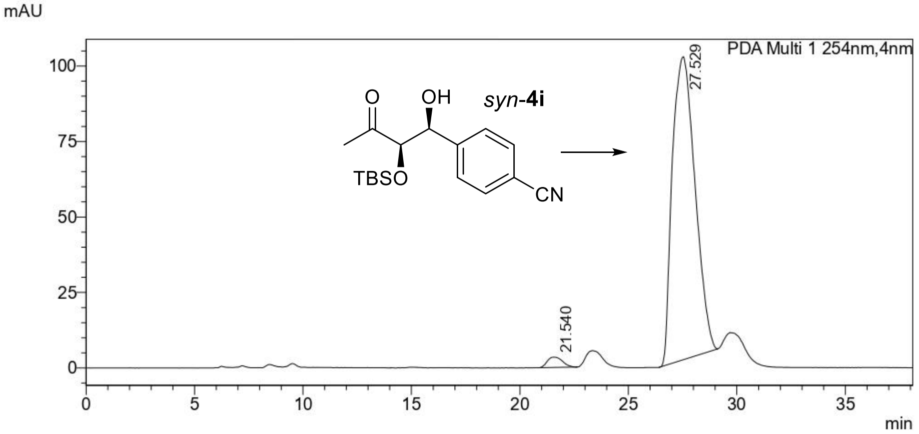

<Peak Table>

PDA Ch1 254nm

Peak#	Ret. Time	Area	Height	Area%
1	21.540	166617	3435	2.228
2	27.529	7311710	100336	97.772
Total		7478327	103771	100.000

C:\Users\Shimadzu\Desktop\Ninos\Runs\NK42 run Prod 5%IPAnHex 20uL 0.5 mLmin 20Feb23 1mgml .lcd

Figure S61. HPLC trace of the enantioenriched *syn*(major)-aldol product **4i** (above).

Acetylation of 4-((1S,2R)-2-((tert-butyldimethylsilyl)oxy)-1-hydroxy-3-oxobutyl)benzonitrile (4i)

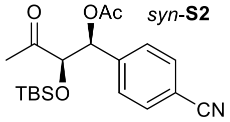

(1*S*,2*R*)-2-((*tert*-butyldimethylsilyl)oxy)-1-(4-cyanophenyl)-3-oxobutylacetate (S2)

To a clean round bottom flask (25 mL) equipped with a magnetic stir bar, were added 4-((1*S*,2*R*)-2-((*tert*-butyldimethylsilyl)oxy)-1-hydroxy-3-oxobutyl)benzonitrile (**4i**) (MW= 319.48, 1.0 equiv, 0.70 mmol, 225.0 mg), 4-dimethylaminopyridine (MW= 122.17, 1.2 equiv, 0.84 mmol, 102.7 mg). The round bottom flask was evacuated and flooded with nitrogen. Anhydrous dichloromethane (MW= 84.93, 0.09 M, density = 1.325 g/mL, 7.5 mL) was added, followed by triethylamine (MW= 101.19, 6.0 equiv, 4.20 mmol, density = 0.726 g/mL, 0.585 mL). Finally, acetic anhydride (MW= 102.09, 5.0 equiv, 3.5 mmol, density = 1.08 g/mL, 0.330 mL) was added dropwise over thirty seconds. The resulting solution was rigorously stirred for 36 h, but we inform the experimentalist that later experience informs us that 1-2 h is sufficient.

Work up: To a separatory funnel was added aqueous 0.5 N HCl (30 mL) and the reaction contents were then transferred to the separatory funnel. The round bottom flask was rinsed with CH_2Cl_2 (3 x 10 mL) and added to the separatory funnel. The CH_2Cl_2 layer was collected, and the aqueous solution was further extracted with CH_2Cl_2 (4 x 30.0 mL). The combined organic layers were dried over Na_2SO_4, filtered, and concentrated under rotary evaporation. The crude product was obtained after drying on high vacuum overnight.

Purification: Silica gel chromatography (3.5 cm column diameter, 16 cm silica gel height) was performed by adding the crude product dissolved in a minimum volume of CH_2Cl_2. Gradient elution was used (10 to 15% ethyl acetate in petroleum ether) and enabled isolation of the acetylated *syn*-product in pure form as a white solid. Note by TLC analysis this is a one spot to one spot reaction, unfortunately we did not record the yield but expect it to be quantitative.

TLC: R_f = 0.29, EtOAc/petroleum ether (15:85)

95% *ee*: Chiralpak OD-H, n-hexane/iPrOH (95:5), flow rate= 1.0 mL/min, λ = 254 nm, major *syn*-aldol product t_{minor} = 8.1 min t_{major}= 9.1 min (**Figure S64**).

^{1}H NMR (400 MHz, $CDCl_3$) (ppm) acetylated *syn*-aldol product **4i**: δ -0.37 (s, 3H), -0.07 (s, 3H), 0.87 (s, 9H), 2.13 (s, 3H), 2.16 (s, 3H), 4.18 (d, 1H, J= 3.9 Hz), 5.92 (dd, 1H, J= 2.75, 3.9 Hz), 7.43-7.45 (d, 2H, J= 8.6 Hz), 7.63-7.65 (d, 2H, J= 8.1 Hz).

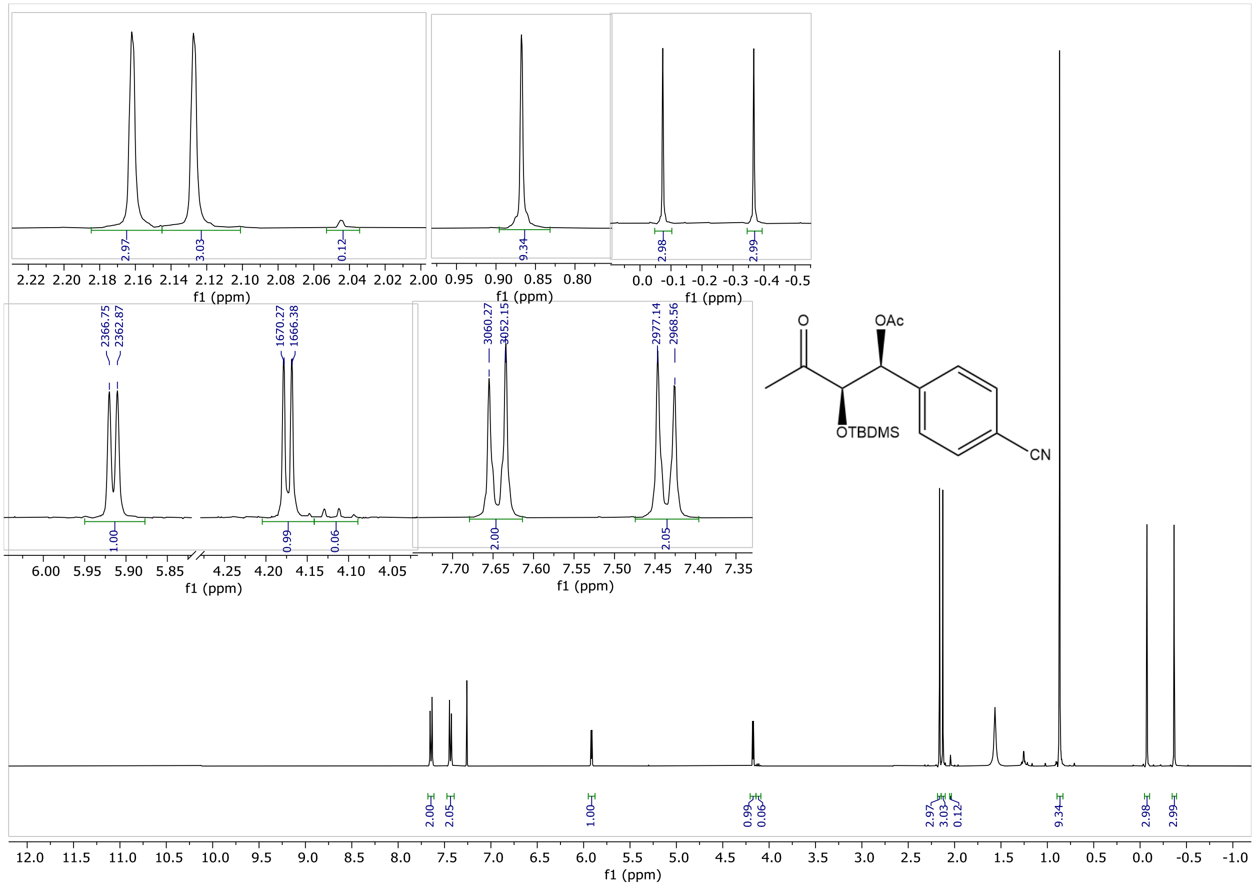

Figure S62. ^{1}H NMR spectrum of the purified acetylated *syn*-aldol product **S2** (above).

SHIMADZU LabSolutions **Analysis Report**

<Sample Information>

Sample Name	: NK54 5%IPAnHex 10uL 1.0 mLmin 9Apr23		
Sample ID	:		
Data Filename	: NK54 5%IPAnHex 10uL 1.0 mLmin 9Apr23.lcd		
Method Filename	: trial.lcm		
Batch Filename	:		
Vial #	: 1-7	Sample Type	: Unknown
Injection Volume	: 10 uL		
Date Acquired	: 4/9/2023 4:29:31 PM	Acquired by	: System Administrator
Date Processed	: 4/9/2023 4:45:06 PM	Processed by	: System Administrator

<Chromatogram>

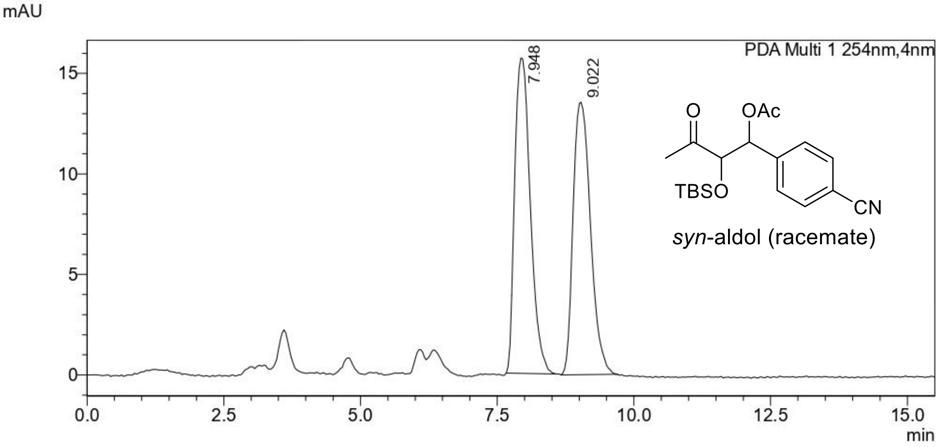

<Peak Table>

PDA Ch1 254nm

Peak#	Ret. Time	Area	Height	Area%
1	7.948	312878	15679	51.008
2	9.022	300516	13554	48.992
Total		613394	29233	100.000

C:\Users\Shimadzu\Desktop\Ninos\Runs\NK54 5%IPAnHex 10uL 1.0 mLmin 9Apr23.lcd

Figure S63. HPLC trace of racemic acetylated *syn*-aldol product **S2** (above).

SHIMADZU LabSolutions

Analysis Report

<Sample Information>

Sample Name	: NK50 5%IPAnHex 20uL 1.0 mLmin 24Mar23
Sample ID	:
Data Filename	: NK50 5%IPAnHex 10uL 1.0 mLmin 24Mar23.lcd
Method Filename	: trial.lcm
Batch Filename	:
Vial #	: 1-5
Sample Type	: Unknown
Injection Volume	: 10 uL
Date Acquired	: 3/24/2023 3:42:08 PM
Acquired by	: System Administrator
Date Processed	: 3/24/2023 4:01:10 PM
Processed by	: System Administrator

<Chromatogram>

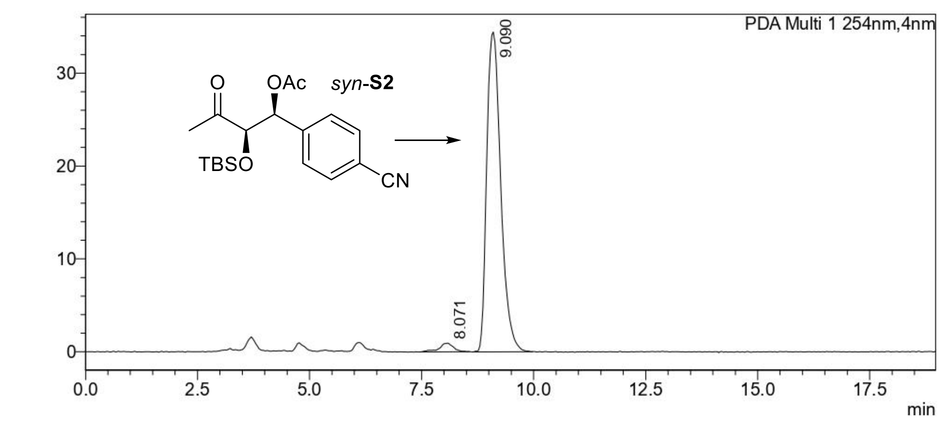

<Peak Table>

PDA Ch1 254nm

Peak#	Ret. Time	Area	Height	Area%
1	8.071	20715	922	2.687
2	9.090	750237	34412	97.313
Total		770952	35335	100.000

C:\Users\Shimadzu\Desktop\Ninos\Runs\NK50 5%IPAnHex 10uL 1.0 mLmin 24Mar23.lcd

Figure S64. HPLC trace of the enantioenriched acetylated *syn*-aldol product **S2** (above).

Table 3, entry 4: Competition reaction between 1-(t-butydimethylsilyloxy)-2-propanone and diethylmalonate for the limiting reactant 2-chlorobenzaldehyde

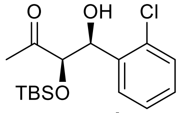

syn-**4j**

(3*R*,4*S*)-3-((*tert*-butyldimethylsilyl)oxy)-4-(2-chlorophenyl)-4-hydroxybutan-2-one

To a clean screw cap V-shaped reaction vessel (5.0 mL) equipped with a pyramidal stir bar, were added 1-(*t*-butydimethylsilyloxy)-2-propanone (MW= 188.34, 1.50 equiv, 2.25 mmol, density= 0.976 g/mL, 434 µL), diethylmalonate (MW= 160.17, 1.50 equiv, 2.25 mmol, density= 1.055 g/mL, 342 µL), O-tBu-L-threonine (MW= 175.23, 15 mol%, 0.225 mmol, 39.4 mg) and 2-chlorobenzaldehyde (MW= 140.57, 1.00 equiv, 1.50 mmol, density= 1.248 g/mL, 169 µL). Within one minute of adding 2-chlorobenzaldehyde, water (MW= 18.02, 15.0 equiv, 22.5 mmol, 405 µL) was added. The resulting heterogenous solution was rigorously stirred for 60 h such that an emulsion was always noted, albeit without causing splashing against the vessel walls; by doing so reproducible yield data was achieved.

This compound has been previously synthesized, see:
Kumar, A.; Singh, S.; Kumar, V.; Chimni, S. S. Asymmetric *syn*-selective direct aldol reaction of protected hydroxyacetone catalyzed by primary amino acid derived bifunctional organocatalyst in the presence of water. *Org. Biomol. Chem.* **2011**, *9*, 2731-2742.

Crude product ^{1}H NMR analysis (**Figure 65**, see below) allowed the following diastereo- and chemoselectivity ratios to be determined.

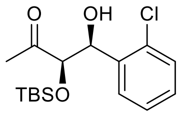

syn-aldol (major)

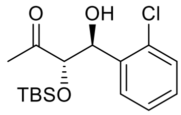

anti-aldol (minor)

Diastereoselectivity: The *syn*/*anti* ratio was determined as 7.7:1 by comparing the *syn*-aldol resonance at 4.42 ppm (d, representing the proton alpha to the ketone) and the *anti*-aldol resonance at 4.34 ppm (d, representing proton alpha to the ketone). Note the same reaction, albeit with the O-tBu-L-serine catalyst resulted in a 7:1 dr. For the literature regarding the *anti*-aldol product chemical shift, see page 2738 within A. Kumar, S. Singh, V. Kumar, S. S. Chimni, *Org. Biomol. Chem*. **2011**, *9*, 2731-2742.

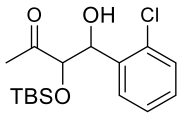

aldol products

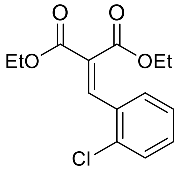

Knoevenagel product

Chemoselectivity: The aldol/Knoevenagel product ratio was determined to be >19:1. The Knoevenagel product has not been previously reported and we found no evidence for its structure within the crude 1H NMR spectrum.

Purification and yield: Silica gel chromatography (3.5 cm column diameter, 18 cm silica gel height) was performed by adding the crude product dissolved in a minimum volume of CH_2Cl_2. Gradient elution was used (0 to 8% EtOAc in petroleum ether). The *syn*- and *anti*-aldol products were isolated in pure form as a white solid (180.0 mg, MW= 328.91, 0.55 mmol, 37% yield). Note the same reaction, albeit with the O-tBu-L-serine catalyst resulted in a 30% isolated yield.

TLC: R_f = 0.29, EtOAc/petroleum ether (1:9)

The *ee* could not be determined: Chiralpak OD-H HPLC column, n-hexane/iPrOH (95:5), flow rate= 1.0 mL/min, λ = 254 nm (and variations thereof) did not result in separation of the *syn*-aldol (major) **4j** enantiomers, see HPLC chromatograms for the racemic and enantioenriched products in **Figures S67** and **S68** (below). The acetyl derivative of aldol product (*syn*-**S3**) was also synthesized, but again there was no separation of the major product enantiomers. For the acetylation procedure, ^1H NMR data and 'failed' HPLC chromatograms, see four pages forward.

^1H NMR (400 MHz, $CDCl_3$) (ppm) *syn*-aldol (major) product **4j**: δ -0.51 (s, 3H), -0.14 (s, 3H), 0.79 (s, 9H), 2.33 (s, 3H), 2.93 (d, 1H, OH, J= 9.26 Hz), 4.42 (d, 1H, J= 5.38 Hz), 5.37 (d, 1H, J= 9.26 Hz), 7.22-7.35 (m, 3H), 7.47 (d, 1H, J= 7.89).

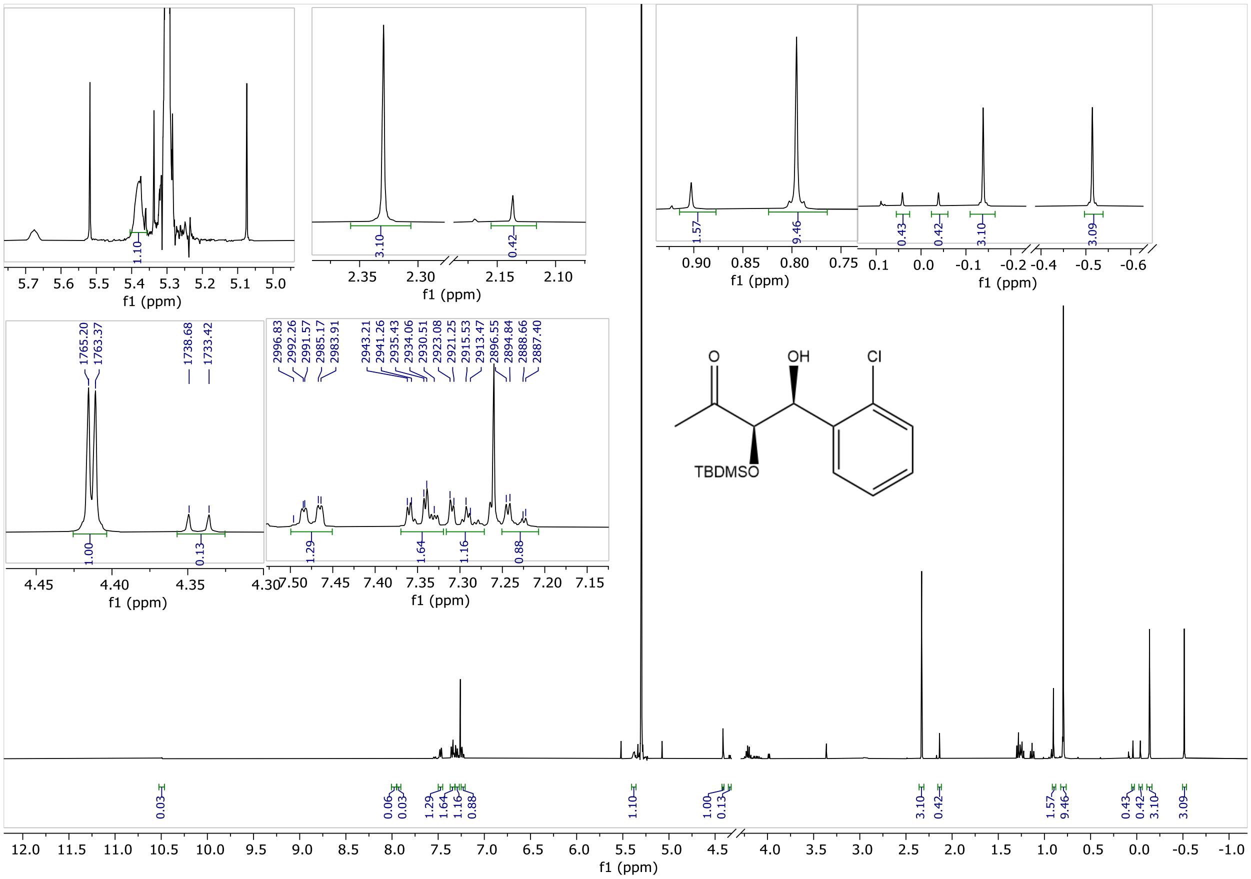

Figure S65. Crude ^1H NMR spectrum after high vacuum drying overnight (above).

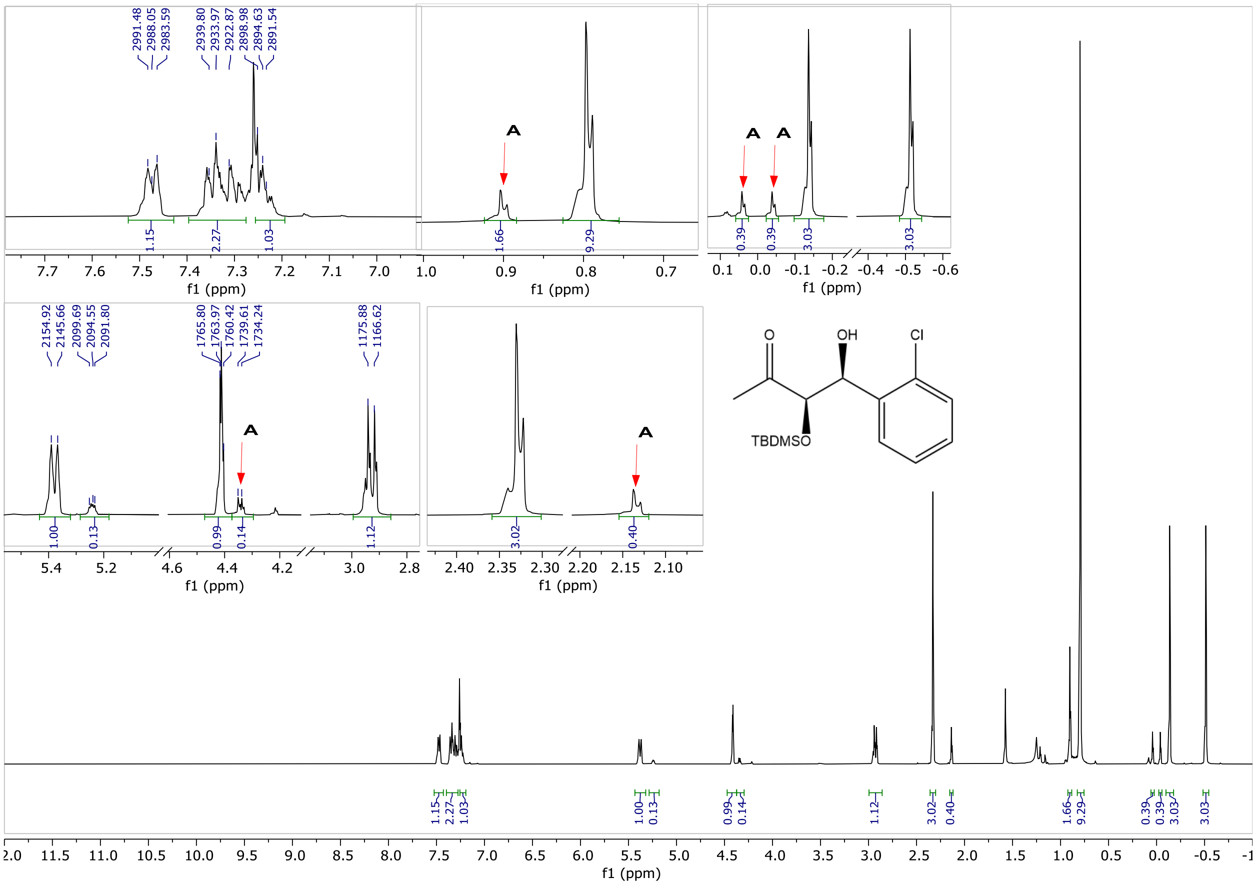

Figure S66. ^{1}H NMR spectrum of the purified *syn*-aldol product (above). We have followed the literature precedent and isolated the *syn*- and *anti*-aldol products together. Note the red arrows in the expansions show the minor *anti*-aldol product (A). For literature regarding the *anti*-aldol product chemical shift, please see page 2738 within A. Kumar, S. Singh, V. Kumar, S. S. Chimni, *Org. Biomol. Chem*. **2011**, *9*, 2731-2742.

SHIMADZU LabSolutions

Analysis Report

<Sample Information>

Sample Name	: NK48 5%IPAnHex 10uL 0.3 mLmin 25Mar23 trial 3		
Sample ID	:		
Data Filename	: NK48 5%IPAnHex 10uL 0.3 mLmin 25Mar23 trial 3.lcd		
Method Filename	: trial.lcm		
Batch Filename	:		
Vial #	: 1-4	Sample Type	: Unknown
Injection Volume	: 10 uL		
Date Acquired	: 3/25/2023 4:20:50 PM	Acquired by	: System Administrator
Date Processed	: 3/25/2023 4:47:54 PM	Processed by	: System Administrator

<Chromatogram>

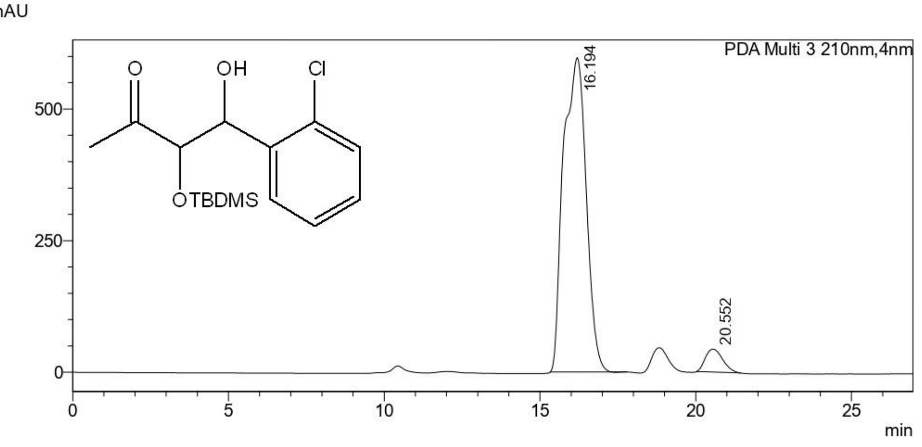

<Peak Table>

PDA Ch3 210nm

Peak#	Ret. Time	Area	Height	Area%
1	16.194	30361235	596785	94.718
2	20.552	1693127	43506	5.282
Total		32054362	640290	100.000

C:\Users\Shimadzu\Desktop\Ninos\Runs\NK48 5%IPAnHex 10uL 0.3 mLmin 25Mar23 trial 3.lcd

Figure S67. HPLC trace of racemic *syn*(major)-aldol product **4j** (above). No separation of *syn*-enantiomers (see the tallest peak with the left shoulder).

SHIMADZU LabSolutions **Analysis Report**

<Sample Information>

Sample Name	: NK47 5%IPAnHex 10uL 0.3 mLmin 25Mar23
Sample ID	:
Data Filename	: NK47 5%IPAnHex 10uL 0.3 mLmin 25Mar23.lcd
Method Filename	: trial.lcm
Batch Filename	:
Vial #	: 1-5
Sample Type	: Unknown
Injection Volume	: 10 uL
Date Acquired	: 3/25/2023 3:19:27 PM
Acquired by	: System Administrator
Date Processed	: 3/25/2023 3:47:30 PM
Processed by	: System Administrator

<Chromatogram>

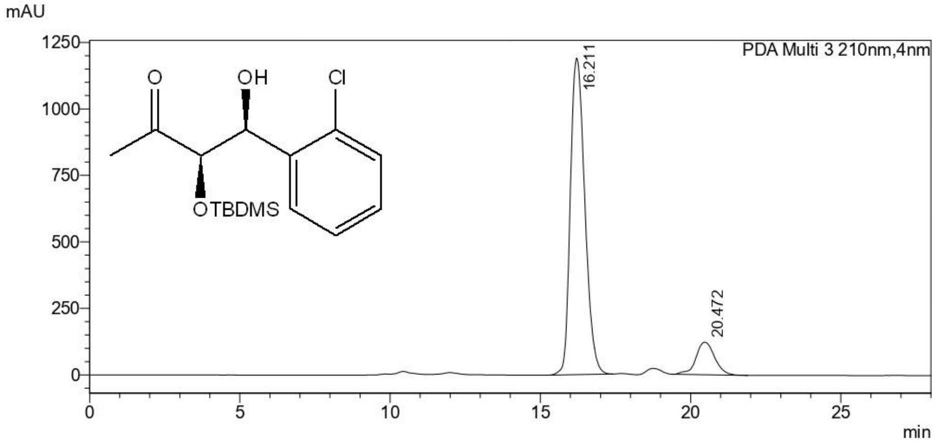

<Peak Table>

PDA Ch3 210nm

Peak#	Ret. Time	Area	Height	Area%
1	16.211	40012118	1189988	88.251
2	20.472	5326686	122495	11.749
Total		45338804	1312483	100.000

C:\Users\Shimadzu\Desktop\Ninos\Runs\NK47 5%IPAnHex 10uL 0.3 mLmin 25Mar23.lcd

Figure S68. HPLC trace of the enantioenriched *syn*(major)-aldol product **4j** (above). No separation of *syn*-enantiomers (see the tallest peak).

Acetylation of (3R,4S)-3-((tert-butyldimethylsilyl)oxy)-4-(2-chlorophenyl)-4-hydroxybutan-2-one (4j)

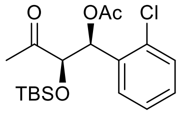

syn-**S3**

(1*S*,2*R*)-2-((*tert*-butyldimethylsilyl)oxy)-1-(2-chlorophenyl)-3-oxobutyl acetate (S3)

To a clean round bottom flask (50 mL) equipped with a magnetic stir bar, were added (3*R*,4*S*)-3-((*tert*-butyldimethylsilyl)oxy)-4-(2-chlorophenyl)-4-hydroxybutan-2-one(MW= 328.13, 1.0 equiv, 0.52 mmol, 169.4 mg), 4-dimethylaminopyridine (MW= 122.17, 1.2 equiv, 0.62 mmol, 75.7 mg). The round bottom flask was placed under nitrogen and anhydrous dichloromethane (MW= 84.93, 0.09 M, density = 1.325 g/mL, 6 mL) was added, followed by triethylamine (MW= 101.19, 6.0 equiv, 3.12 mmol, density = 0.726 g/mL, 0.435 mL). Finally, acetic anhydride (MW= 102.09, 5.0 equiv, 2.6 mmol, density = 1.08 g/mL, 0.319 mL) was added over a thirty second time period. The resulting solution was rigorously stirred for 2 hours and then worked-up.

Work up: To a separatory funnel was added aqueous 0.5 N HCl (30 mL) and the reaction contents were then transferred to the separatory funnel. The round bottom flask was rinsed with CH_2Cl_2 (3 x 10 mL) and added to the separatory funnel. The CH_2Cl_2 layer was collected, and the aqueous solution was further extracted with CH_2Cl_2 (4 x 30.0 mL). The combined organic layers were dried over Na_2SO_4, filtered, and concentrated under rotary evaporation. The crude product was obtained after drying on high vacuum overnight.

Purification: Silica gel chromatography (3.5 cm column diameter, 16 cm silica gel height) was performed by adding the crude product dissolved in a minimum volume of CH_2Cl_2. Gradient elution was used (5 to 10% ethyl acetate in petroleum ether). Note by TLC analysis this is a one spot to one spot reaction, unfortunately we did not record the yield but expect it to be quantitative.

TLC: R_f = 0.31, EtOAc/petroleum ether (15:85)

The *ee* could not be determined: Chiralpak OD-H, n-hexane/iPrOH (95:5), flow rate= 1.0 mL/min, λ = 210 nm, did not result in separation of the acetylated *syn*-aldol (major) product **S3** enantiomers. The *ee* could not be determined.

^{1}H NMR (400 MHz, $CDCl_3$) (ppm) acetylated *syn*-aldol product **S3**: δ -0.49 (s, 3H), -0.14 (s, 3H), 0.82 (s, 9H), 2.10 (s, 3H), 2.29 (s, 3H), 4.39 (d, 1H, J= 3.2 Hz), 6.40 (d, 1H, J= 3.32 Hz), 7.21-7.27 (m, 2H), 7.34-7.29 (m, 2H).

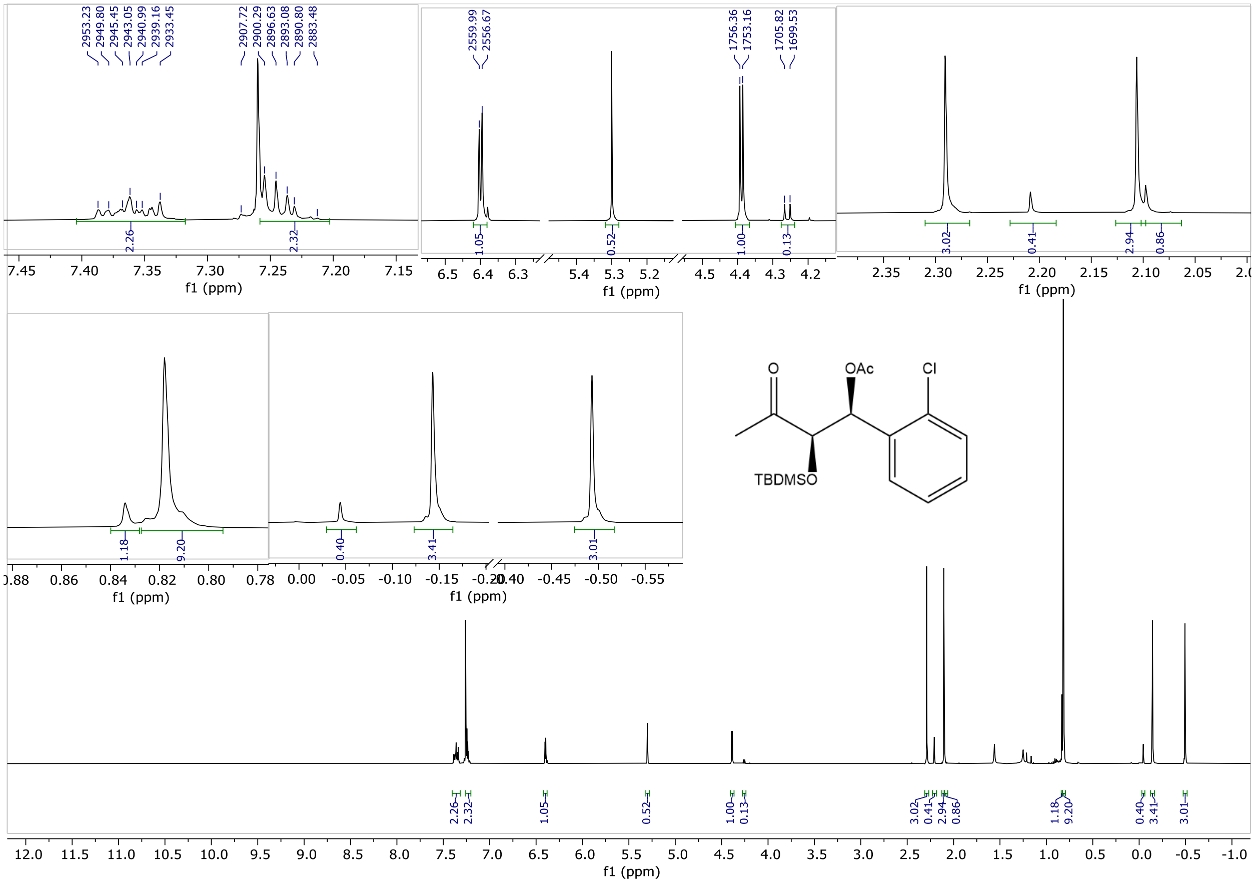

Figure S69. ^{1}H NMR spectrum of the purified acetylated *syn*-aldol product **S3** (above).

SHIMADZU LabSolutions **Analysis Report**

<Sample Information>

Sample Name	: NK62 race 5%IPAnHex 10ul 0.3mLmin 28.04.23		
Sample ID	:		
Data Filename	: NK62 race 5%IPAnHex 10ul 0.3mLmin 28.04.23.lcd		
Method Filename	: trial.lcm		
Batch Filename	:		
Vial #	: 1-5	Sample Type	: Unknown
Injection Volume	: 10 uL		
Date Acquired	: 4/28/2023 1:51:09 PM	Acquired by	: System Administrator
Date Processed	: 4/28/2023 2:12:59 PM	Processed by	: System Administrator

<Chromatogram>

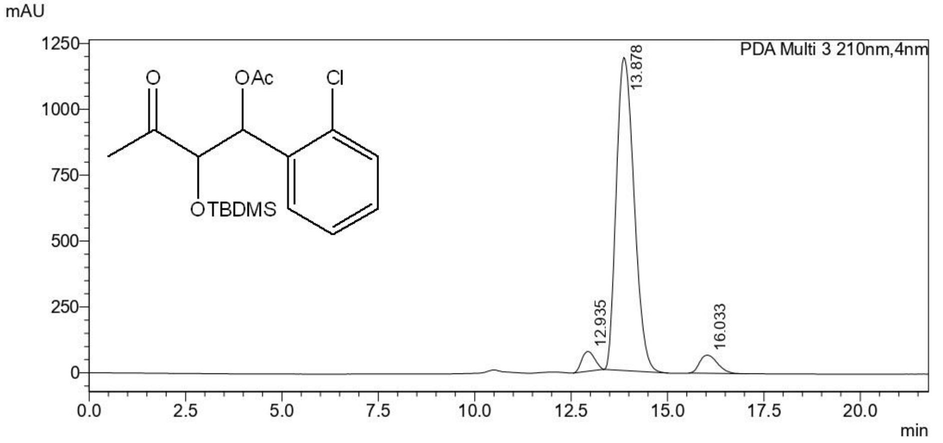

<Peak Table>

PDA Ch3 210nm

Peak#	Ret. Time	Area	Height	Area%
1	12.935	1748408	74743	4.144
2	13.878	38224800	1187616	90.602
3	16.033	2216525	68912	5.254
Total		42189733	1331272	100.000

C:\Users\Shimadzu\Desktop\Ninos\Runs\NK62 race 5%IPAnHex 10ul 0.3mLmin 28.04.23.lcd

Figure S70. HPLC trace of racemic acetylated *syn*-aldol (major) product **S3** (above). No separation of *syn*-enantiomers (see the tallest peak).

SHIMADZU LabSolutions

Analysis Report

<Sample Information>

Sample Name	: NK61 5%IPAnHex 10ul 0.3mLmin 28.04.23		
Sample ID	:		
Data Filename	: NK61 5%IPAnHex 10ul 0.3mLmin 28.04.23.lcd		
Method Filename	: trial.lcm		
Batch Filename	:		
Vial #	: 1-4	Sample Type	: Unknown
Injection Volume	: 10 uL		
Date Acquired	: 4/28/2023 1:27:08 PM	Acquired by	: System Administrator
Date Processed	: 4/28/2023 1:49:49 PM	Processed by	: System Administrator

<Chromatogram>

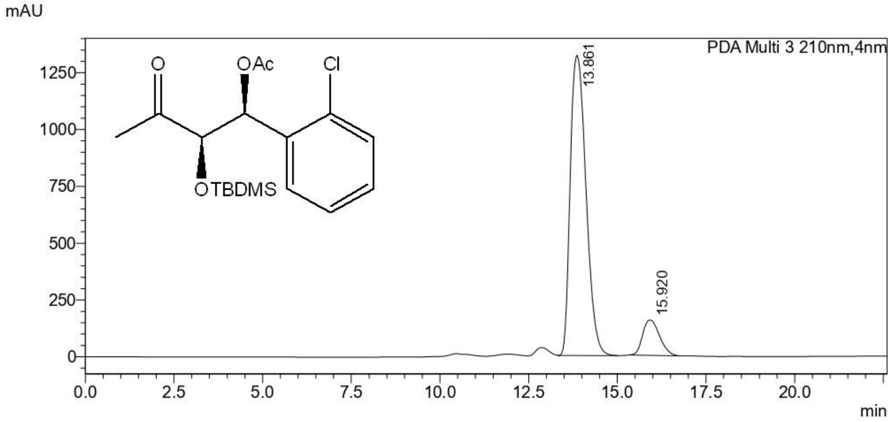

<Peak Table>

PDA Ch3 210nm

Peak#	Ret. Time	Area	Height	Area%
1	13.861	40767627	1321358	88.763
2	15.920	5160780	155839	11.237
Total		45928406	1477197	100.000

C:\Users\Shimadzu\Desktop\Ninos\Runs\NK61 5%IPAnHex 10ul 0.3mLmin 28.04.23.lcd

Figure 71. HPLC trace of the enantioenriched acylated *syn*-aldol (major) product **S3** (above). No separation of *syn*-enantiomers (see the tallest peak).

Section S8. Table 5 entries and characterization of *anti*-aldol product **4a**, **b**, **c**, **e, g, h, k**

Table 5, entry 1: Competition reaction between cyclohexanone and acetylacetone for the limiting reactant 4-nitrobenzaldehyde

(*S*)-2-[(*R*)-hydroxy(4-nitrophenyl)methyl]cyclohexanone

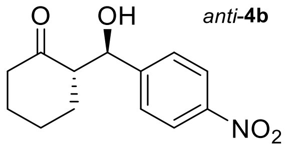

To a clean Eppendorf vial (3 mL) were added cyclohexanone (MW= 98.14, 1.50 equiv, 2.25 mmol, 220.8 mg, density= 1.055 g/mL, 233 μL), acetylacetone (MW= 100.12, 1.5 equiv, 2.25 mmol, density= 0.975 g/mL, 231 μL), 4-nitrobenzaldehyde (MW= 151.12, 1.0 equiv, 1.50 mmol, 227 mg), *trans*-4-(*tert*-butyldiphenylsilyloxy)-L-proline catalyst (MW= 369.54, 5 mol%, mmol, 27.7 mg), Choline chloride/Urea 1/2 DES (5.78 mmol, 500 mg, 400 μL) and H_2O (11.11 mmol, 200 mg, 200 μL) in the stated order. The reaction was mixed in a ball mill with 5 stainless steel balls for 7 h at a frequency of 15.5 s^{-1}.

Table 5, entry 2: Competition reaction between cyclohexanone and methylsulfonylacetone for the limiting reactant 4-nitrobenzaldehyde

(*S*)-2-[(*R*)-hydroxy(4-nitrophenyl)methyl]cyclohexanone

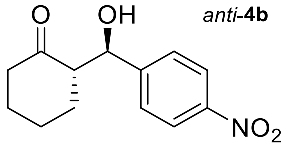

To a clean Eppendorf vial (3 mL) were added cyclohexanone (MW= 98.14, 1.50 equiv, 2.25 mmol, 220.8 mg, density= 1.055 g/mL, 233 μL), methylsulfonylacetone (MW= 136.17, 1.5 equiv, 2.25 mmol, 306.3 mg), 4-nitrobenzaldehyde (MW= 151.12, 1.0 equiv, 1.50 mmol, 227 mg), *trans*-4-(*tert*-butyldiphenylsilyloxy)-L-proline catalyst (MW= 369.54, 5 mol%, mmol, 27.7 mg), Choline chloride/Urea 1/2 DES (5.78 mmol, 500 mg, 400 μL) and H_2O (11.11 mmol, 200 mg, 200 μL) in the stated order. The reaction was mixed in a ball mill with 5 stainless steel balls for 7 h at a frequency of 15.5 s^{-1}.

Table 5, entry 3: Competition reaction between cyclohexanone and acetylacetone for the limiting reactant 4-(trifluoromethyl)benzaldehyde

(*S*)-2-[(*R*)-hydroxy(4-(trifluoromethyl)phenyl)methyl]cyclohexanone

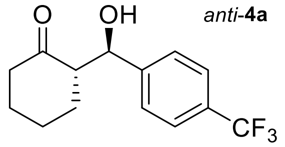

anti-**4a**

To a clean Eppendorf vial (3 mL) were added cyclohexanone (MW= 98.14, 1.50 equiv, 2.25 mmol, 220.8 mg, density= 1.055 g/mL, 233 μL), acetylacetone (MW= 100.12, 1.5 equiv, 2.25 mmol, density= 0.975 g/mL, 231 μL), 4-(trifluoromethyl)benzaldehyde (MW= 174.12, 1.0 equiv, 1.50 mmol, 261 mg, density=1.275 g/mL, 205 μL), *trans*-4-(*tert*-butyldiphenylsilyloxy)-L-proline catalyst (MW= 369.54, 5 mol%, mmol, 27.7 mg), Choline chloride/Urea 1/2 DES (5.78 mmol, 500 mg, 400 μL) and H_2O (11.11 mmol, 200 mg, 200 μL) in the stated order. The reaction was mixed in a ball mill with 5 stainless steel balls for 7 h at a frequency of 15.5 s^{-1}.

Table 5, entry 4: Competition reaction between cyclohexanone and acetylacetone for the limiting reactant methyl 4-formylbenzoate

Methyl 4-[(*R*)-hydroxy((*S*)-2-oxocyclohexyl)methyl]benzoate

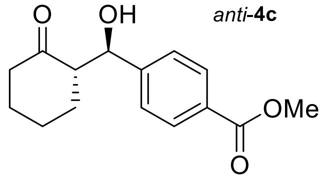

anti-**4c**

To a clean Eppendorf vial (3 mL) were added cyclohexanone (MW= 98.14, 1.50 equiv, 2.25 mmol, 220.8 mg, density= 1.055 g/mL, 233 μL), acetylacetone (MW= 100.12, 1.5 equiv, 2.25 mmol, density= 0.975 g/mL, 231 μL), methyl 4-formylbenzoate (MW= 164.16, 1.0 equiv, 1.50 mmol, 246 mg), *trans*-4-(*tert*-butyldiphenylsilyloxy)-L-proline catalyst (MW= 369.54, 5 mol%, mmol, 27.7 mg), Choline chloride/Urea 1/2 DES (5.78 mmol, 500 mg, 400 μL) and H_2O (11.11 mmol, 200 mg, 200 μL) in the stated order. The reaction was mixed in a ball mill with 5 stainless steel balls for 7 h at a frequency of 15.5 s^{-1}.

Table 5, entry 5: Competition reaction between tetrahydro-4*H*-thiopyran-4-one and acetylacetone for the limiting reactant 4-nitrobenzaldehyde

(*S*)-3-[(*R*)-hydroxy(4-nitrophenyl)methyl]tetrahydro-4*H*-thiopyran-4-one (**4k**)

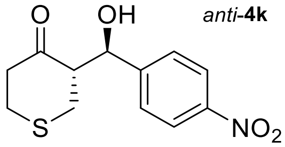
anti-**4k**

To a clean Eppendorf vial (3 mL) were added tetrahydro-4*H*-thiopyran-4-one (MW= 116.18, 1.50 equiv, 2.25 mmol, 261.4 mg), acetylacetone (MW= 100.12, 1.5 equiv, 2.25 mmol, density= 0.975 g/mL, 231 μL), 4-nitrobenzaldehyde (MW= 151.12, 1.0 equiv, 1.50 mmol, 227 mg), *trans*-4-(*tert*-butyldiphenylsilyloxy)-L-proline catalyst (MW= 369.54, 5 mol%, mmol, 27.7 mg), Choline chloride/Urea 1/2 DES (5.78 mmol, 500 mg, 400 μL) and H_2O (11.11 mmol, 200 mg, 200 μL) in the stated order. The reaction was mixed in a ball mill with 5 stainless steel balls for 7 h at a frequency of 15.5 s^{-1}.

Crude product ^1H NMR analysis (**Figure S72**, below) allowed the following diastereo- and chemoselectivity ratios to be determined.

Conversion: the reaction conversion was determined to be 88%, based on the *anti*-aldol product resonance at 5.06 ppm (d, representing the benzylic proton) and *syn*-aldol product resonance at 5.50 ppm (d, representing the benzylic proton) *versus* the 4-nitrobenzaldehyde resonance at 10.15 ppm (s, representing the aldehyde proton) observed in the ^1H NMR spectrum of the crude reaction mixture.

Diastereoselectivity: the *anti*/*syn* aldol product ratio was determined to be >49/1, based on the *anti*-aldol product resonance at 5.06 ppm (d, representing the benzylic proton) and the *syn*-aldol product resonance at 5.50 ppm (d, representing the benzylic proton) observed in the ^1H NMR spectrum of the crude reaction mixture.

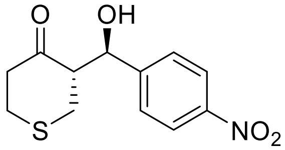
anti-aldol

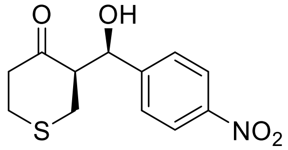
syn-aldol

Chemoselectivity: the aldol/Knoevenagel product ratio was determined to be 6/1, based on the addition of the *anti*-aldol product resonance at 5.06 ppm (d, representing the benzylic proton)

and *syn*-aldol product resonance at 5.50 ppm (d, representing the benzylic proton) *versus* the Knoevenagel product resonance at 2.28 ppm (s, representing one of the methyl groups) observed in the ^{1}H NMR spectrum of the crude reaction mixture.

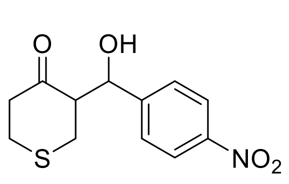

aldol products

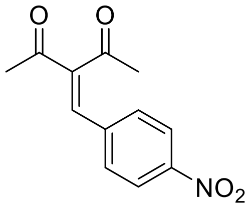

Knoevenagel product

Purification and yield: silica gel chromatography (35 mm column outer diameter, 16 cm silica bed height) was performed by adding the crude product in silica pellet. Gradient elution was used (13 to 25 vol% EtOAc in hexane). 260 mg (MW= 267, mmol, 65% yield) of the *anti*-/*syn*- aldol products **4k** was isolated as a light-yellow solid.

TLC: R_f = 0.3, Hexane/EtOAc (4:1).

98% *ee*: Chiralpak AD-H, iPrOH/n-hexane (10:90), Flow rate = 1.0 mL/min, λ= 254 nm, *anti*-aldol product t_{major}= 46.8 min, t_{minor}= 82.9 min was observed (**Figure S75**).

^{1}H NMR (400MHz, $CDCl_3$) (ppm) *anti*-aldol (major) product: δ 8.23 (d, 2H, J= 8 Hz), 7.54 (d, 2H, J= 8 Hz), 5.04 (dd, 1H, J= 4 and 8 Hz), 3.61 (d, 1H, J= 4 Hz), 3.05-2.96 (m, 3H), 2.84-2.81 (m, 2H), 2.71-2.64 (m, 1H) and 2.55-2.50 (m, 1H).

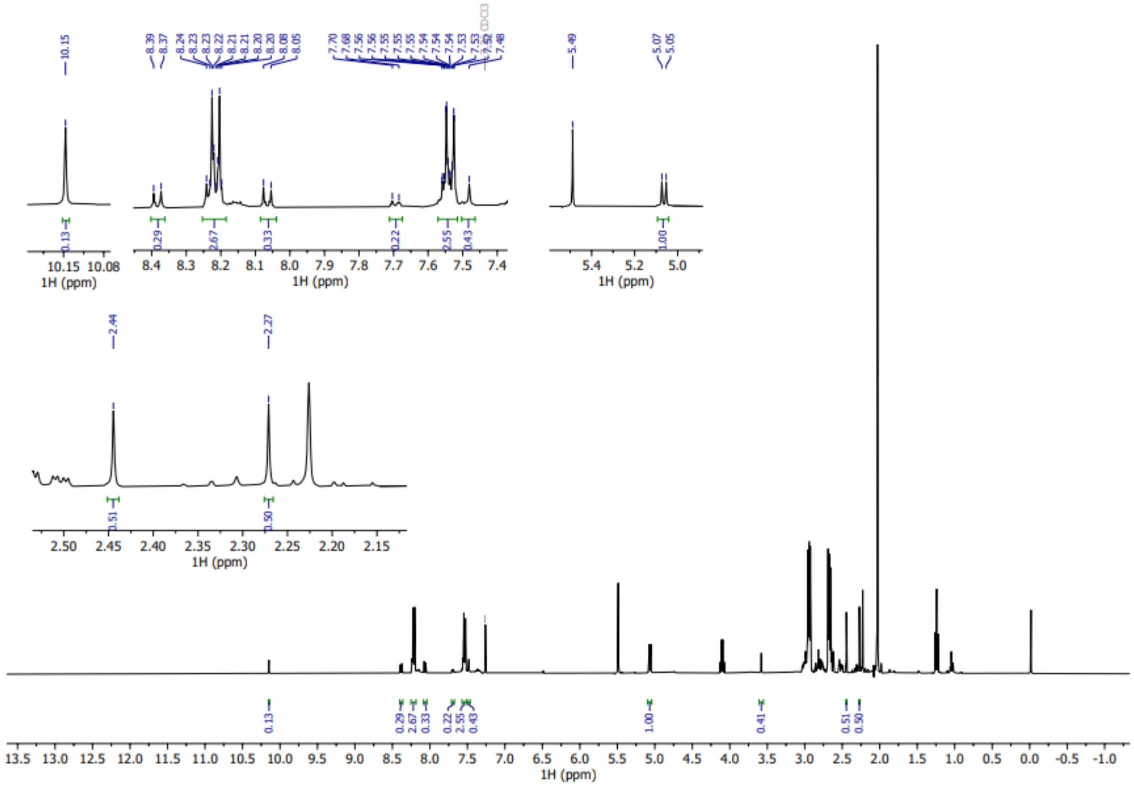

Figure S72. Crude ^{1}H NMR spectrum (above). Note that traces of EtOAc are present, and the signal at 5.5 ppm is not representing the *syn*-aldol but the sp^2-proton from the enol of the equilibrium of acetylacetone.

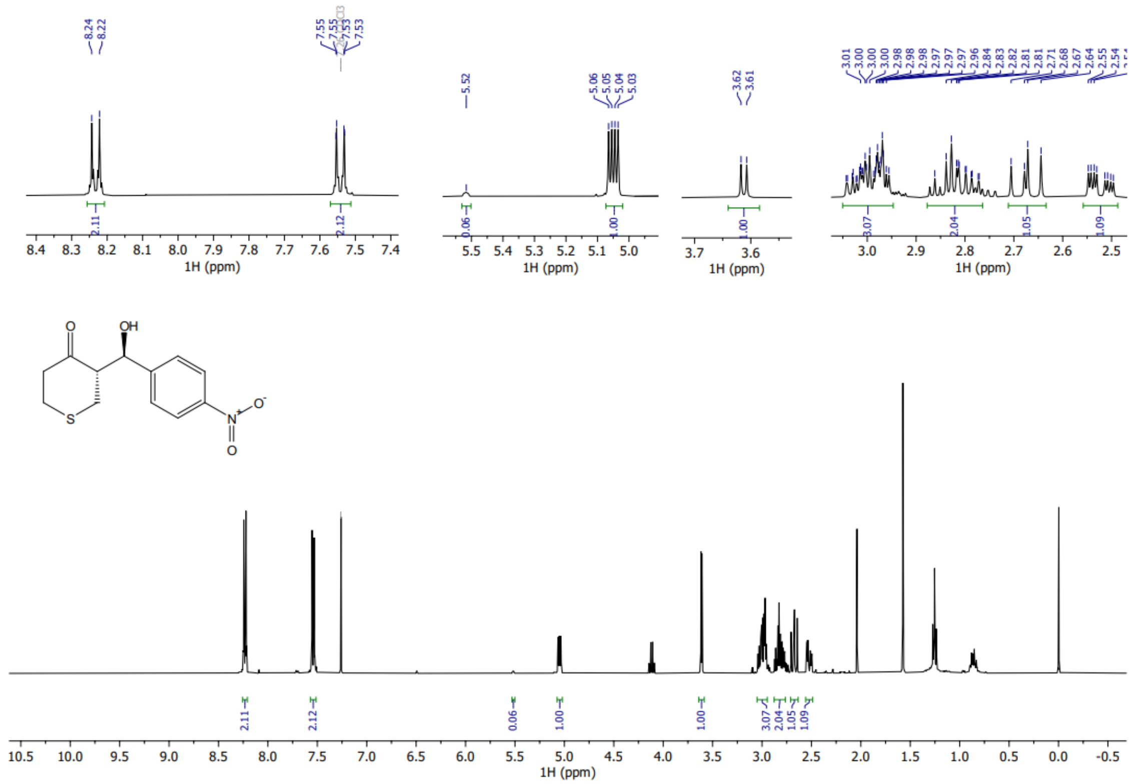

Figure S73. ^{1}H NMR spectrum of the purified anti-(major) and syn-(minor) aldol products (above) with some traces of EtOAc.

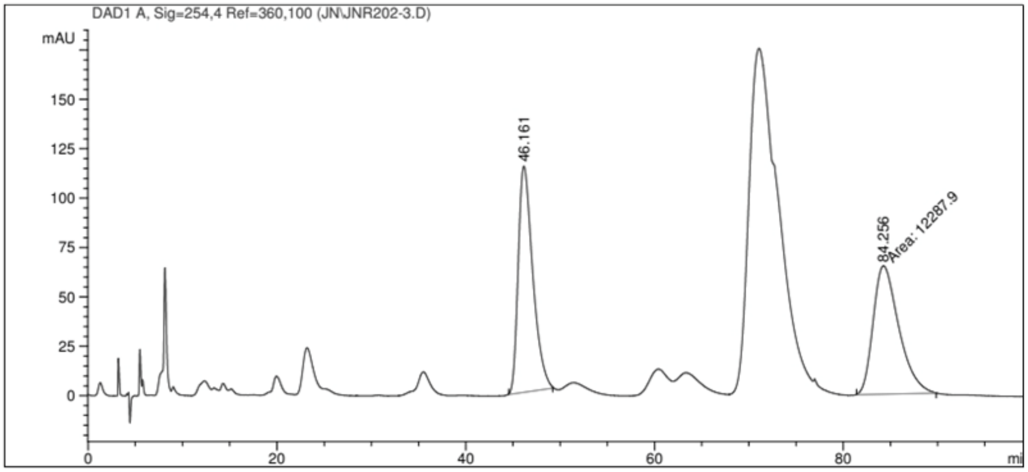

Peak #	RetTime [min]	Type	Width [min]	Area [mAU*s]	Height [mAU]	Area %
1	46.161	BB	1.4815	1.22669e4	114.53281	49.9571
2	84.256	MM	3.1545	1.22879e4	64.92210	50.0429
Totals :				2.45548e4	179.45490	

Figure S74. HPLC trace of the crude reaction mixture of racemic aldol product **4k** (above).

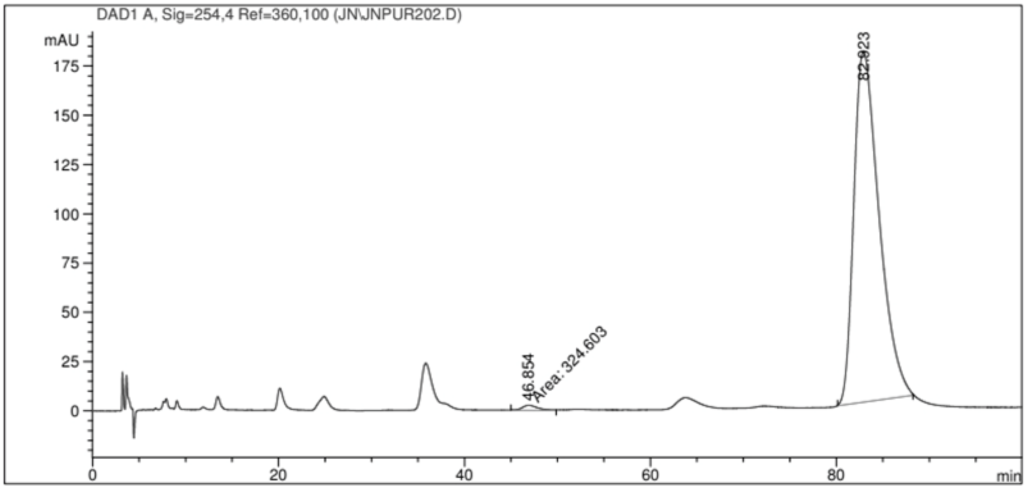

Peak #	RetTime [min]	Type	Width [min]	Area [mAU*s]	Height [mAU]	Area %
1	46.854	MM	2.0566	324.60284	2.63062	0.9559
2	82.923	BB	2.2300	3.36338e4	178.20042	99.0441
Totals :				3.39584e4	180.83104	

Figure S75. HPLC trace of the pure enantioenriched *anti*-aldol (major) product **4k** (above).

Table 5, entry 6: Competition reaction between tetrahydro-4*H*-thiopyran-4-one and methylsulfonylacetone for the limiting reactant 4-nitrobenzaldehyde

(*S*)-3-[(*R*)-hydroxy(4-nitrophenyl)methyl]tetrahydro-4*H*-thiopyran-4-one

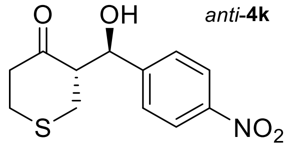

anti-**4k**

To a clean Eppendorf vial (3 mL) were added tetrahydro-4*H*-thiopyran-4-one (MW= 116.18, 1.50 equiv, 2.25 mmol, 261.4 mg), methylsulfonylacetone (MW= 136.17, 1.5 equiv, 2.25 mmol, 306.3 mg), 4-nitrobenzaldehyde (MW= 151.12, 1.0 equiv, 1.50 mmol, 227 mg), *trans*-4-(*tert*-butyldiphenylsilyloxy)-L-proline catalyst (MW= 369.54, 5 mol%, mmol, 27.7 mg), Choline chloride/Urea 1/2 DES (5.78 mmol, 500 mg, 400 μL) and H_2O (11.11 mmol, 200 mg, 200 μL) in the stated order. The reaction was mixed in a ball mill with 5 stainless steel balls for 7 h at a frequency of 15.5 s^{-1}.

Crude product ^1H NMR analysis (**Figure S76**, below) allowed the following diastereo- and chemoselectivity ratios to be determined.

Conversion: the reaction conversion was determined to be 96%, based on the the *anti*-aldol product resonance at 5.06 ppm (d, representing the benzylic proton) and *syn*-aldol product resonance at 5.50 ppm (d, representing the benzylic proton) versus the 4-nitrobenzaldehyde resonance at 10.15 ppm (s, representing the aldehyde proton) observed in the ^1H NMR spectrum of the crude reaction mixture.

Diastereoselectivity: the *anti*/*syn* aldol product ratio was determined to be 13/1, based on the *anti*-aldol product resonance at 5.06 ppm (d, representing the benzylic proton) and the *syn*-aldol product resonance at 5.50 ppm (d, representing the benzylic proton) observed in the ^1H NMR spectrum of the crude reaction mixture.

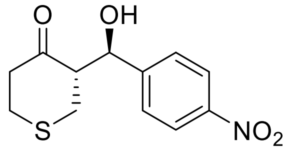

anti-aldol

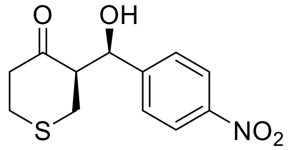

syn-aldol

Chemoselectivity: the aldol/Knoevenagel product ratio was determined to be 6/1, based on the addition of the *anti*-aldol product resonance at 5.06 ppm (d, representing the benzylic proton) and *syn*-aldol product resonance at 5.50 ppm (d, representing the benzylic proton) versus the Knoevenagel product resonance at 2.28 ppm (s, representing one of the methyl groups) observed in the ^1H NMR spectrum of the crude reaction mixture.

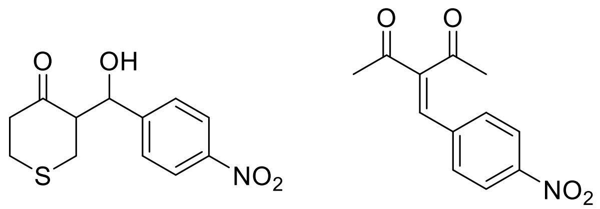

aldol products　　Knoevenagel product

Purification and yield: silica gel chromatography (35 mm column outer diameter, 16 cm silica bed height) was performed by adding the crude product in silica pellet. Gradient elution was used (13 to 25 vol% EtOAc in hexane). 256 mg (MW= 267, mmol, 64% yield) of the *anti-*/*syn-* aldol products 4k was isolated as a light-yellow solid.

TLC: R_f = 0.3, Hexane/EtOAc (4:1).

96% *ee*: Chiralpak AD-H, iPrOH/n-hexane (10:90), Flow rate = 1.0 mL/min, λ= 254 nm, *anti*-aldol product t_{major}= 46.9 min, t_{minor}= 83.9 min was observed (**Figure S79**).

^{1}H NMR (400MHz, CDCl$_3$) (ppm): *anti*-aldol (major) product: δ 8.24 (d, 2H, J= 8 Hz), 7.55 (d, 2H, J= 8 Hz), 5.04 (dd, 1H, J= 4 and 8 Hz), 3.61 (d, 1H, J= 4 Hz), 3.05-2.95 (m, 3H), 2.84-2.79 (m, 2H), 2.72-2.64 (m, 1H) and 2.55-2.50 (m, 1H).

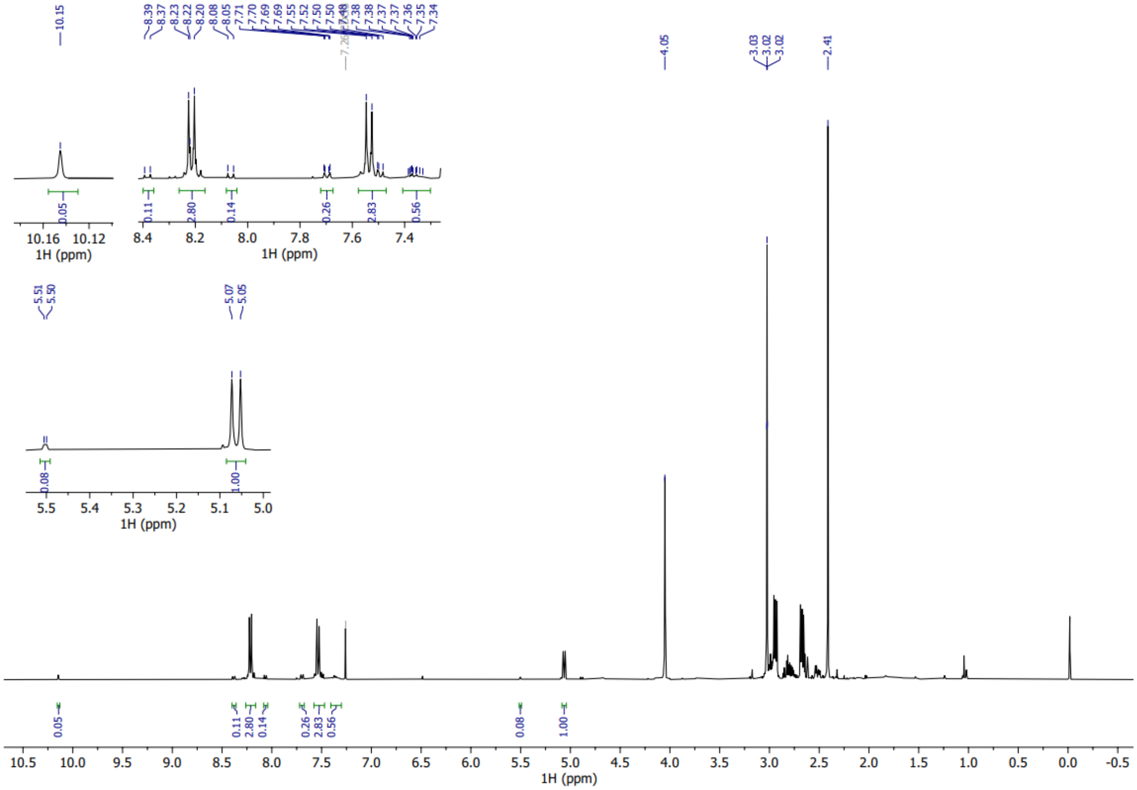

Figure S76. Crude ^{1}H NMR spectrum (above). Signal (s) at 4.05, 3.02 and 2.41 belong to methylsulfonylacetone.

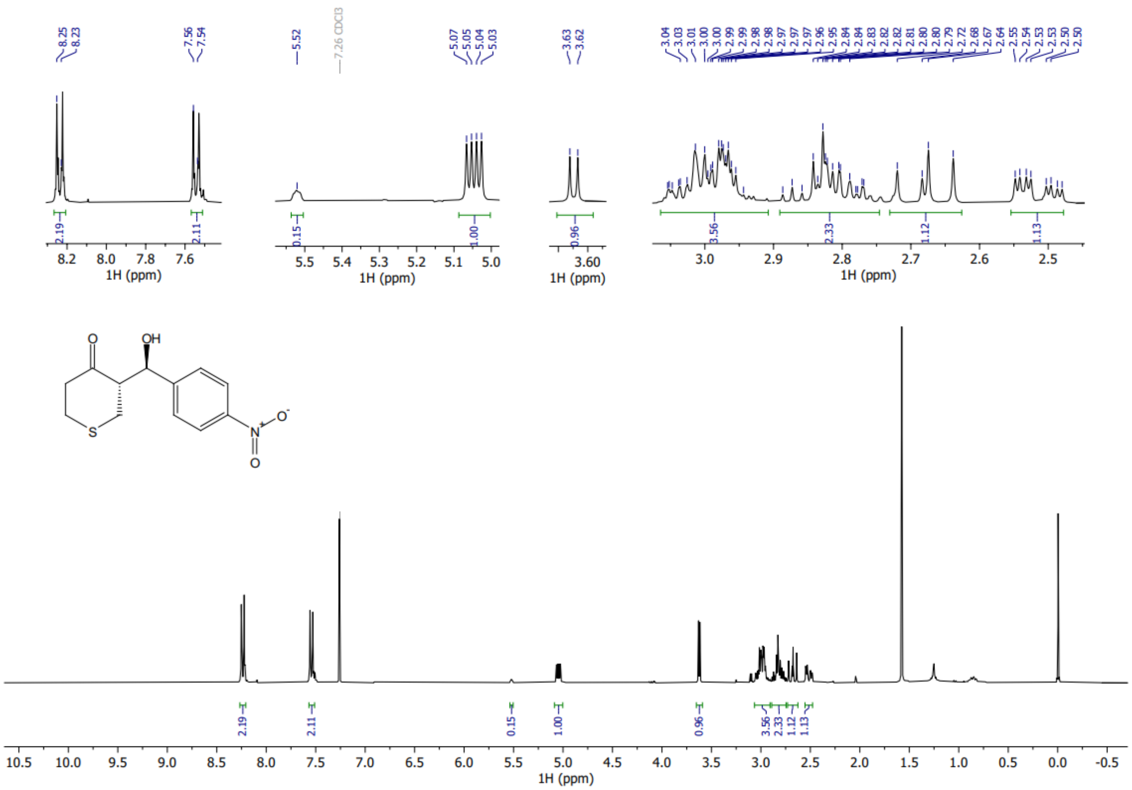

Figure S77. ^{1}H NMR spectrum of the purified anti-(major) and syn-(minor) aldol products (above).

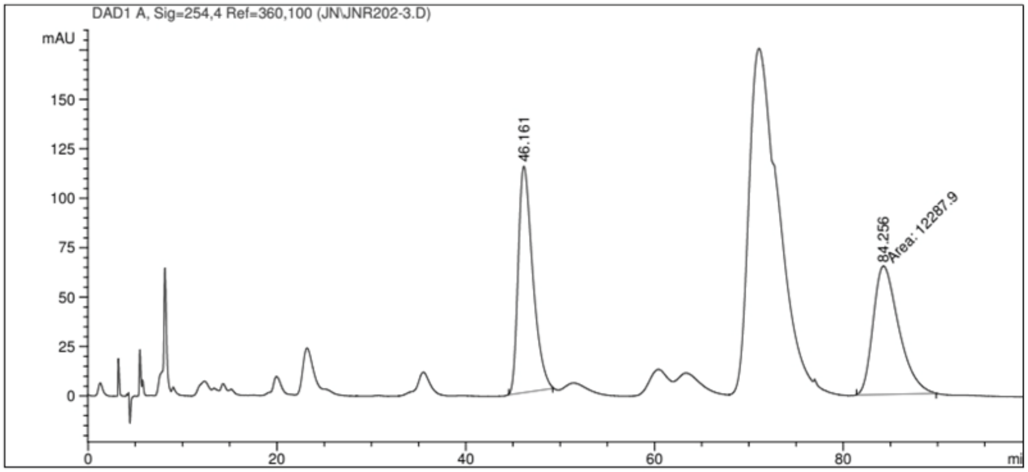

Peak #	RetTime [min]	Type	Width [min]	Area [mAU*s]	Height [mAU]	Area %
1	46.161	BB	1.4815	1.22669e4	114.53281	49.9571
2	84.256	MM	3.1545	1.22879e4	64.92210	50.0429
Totals :				2.45548e4	179.45490	

Figure S78. HPLC trace of the crude reaction mixture of racemic aldol product **4k** (above).

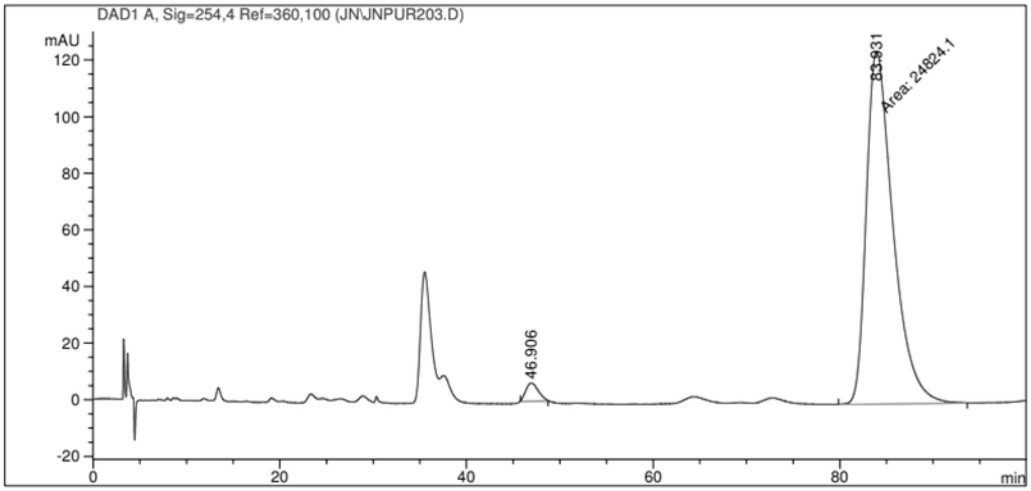

Peak #	RetTime [min]	Type	Width [min]	Area [mAU*s]	Height [mAU]	Area %
1	46.906	BB	1.0949	599.04382	6.45389	2.3563
2	83.931	MM	3.3186	2.48241e4	124.67222	97.6437
Totals :				2.54231e4	131.12611	

Figure S79. HPLC trace of the pure enantioenriched *anti*-aldol (major) product **4k** (above).

Table 5, entry 7: Competition reaction between 2,2-dimethyl-1,3-dioxan-5-one and acetylacetone for the limiting reactant 4-nitrobenzaldehyde

(*S*)-4-[(*S*)-hydroxy(4-nitrophenyl)methyl]-2,2-dimethyl-1,3-dioxan-5-one

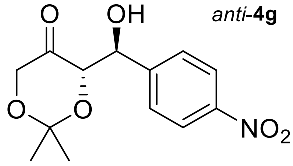

anti-**4g**

To a clean Eppendorf vial (3 mL) were added 2,2-dimethyl-1,3-dioxan-5-one (MW= 130.14, 1.50 equiv, 2.25 mmol, 292.8 mg, density= 1.09 g/mL, 268 μL), acetylacetone (MW= 100.12, 1.5 equiv, 2.25 mmol, density= 0.975 g/mL, 231 μL), 4-nitrobenzaldehyde (MW= 151.12, 1.0 equiv, 1.50 mmol, 227 mg), *trans*-4-(*tert*-butyldiphenylsilyloxy)-L-proline catalyst (MW= 369.54, 5 mol%, mmol, 27.7 mg), Choline chloride/Urea 1/2 DES (5.78 mmol, 500 mg, 400 μL) and H_2O (11.11 mmol, 200 mg, 200 μL) in the stated order. The reaction was mixed in a ball mill with 5 stainless steel balls for 7 h at a frequency of 15.5 s^{-1}.

Table 5, entry 8: Competition reaction between 4-methylciclohexanone and acetylacetone for the limiting reactant 4-nitrobenzaldehyde

(2*S*)-2-[(*R*)-hydroxy(4-nitrophenyl)methyl)-4-methylcyclohexanone

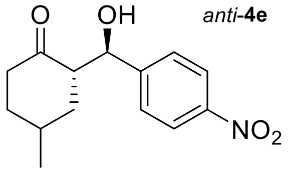

anti-**4e**

To a clean Eppendorf vial (3 mL) were added 4-methylcyclohexanone (MW= 112.17, 1.50 equiv, 2.25 mmol, 252.4 mg, density= 0.914 g/mL, 276 μL), acetylacetone (MW= 100.12, 1.5 equiv, 2.25 mmol, density= 0.975 g/mL, 231 μL), 4-nitrobenzaldehyde (MW= 151.12, 1.0 equiv, 1.50 mmol, 227 mg), *trans*-4-(*tert*-butyldiphenylsilyloxy)-L-proline catalyst (MW= 369.54, 5 mol%, mmol, 27.7 mg), Choline chloride/Urea 1/2 DES (5.78 mmol, 500 mg, 400 μL) and H_2O (11.11 mmol, 200 mg, 200 μL) in the stated order. The reaction was mixed in a ball mill with 5 stainless steel balls for 7 h at a frequency of 15.5 s^{-1}.

Table 5, entry 9: Competition reaction between 1-((*tert*-butyldimethylsilyloxy)propan-2-one and acetylacetone for the limiting reactant 4-nitrobenzaldehyde

(3S,4S)-3-[(tert-butyldimethylsilyl)oxy]-4-hydroxy-4-(4-nitrophenyl)butan-2-one

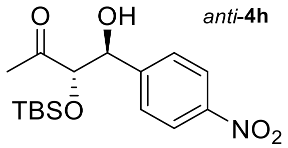

anti-**4h**

To a clean Eppendorf vial (3 mL) were added 1-((*tert*-butyldimethylsilyloxy)propan-2-one (MW= 188.34, 1.50 equiv, 2.25 mmol, 423.7 mg, density= 0.976 g/mL, 434 μL), acetylacetone (MW= 100.12, 1.5 equiv, 2.25 mmol, density= 0.975 g/mL, 231 μL), 4-nitrobenzaldehyde (MW= 151.12, 1.0 equiv, 1.50 mmol, 227 mg), *trans*-4-(*tert*-butyldiphenylsilyloxy)-L-proline catalyst (MW= 369.54, 5 mol%, mmol, 27.7 mg), Choline chloride/Urea 1/2 DES (5.78 mmol, 500 mg, 400 μL) and H_2O (11.11 mmol, 200 mg, 200 μL) in the stated order. The reaction was mixed in a ball mill with 5 stainless steel balls for 7 h at a frequency of 15.5 s^{-1}.

Section S9. Knoevenagel selective competition reaction.

Reaction of cyclohexanone and acetylacetone for the limiting reagent 4-nitrobenzaldehyde in dry DMSO-d$_6$

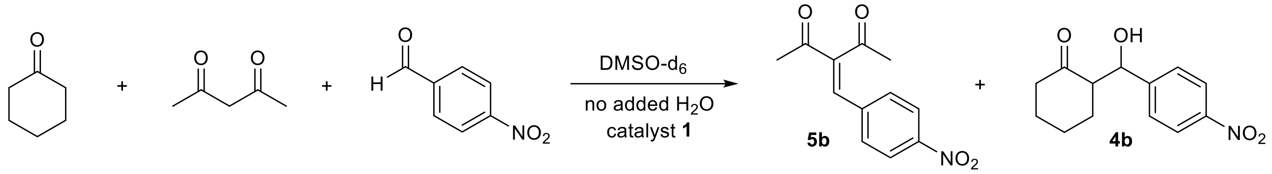

Set-up: To a clean screw cap V-shaped reaction vessel (5 mL) equipped with a stir bar were subsequently added *trans*-4-(*tert*-butyldiphenylsilyloxy)-*L*-proline catalyst (MW = 369.54, 30 mol%, 0.30 mmol, 110.9 mg), 4-nitrobenzaldehyde (MW = 151.12, 1.0 equiv, 1.0 mmol, 151.1 mg), *d$_6$*-DMSO (1.4 mL, 0.71 M), cyclohexanone (MW = 98.14, 1.5 equiv, 1.5 mmol, density = 0.948 g/mL, V = 155 µL), and acetylacetone (MW = 100.12, 1.5 equiv, 1.5 mmol, density = 0.980 g/mL, V = 153 µL) in the stated order. The acetylacetone was added within 30 seconds after the addition of cyclohexanone. The reaction stirred at 25 °C. At 72 h, an aliquot was removed and a ^{1}H NMR was recorded. The spectrum is seen below.

According to Yang, aldol product **4b** in DMSO-d$_6$ (see page S12 within the Supporting Information document of: Wang, R.; Xu, E.; Su, Z.; Duan, H.; Wang, J.; Xue, L.; Lin, Y.; Li, Y.; Weia, Z.; Yang, Q. *RSC Adv.,* **2018**, *8*, 28376–28385), shows, among others, two resonance patterns between (i) 2.70 to 2.75 ppm and (ii) 8.18 to 8.22 ppm. However, in the below spectrum no resonance pattern is noted between 2.70 to 2.75 ppm, while within the 8.18 to 8.22 ppm region a minute resonance is noted. Comparison of that resonance centered at 8.17 ppm (possible aldol product), integration value of 0.07, with the one centered at 8.04 (Knoevenagel product), integration value of 1.17, a chemoselectivity of 17:1 (Knoevenagel/aldol) is obtained.

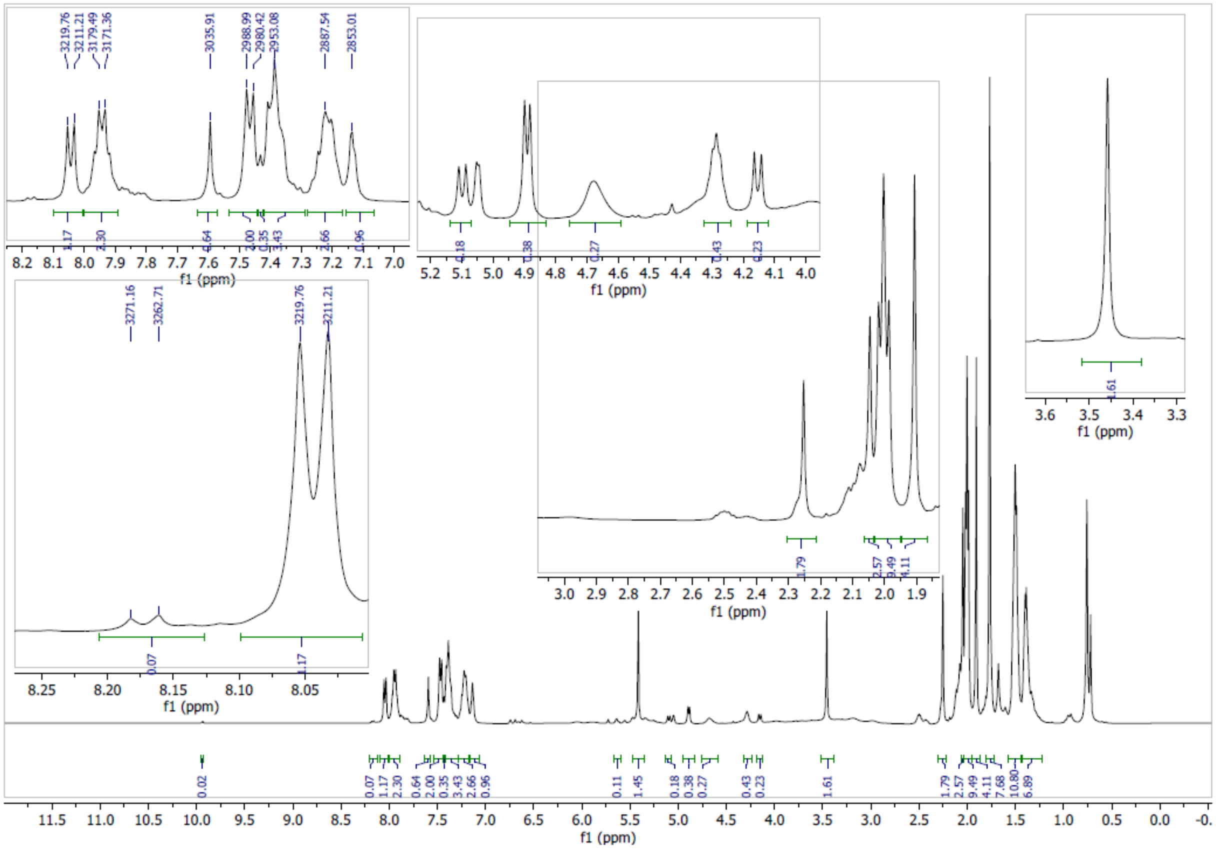

Supplement: Supplementary file 1 [file molecules-29-00004-s001.zip › molecules-2759211-supplementary.pdf]
